# Asymmetric organocatalytic Michael addition of cyclopentane-1,2-dione to alkylidene oxindole

**DOI:** 10.3762/bjoc.18.18

**Published:** 2022-02-03

**Authors:** Estelle Silm, Ivar Järving, Tõnis Kanger

**Affiliations:** 1 Department of Chemistry and Biotechnology, Tallinn University of Technology, Akadeemia tee 15, 12618 Tallinn, Estonia

**Keywords:** cyclopentane-1,2-dione, enantioselective catalysis, Michael addition, organocatalysis, squaramide

## Abstract

An asymmetric Michael reaction between cyclopentane-1,2-dione and alkylidene oxindole was studied in the presence of a multifunctional squaramide catalyst. Michael adducts were obtained in high enantioselectivities and in moderate diastereoselectivities.

## Introduction

Diketones are generally very versatile starting materials in organic synthesis [1–2]. Specifically, due to their keto–enol tautomerism and high reactivity, diketones are excellent precursors for different pharmaceuticals [3]. Cyclic 1,3-diketones have been widely exploited to access enantiomerically enriched scaffolds with increased molecular complexity. There are many examples of the organocatalytic synthesis of fused cycles starting from the cyclohexane-1,3-dione. For example, Rueping et al. demonstrated that the cyclohexane-1,3-dione undergoes a cascade reaction with α,β-unsaturated aldehydes [4] and they later employed the method to synthesise indoloquinolizidines [5]. Moreover, six-membered and five-membered cyclic 1,3-diketones have been investigated in reactions with acetates of nitroalkenes [6], cyanoacrylates and benzylidene malononitriles [7], *ortho*-hydroxy-benzhydryl alcohols [8], α,β-unsaturated pyrazolamides [9] and, 2-oxobut-3-enoates [10]. A 1,2-dicarbonyl moiety is also an important structural fragment present in various natural products and biologically active compounds [11]. 1,2-Diketones have been used for the synthesis of photosensitive polymers [12] and substituted imidazoles [13–14] and have been used in carbohydrate chemistry [15]. Cyclic six-membered 1,2-diketones have been shown to react with benzylidene malononitriles [7,16], β-nitrostyrenes [17] and substituted propionaldehydes [18]. For a while, there were no examples related to cyclopentane-1,2-dione (CPD). In 2004, the first instance of using CPD as a precursor for high value-added fine chemicals such as a homocitric acid lactone was published by our group [19]. Since then we have developed synthetic pathways for lycoperdic acid [20] and nucleoside analogues [21] starting from CPD. The organocatalytic methods for the synthesis of substituted cyclopentane diones were uninvestigated until 2014 when we showed that CPD undergoes a Michael addition with nitrostyrenes [22]. Subsequently, different cascade reactions for CPD have been developed: with highly reactive (*E*)-2-oxobut-3-enoates [23], α,β-unsaturated aldehydes [24] and alkylidene malonates [25].

Herein, we report the results of an asymmetric organocatalytic Michael addition of CPD to alkylidene oxindoles.

## Results and Discussion

**Figure 1:**
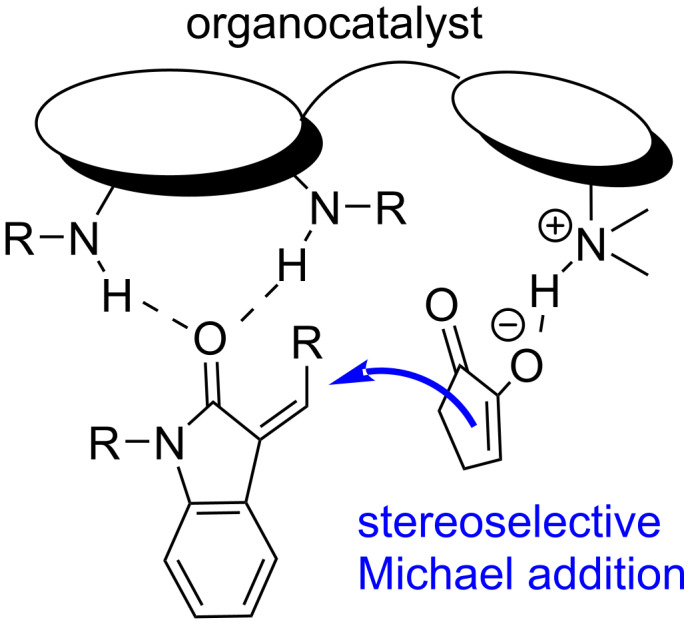
Model of the catalyst action.

Chiral multifunctional thioureas [26–27] and squaramides [28] are extensively used as catalysts in asymmetric Michael additions. We believed that a bifunctional hydrogen-bonding catalyst would activate both CPD via a tertiary amino group of a quinuclidine moiety acting as a base via anion-binding, and an oxindole through the squaramide or thiourea moieties of the catalyst as hydrogen bond donors (Figure 1) [29–32].

**Figure 2:**
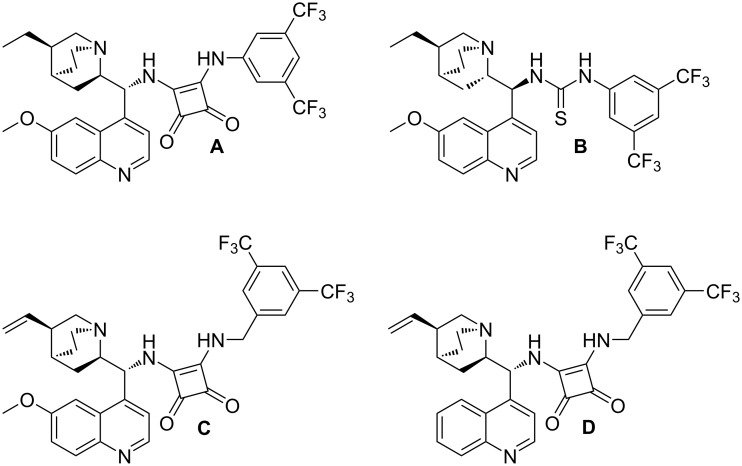
Catalysts screened.

Therefore, squaramide and thiourea catalysts were screened in a model reaction between CPD **1** and Boc-protected benzylidene oxindole **2a** at room temperature in the presence of 10 mol % of catalyst (Figure 2).

First, the quinidine-derived squaramide **A** was used and the desired product was obtained as a mixture of chromatographically inseparable diastereoisomers in 53% yield but in low enantiomeric excess for both diastereomers (Table 1, entry 1). With the quinine-derived thiourea **B**, the reaction was slow and the yield was very low, 12% (Table 1, entry 2). For that reason, we focused on the screening of squaramides. Squaramides were found to be more selective catalysts than thioureas. When squaramide **C** was used as a catalyst, the product was isolated in 80%/87% ee (major/minor diastereoisomer) (Table 1, entry 3). The enantioselectivity was even higher with the cinchonine-derived squaramide **D**, 85%/92% (major/minor) (Table 1, entry 4). To further optimise the reaction, we screened different solvents (apolar, polar aprotic, and chlorinated solvents) (Table 1, entries 5–7). According to the obtained results chloroform was clearly superior to other solvents. Previously the isolated yield of the product had been moderate and to increase the yield the substrate concentration was varied. A substantial excess of CPD (five equivalents) led to a very slow reaction and a decrease in enantioselectivity (Table 1, entry 8). It was assumed that the binding between CPD and the catalyst was stronger than the binding between the substituted oxindole and the squaramide decreasing the effective concentration of the catalyst. Taking this into consideration, 2 equiv of substituted oxindole was used and the reaction proceeded smoothly in 2 h in high enantioselectivity (90%/94% ee), in high yield (74%) but in moderate diastereoselectivity (Table 1, entry 9). Next, we looked onto the effect of lower temperature on the reaction. At 0 °C the reaction was approximately 10 times slower and only the ee of the minor diastereoisomer increased by 3% (Table 1, entry 10), so there was no justification for carrying out the reaction at a lower temperature because of the longer time needed.

**Table 1:** Screening conditions for the reaction^a^.

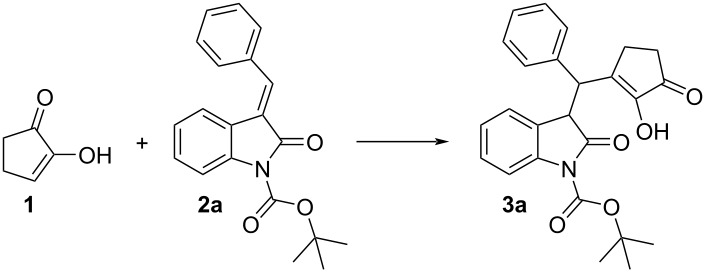							

Entry	Catalyst	Solvent	Temp.	Time	Yield^b^	dr	ee%^c^ (major/minor)

1	**A**	chloroform	rt	2 h	53%	3:1	43/27
2	**B**	chloroform	rt	6 days	12%	3.8:1	−57/−67
3	**C**	chloroform	rt	7 h	59%	2.4:1	80/87
4	**D**	chloroform	rt	2.5 h	58%	3:1	85/92
5^d^	**D**	toluene	rt	2 days	54%	2.5:1	77/81
6^d^	**D**	THF	rt	2 days	44%	2.6:1	68/74
7^d^	**D**	DCM	rt	1 day	51%	2.5:1	83/83
8^e^	**D**	chloroform	rt	12 days	59%	2.2:1	71/87
9^f^	**D**	chloroform	rt	2 h	74%	2.6:1	90/94
10^f^	**D**	chloroform	0 °C	23 h	75%	2.7:1	90/97

^a^Reaction conditions: 0.2 M solution of **1** (1 equiv), **2** (1 equiv), catalyst (0.1 equiv). ^b^Isolated yield after column chromatography. ^c^ee determined by chiral HPLC analysis. ^d^1.5 equiv of **1**; ^e^5 equiv of **1**; ^f^2 equiv of **2a**.

**Scheme 1:**
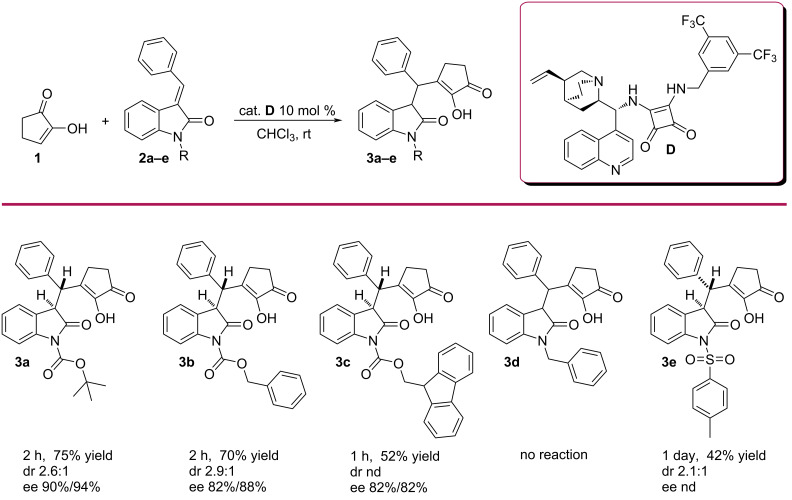
Screening of different *N*-protecting groups. Reaction conditions: 0.2 M solution of **1** (1 equiv), **2** (1 equiv), of catalyst **D** (0.1 equiv), chloroform, at room temperature; isolated yields after column chromatography; ee determined by chiral HPLC.

Next, we screened different protecting groups for the oxindole. Previously, Boc-protected oxindole **2a** gave us the product in 75% yield, in dr 2.6:1 and in ee 90%/94% (Scheme 1, **3a**). With a Cbz-protecting group the enantioselectivity decreased to 82%/88% (Scheme 1, **3b**). The use of a sterically more demanding Fmoc-protecting group decreased the ee values even more for the minor diastereoisomer (Scheme 1, **3c**). Surprisingly, with benzyl-protected oxindole, the reaction did not proceed (Scheme 1, **3d**), which implies that the carbonyl group of the carbamate moiety in the *N*-protecting group and electron-withdrawing properties of the protection groups are essential for coordination with the catalyst and for the reactivity of the Michael acceptor. Using a tosyl-protected oxindole the reaction was sluggish, the yield was low and the enantioselectivity could not be determined (Scheme 1, **3e**). These experiments revealed that the best results were achieved in chloroform at room temperature with catalyst **D**, using 1 equiv of diketone and 2 equiv of *N*-Boc-substituted oxindole **2a**.

**Scheme 2:**
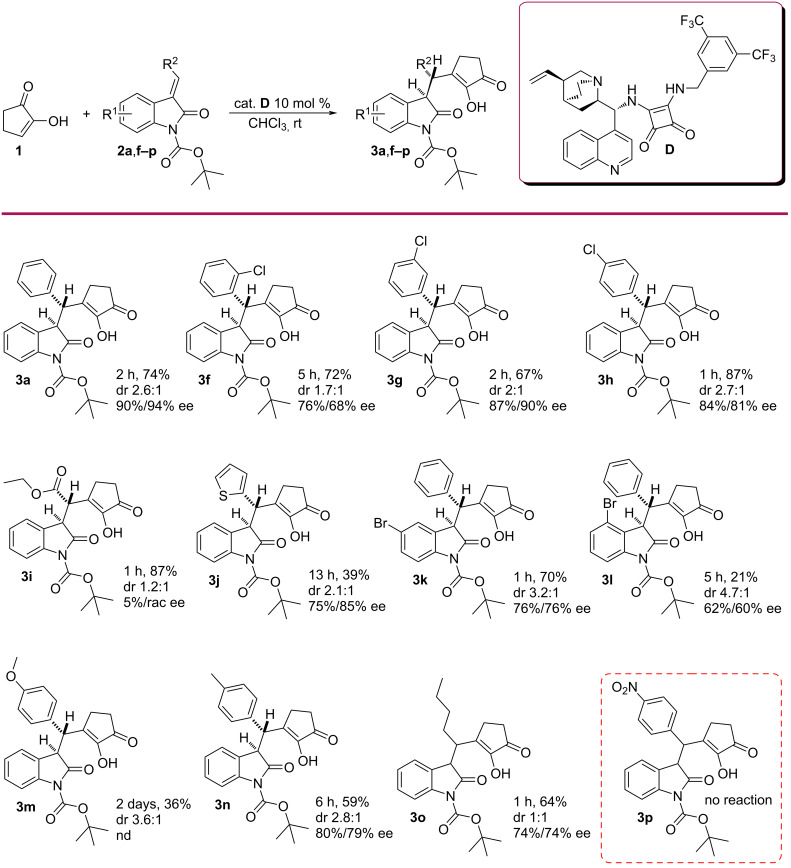
Scope of the reaction (the relative configuration of the major diastereoisomer is depicted). Reaction conditions: 0.2 M solution of 1 equiv of **1**, 2 equiv of **2**, 0.1 equiv of catalyst **D**, chloroform, at room temperature; isolated yields after column chromatography; ee determined by chiral HPLC.

**Scheme 3:**
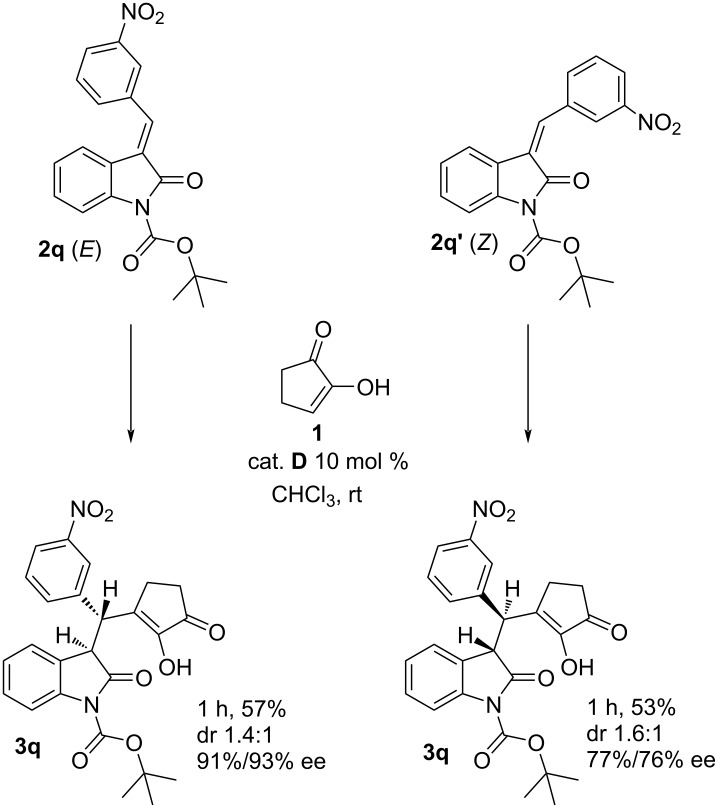
Comparison reactions of *E*- and *Z*-isomers (the relative configurations of the major diastereoisomers are depicted). Reaction conditions: 0.2 M solution of 1 equiv of **1**, 2 equiv of **2**, 0.1 equiv of catalyst **D**, chloroform, at room temperature; isolated yields after column chromatography; ee determined by chiral HPLC.

Under optimised conditions, the substrate scope of the reaction was examined by using various substituted oxindoles with an *E*-configuration of the double bond. The results are presented in Scheme 2. Both electron-withdrawing (Scheme 2, **3f**–**h**) and electron-donating groups (Scheme 2, **3m**,**n**) at the phenyl ring of the benzylidene moiety were tolerated. The position of the halide at the aromatic ring did not have a major effect on the yield or the enantioselectivity. *Ortho*-, *meta*- and *para*-chlorophenyl-substituted starting materials afforded products in similar enantioselectivities (Scheme 2, **3f**–**h**). However, the reaction was slower with the sterically more hindered *ortho*-chloro substrate (Scheme 2, **3f**). When instead of a benzylidene-containing substrate an alkylidene with an extra ester moiety was used, the enantioselectivity was lost and the product **3i** was obtained as a racemic mixture. Either additional coordination with the catalyst or a lack of π–π-interaction may have been responsible for that. Also, a higher C–H acidity of the proton at the stereogenic centre and possible racemisation can’t be excluded. A heteroaromatic oxindole derivative afforded the product **3j** in lower yield and high ee values. 4- and 5-bromo oxindole derivatives (**2l** and **2k**, respectively) were also used as starting compounds. If the substituent in the oxindole ring was further from the reaction centre, the outcome was not affected (Scheme 2, **3k**). However, when using a 4-bromo-substituted oxindole, the reaction was slower, the yield drastically decreased and the enantioselectivity was moderate (Scheme 2, **3l**). The reaction with an electron-donating *p*-MeO-substituted benzylidene oxindole was very sluggish and did not reach full conversion (Scheme 2, **3m**). The product **3m** was obtained with only 36% yield and with undetermined enantiomeric purity, since the peaks were not separable in various HPLC methods. Similarly, the *p-*Me-substituted oxindole was also slow in reacting and the yield was moderate, but the enantioselectivity remained high (Scheme 2, **3n**). The reaction tolerated alkylidene oxindoles, although the product was obtained in a slightly lower yield and enantioselectivity (Scheme 2, **3o**). The reaction did not occur when starting compound **2p** was tried. This was probably because of the very poor solubility of the starting material. Generally, the diastereoselectivities of the reactions were moderate (dr 2.1:1–3.6:1) throughout the scope. The diastereoselectivity was missing or was very low for the compounds with non-aromatic substituents at the double bond (**3i** and **3o**).

The relative *anti*-configuration of the vicinal diastereotopic hydrogens was determined by comparing the ^3^*J*_HH_ coupling constants of the major diastereomer with those of the minor diastereomer. The constants were larger for the major diastereomer, meaning vicinal hydrogens were in *anti*-configuration.

In all previous experiments only *E*-isomers were used. In the case of the 3-nitro-substituted starting material **2q** we managed to separate isomers and carried out the reaction with both the *E*- and *Z*-isomer. In these experiments, both isomers afforded the same major diastereoisomer but opposite enantiomers (Scheme 3, **3q**). The diastereoselectivities were similar for the isomers.

Since the diastereoselectivity of the reaction was low, we attempted to increase the ratio of diastereoisomers via enolisation followed by diastereoselective protonation (Table 2). As the racemate of **3a** was obtained in a higher diastereomeric ratio (6.3:1) we applied kinetic and thermodynamic conditions for the epimerisation of it (Table 2, entries 1 and 2, respectively). Unfortunately, in both cases the diastereomeric ratio decreased and the amount of more stable *syn* diastereoisomer increased. A similar trend was observed when starting from the enantiomerically enriched **3a** (Table 2, entry 3).

**Table 2:** Epimerisation of **3a**.

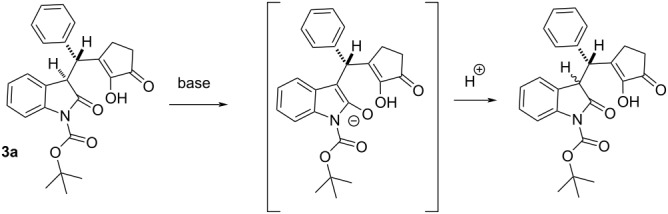				

Entry	Starting compound		Conditions	Product (dr)

		dr		

1	**3a** (rac)	6.3:1	LDA, THF; −78 °C, 30 min, then sat. aq. NH_4_Cl	2.7:1
2	**3a** (rac)	6.3:1	*t*-BuOK/*t-*BuOH, rt, overnight	2.6:1
3	**3a**	2.5:1	LiHDMS, THF; −78 °C, 30 min, then sat. aq. NH_4_Cl	2.2:1

It has been shown that substituted oxindoles can be converted to indolopyrans via intramolecular cyclisation [33]. We also tried synthesizing 4*H*-pyrans in acidic conditions but no cyclised product was detected.

## Conclusion

In summary, we have developed a new asymmetric organocatalytic Michael addition of cyclopentane-1,2-dione to alkylidene oxindoles catalysed by bifunctional squaramide which leads to products in high enantioselectivities and moderate diastereoselectivities. The scope of alkylidene oxindoles is reasonably wide including aromatic and aliphatic substituents at the double bond and also substituents in the oxindole core. The work widens the synthetic utility of cyclopentane-1,2-diones.

## Supporting Information

File 1 Experimental details, NMR spectra, HPLC chromatograms.
