# Supplementary material for: Asymmetric organocatalytic Michael addition of cyclopentane-1,2-dione to alkylidene oxindole
Source: Beilstein J Org Chem. 2022 Feb 3;18:167–73. doi: 10.3762/bjoc.18.18 (PMC8822468; doi:10.3762/bjoc.18.18)
Supplement: File 1 — Experimental details, NMR spectra, HPLC chromatograms. [file Beilstein_J_Org_Chem-18-167-s001.pdf]

BEILSTEIN JOURNAL OF ORGANIC CHEMISTRY

Supporting Information

for

Asymmetric organocatalytic Michael addition of cyclopentane-1,2-dione to alkylidene oxindole

Estelle Silm, Ivar Järving and Tõnis Kanger

Beilstein J. Org. Chem. **2022,** *18,* 167–173. doi:10.3762/bjoc.18.18

Experimental details, NMR spectra, HPLC chromatograms

License and Terms: This is a supporting information file under the terms of the Creative Commons Attribution License (https://creativecommons.org/licenses/by/4.0). Please note that the reuse, redistribution and reproduction in particular requires that the author(s) and source are credited and that individual graphics may be subject to special legal provisions.
The license is subject to the *Beilstein Journal of Organic Chemistry* terms and conditions: (https://www.beilstein-journals.org/bjoc/terms)

Contents

1. General information S2

Synthesis of starting materials S3

General procedure I for the synthesis of compounds **3** S8

2. ^{1}H NMR and ^{13}C NMR spectra S27

3. HPLC chromatograms S49

1. General information

Full assignment of ^{1}H and ^{13}C chemical shifts were based on the 1D and 2D FT NMR spectra measured on a Bruker Avance III 400 MHz instrument. Residual solvent signals were used ($CDCl_3$ δ = 7.26 ^{1}H NMR, 77.2 ^{13}C NMR and $(CD_3)_2SO$ δ = 2.5 ^{1}H NMR, δ = 39.5 ^{13}C NMR) as internal standards. High-resolution mass spectra were recorded by using Agilent Technologies 6540 UHD Accurate-Mass Q-TOF LC/MS spectrometer by using AJ-ESI ionization. IR spectra were recorded on a Bruker Tensor 27 FT-IR spectrophotometer. Chiral HPLC was performed by using CHIRALPAK® AD-H column. Precoated silica gel 60 F254 plates were used for TLC. Commercal reagents and solvents were generally used as received. Chloroform and ethyl acetate was distilled over phosphorus pentoxide.

Racemic compounds were prepared following the general procedure using DABCO, triethylamine or K_2CO_3 as catalyst.

Cyclopentane-1,2-dione **1** was prepared in a manner analogous to literature [1] from commercially available cyclopentanone. Alkylidene oxindoles **2** were prepared from commercially available oxindole or isatin and commercially available aldehydes in a manner similar to literature [2] and the analytical data matched with that of the literature [3, 4]. Catalysts **A**, **C**, **D** [5, 6, 7], **B** [8] were prepared in a manner analogous to literature and the analytical data matched with that of the literature.

Synthesis of starting materials

Benzyl (*E*)-3-benzylidene-2-oxoindoline-1-carboxylate (2b)

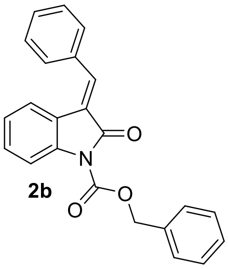

To the oxindole (10 mmol, 1 equiv) and benzaldehyde (10 mmol, 1 equiv) in methanol (0.3 M) was added piperidine (10 mmol, 1 equiv) and the reaction mixture was stirred under reflux. After 30 min, the reaction mixture was cooled to room temperature. The benzylidene oxindole (*E*)-**S2a** precipitated, the reaction mixture was then filtered and recrystallized to obtain the (*E*)-**S2a** in 64% yield (1.4 g).

Benzylidene oxindole **S2a** (1.36 mmol, 1 equiv) and DMAP (0.07 mmol, 0.05 equiv) was dissolved in DCM (0.3 M). Then under Ar Et_3N (1.63 mmol, 1.2 equiv) followed by benzyl chloroformate (1.63 mmol, 1.2 equiv) was added. The reaction mixture was stirred at room temperature and monitored by TLC and NMR. After 3 h, the reaction was quenched with water. The organic layer was exctracted with DCM (3 × 4 mL). The product was purified by column chromatography (EtOAc/

hexane 5%→20%) affording the product **2b** as yellow solid in 48% yield (233 mg). The product was further purified by recrystallization, affording the product **2b** as yellow needles in 60% yield (140 mg).

^{1}H NMR (400 MHz, $CDCl_3$) δ 7.99 (d, *J* = 8.5 Hz, 1H), 7.91 (s, 1H), 7.68 (d, *J* = 7.7 Hz, 1H), 7.66-7.60 (m, 2H), 7.58-7.52 (m, 2H), 7.51-7.28 (m, 7H), 7.01 (td, *J* = 7.7, 1.1 Hz, 1H), 5.49 (s, 2H).

^{13}C NMR (101 MHz, $CDCl_3$) δ 166.5, 151.0, 139.7, 139.0, 135.2, 134.6, 132.3, 130.3, 130.1, 129.3 (2C), 128.9 (2C), 128.8 (2C), 128.6, 128.2 (2C), 125.9, 124.2, 122.0, 115.5, 68.7.

IR: 3061, 3021, 1768, 1752, 1693, 1633, 1602, 1462, 1384, 1335, 1305, 1291, 1235, 1165, 1093, 1060, 779, 750, 696, 568, 459 cm^{-1}.

HRMS (ESI): m/z $[M+H]^+$ calcd for $[C_{23}H_{18}NO_3]^+$: 356.1281; found: 356.1274.

(9*H*-Fluoren-9-yl)methyl (*E*)-3-benzylidene-2-oxoindoline-1-carboxylate (2c)

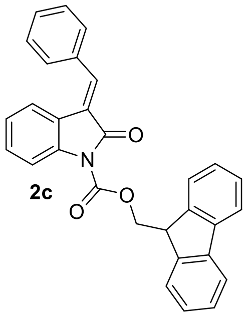

2c

To the oxindole (10 mmol, 1 equiv) and benzaldehyde (10 mmol, 1 equiv) in methanol (0.3 M) was added piperidine (10 mmol, 1 equiv) and the reaction mixture was stirred under reflux. After 30 min, the reaction mixture was cooled to room temperature. The benzylidene oxindole (*E*)-**S2a** precipitated, the reaction mixture was then filtered and recrystallized to obtain the (*E*)-**S2a** in 64% yield (1.4 g).

A dry and argon-flushed round-bottomed flask was charged with benzylidene oxindole **S2a** (0.9 mmol, 1 equiv) in anhydrous THF (0.9 M) and then NaH (1.35 mmol, 1.5 equiv) and FmocCl (1.35 mmol, 1.5 equiv) were added to the flask at 0 °C and stirred overnight at room temperature. Then the reaction mixture was concentrated in vacuo and the residue was purified by column chromatography (EtOAc/hexane 10%→14%) affording the product **2c** as yellow solid in 49% yield (196 mg). The product was further purified by recrystallization, affording the product **2c** in 79% yield (155 mg).

^1H NMR (400 MHz, $CDCl_3$) δ 7.97 (s, 1H), 7.87-7.83 (m, 2H), 7.82-7.76 (m, 3H), 7.72-7.68 (m, 1H), 7.68-7.63 (m, 2H), 7.52-7.41 (m, 5H), 7.37 (td, *J* = 7.4, 1.2 Hz, 2H), 7.30-7.21 (m, 1H), 7.00 (td, *J* = 7.7, 1.1 Hz, 1H), 4.72 (d, *J* = 7.1 Hz, 2H), 4.46 (t, *J* = 7.1 Hz).

^{13}C NMR (101 MHz, $CDCl_3$) δ 166.5, 151.1, 143.6 (2C), 141.5 (2C), 139.7, 139.0, 134.6, 130.4, 130.1, 129.3 (2C), 128.9 (2C), 128.1 (2C), 127.5 (2C), 126.0, 125.6 (2C), 124.2, 122.5, 121.8, 120.2 (2C), 115.5, 69.2, 46.8.

IR: 3066, 3023, 1783, 1775, 1631, 1602, 1463, 1450, 1386, 1333, 1306, 1290, 1244, 1165, 1093, 1061, 1037, 1017, 934, 780, 758, 696, 621, 570, 549, 459 cm^{-1}.

HRMS (ESI): m/z $[M+Na]^+$ calcd for $[C_{30}H_{21}NO_3Na]^+$: 466.1414; found: 466.1408.

tert-Butyl (_E_)-2-oxo-3-(thiophen-2-ylmethylene)indoline-1-carboxylate (2j)

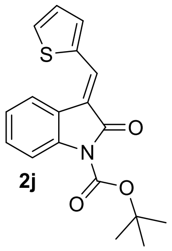

To the oxindole (2 mmol, 1 equiv) and thiophene-2-carbaldehyde (2 mmol, 1 equiv) in methanol (0.3 M) was added piperidine (2 mmol, 1 equiv) and the reaction mixture was stirred under reflux. After 30 min, the reaction mixture was cooled to room temperature. The alkylidene oxindole **S2j** precipitated, the reaction mixture was then filtered. The intermediate (*E*)-**S2j** was obtained in 68% yield (310 mg).

(*E*)-3-(Thiophen-2-ylmethylene)indolin-2-one (**S2j**, 1.36 mmol, 1 equiv) and DMAP (0.068 mmol, 0.05 equiv) were dissolved in THF (0.26 M). Then Boc_2O (1.49 mmol, 1.1 equiv) solution in THF (0.89 M) was added to the reaction mixture under argon. The mixture was stirred (TLC and/or NMR monitoring) and after 3 h, the mixture was concentrated in vacuo and purified by column chromatography (EtOAc/hexane 7%→10%) affording the product **2j** as a yellow solid in 93% yield (412 mg).

^{1}H NMR (400 MHz, $CDCl_3$) δ 7.85-7.83 (m, 1H), 7.83-7.82 (m, 1H), 7.76 (s, 1H), 7.68 (dt, *J* = 5.1, 1.1 Hz, 1H), 7.56 (dd, *J* = 7.6, 0.8 Hz, 1H), 7.34-7.28 (m, 1H), 7.21-7.15 (m, 2H), 1.69 (s, 9H).

^{13}C NMR (101 MHz, $CDCl_3$) δ 165.1, 149.6, 138.1, 137.9, 137.7, 134.6, 129.4, 128.8, 127.6, 124.3, 124.0, 119.9, 118.4, 115.2, 84.3, 28.3 (3C).

IR: 3081, 2984, 1723, 1608, 1466, 1415, 1370, 1351, 1325, 1298, 1253, 1149, 1092, 1064, 1004, 835, 786, 759, 743, 589, 504 cm^{-1}.

HRMS (ESI): m/z $[M+Na]^+$ calcd for $[C_{18}H_{17}NO_3SNa]^+$: 350.0821; found: 350.0818.

***tert*-Butyl (*E*)-3-benzylidene-4-bromo-2-oxoindoline-1-carboxylate (2l)**

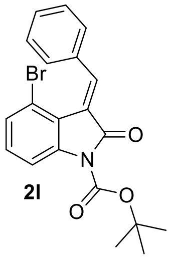

2l

An oven dried 50 mL round-bottomed flask under argon atmosphere was charged with benzyltriphenylphosphonium bromide (1.3 mmol, 1 equiv). Anhydrous THF (4 mL) was then added via syringe and the solution is cooled to 0 °C. A solution of butyl lithium (2.5 M in hexanes, 183 µL, 1.3 mmol, 1 equiv) was added dropwise via syringe and the reaction was allowed to stir for 1 h in order to form the Wittig reagent. A solution of isatin (1.3 mmol, 1 equiv) in dry THF (4 mL) was added dropwise via syringe at 0 °C and the resulting solution was allowed to stir at room temperature for 15 h. The reaction mixture was then poured into a saturated solution of NH_4Cl (4 mL) at 0 °C and extracted with DCM (3 × 3 mL). The combined organic layers were dried over $MgSO_4$, filtered and concentrated in vacuo. The mixture was purified by column chromatography (EtOAc/DCM 5%→10%) affording the product (*E*)-**S2l** as yellow solid in 26% yield (100 mg).

(*E*)-3-Benzylidene-4-bromoindolin-2-one (**S2l**, 0.33 mmol, 1 equiv) and DMAP (0.017 mmol, 0.05 equiv) were dissolved in THF (0.26 M). Then Boc_2O (0.36 mmol, 1.1 equiv) solution in THF (0.89 M) was added to the reaction mixture under argon. The mixture was stirred (TLC and/or NMR monitoring) and after 2 h, the mixture was concentrated *in vacuo* and purified by column chromatography (EtOAc/petroleum ether 10%) affording the product **2l** as a yellow solid in 62% yield (83 mg).

^{1}H NMR (400 MHz, $CDCl_3$) δ 8.87 (s, 1H), 8.03-7.94 (m, 2H), 7.87 (dd, *J* = 8.1, 1.0 Hz, 1H), 7.47-7.39 (m, 3H), 7.36 (dd, *J* = 8.1, 1.0 Hz, 1H), 7.14 (t, *J* = 8.2 Hz, 1H), 1.64 (s, 9H).

^{13}C NMR (101 MHz, $CDCl_3$) δ 163.4, 149.63, 143.4, 140.5, 133.5, 132.0 (2C), 130.7, 129.7, 129.4, 128.2 (2C), 124.7, 121.6, 116.6, 113.9, 84.8, 28.3 (3C).

IR: 2981, 2934, 1826, 1771, 1732, 1591, 1434, 1394, 1369, 1339, 1295, 1254, 1158, 1115, 935, 846, 777, 690, 601 cm^{-1}.

HRMS (ESI): m/z $[M+Na]^+$ calcd for $[C_{20}H_{18}NBrO_3Na]^+$: 422.0362; found: 422.0357.

***tert*-Butyl (*E*)-2-oxo-3-pentylideneindoline-1-carboxylate (2o)**

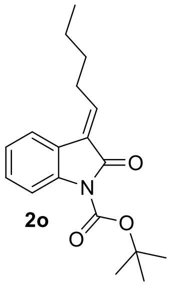

2o

To the oxindole (2.25 mmol, 1 equiv) and pentanal (2.25 mmol, 1 equiv) in methanol (0.2 M) was added piperidine (2.25 mmol, 1 equiv) and the reaction mixture was stirred under reflux. After 2 h, the reaction mixture was cooled to room temperature. The reaction mixture was concentrated in vacuo and purified by column chromatography (EtOAc/DCM 5%→20%). The intermediate (*E*)-**S2o** was obtained in 58% yield (264 mg).

(*E*)-3-Pentylideneindolin-2-one (**S2o**, 1.24 mmol, 1 equiv) and DMAP (0.06 mmol, 0.05 equiv) were dissolved in THF (0.26 M). Then Boc_2O (1.36 mmol, 1.1 equiv) solution in THF (0.89 M) was added to the reaction mixture under argon. The mixture was stirred (TLC and/or NMR monitoring) and after 1 h, the mixture was concentrated in vacuo and purified by column chromatography (EtOAc/DCM 0%→10%). affording the product **2o** as a yellow oil in 45% yield (168 mg).

^{1}H NMR (400 MHz, $CDCl_3$) δ 7.91 (d, *J* = 8.2 Hz, 1H), 7.6 (d, *J* = 7.6 Hz, 1H), 7.31 (td, *J* = 7.9, 1.3 Hz, 1H), 7.16 (td, *J* = 7.6, 1.1 Hz, 1H), 7.09 (td, *J* = 7.7 Hz, 1H), 2.68 (q, *J* = 7.5 Hz, 2H), 1.65 (s, 9H), 1.64-1.56 (m, 2H), 1.52-1.40 (m, 2H), 0.96 (t, *J* = 7.3 Hz, 3H).

^{13}C NMR (101 MHz, $CDCl_3$) δ 166.2, 149.6, 144.0, 139.5, 129.1, 126.6, 124.1, 123.3, 122.8, 115.2, 84.2, 30.8, 29.2, 28.3 (3C), 22.7, 14.0.

IR: 3305, 2961, 2933, 2873, 1830, 1736, 1654, 1608, 1583, 1527, 1466, 1394, 1370, 1344, 1291, 1253, 1151, 1050, 983, 841, 754 cm^{-1}.

HRMS (ESI): m/z $[M+Na]^+$ calcd for $[C_{18}H_{23}NO_3Na]^+$: 324.1570; found: 324.1569.

General procedure I for the synthesis of compounds 3

To a solution of cyclopentane-1,2-dione (**1**, 0.1 mmol, 1 equiv) and *N*-Boc alkylidene oxindole **2** (0.2 mmol, 2 equiv) in chloroform (510 µL, 0.2 M) was added cinchonine derived squaramide catalyst **D** (0.01 mmol, 0.1 equiv). The mixture was stirred until the reaction was completed (TLC and/or NMR monitoring). The mixture was purified by column chromatography (EtOAc/DCM 5%→10%) affording the product **3** as a mixture of unseparable diastereoisomers.

***tert*-Butyl (*R**)-3-((*R**)(2-hydroxy-3-oxocyclopent-1-en-1-yl)(phenyl)methyl)-2-oxoindolin-1-carboxylate (3a)**

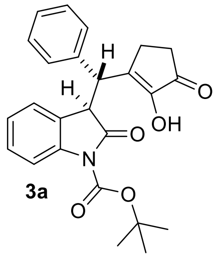

3a

Synthesized according to general procedure I, using cyclopentane-1,2-dione (**1**), *tert*-butyl (*E*)-3-benzylidene-2-oxoindoline-1-carboxylate (**2a**) and catalyst **D**. Compound **3a** was obtained as an off-white solid in 74% yield (31.6 mg, dr 2.6:1). Major diastereoisomer: ee 90% [HPLC (CHIRALPAK® AD-H, hexane/ethanol 95:5, 25 °C, 1 mL/min, 254 nm): t_R (major) = 25.8 min, t_R (minor) = 38.8 min].

^{1}H NMR (400 MHz, $CDCl_3$) δ 7.71 (d, *J* = 8.2 Hz, 1H), 7.33-7.16 (m, 6H), 6.87 (t, *J* = 7.6 Hz, 1H), 6.40 (d, *J* = 7.6 Hz, 1H), 5.94 (b.s, 1H), 4.58 (d, *J* = 9.4 Hz, 1H), 4.21 (d, *J* = 9.4 Hz, 1H), 2.76-2.65 (m, 1H), 2.48-2.37 (m, 3H), 1.61 (s, 9H).

^{13}C NMR (101 MHz, $CDCl_3$) δ 203.2, 173.9, 148.7, 149.1, 144.4, 140.32, 138.1, 129.2 (2C), 128.9 (2C), 128.76, 128.6, 126.1, 125.3, 123.9, 114.8, 84.5, 48.9, 47.4, 31.9, 28.22 (3C), 24.6.

Minor diastereoisomer: ee 94% [HPLC (CHIRALPAK® AD-H, hexane/ethanol 95:5, 25 °C, 1 mL/min, 254 nm): t_R (major) = 35.2 min, t_R (minor) = 31.2 min].

^{1}H NMR (400 MHz, $CDCl_3$) δ 7.65 (d, *J* = 8.0 Hz, 1H), 7.33-7.16 (m, 5H), 7.12 (d, *J* = 7.5 Hz, 1H), 7.10-7.05 (m, 2H), 6.97 (b.s, 1H), 4.50 (d, *J* = 4.8 Hz, 1H), 4.45 (d, *J* = 4.7 Hz, 1H), 2.54-2.48 (m, 1H), 2.48-2.37 (m, 2H), 2.36-2.28 (m, 1H), 1.57 (s, 9H).

^{13}C NMR (101 MHz, $CDCl_3$) δ 203.3, 175.4, 149.6, 148.8, 143.7, 140.28, 136.7, 128.72, 128.5 (2C), 127.9 (2C), 127.7, 126.3, 124.5, 124.2, 115.0, 84.7, 49.1, 48.8, 32.0, 28.16 (3C), 25.9.

IR: 3278, 2979, 2957, 1769, 1723, 1698, 1658, 1604, 1479, 1464, 1414, 1393, 1370, 1349, 1255, 1150, 1094, 753, 699 cm^{-1}.

HRMS (ESI): m/z $[M+Na]^+$ calcd for $[C_{25}H_{25}NO_5Na]^+$: 442.1625; found: 442.1622.

Benzyl (*R)-3-((*R**)-(2-hydroxy-3-oxocyclopent-1-en-1-yl)(phenyl)methyl)-2-oxoindoline-1-carbocylate (3b)**

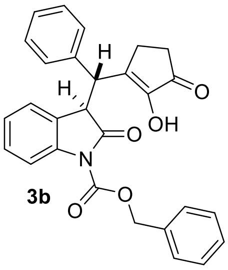

3b

Synthesized according to general procedure I, using cyclopentane-1,2-dione (**1**), benzyl (*E*)-3-benzylidene-2-oxoindoline-1-carboxylate (**2b**) and catalyst **D**. Compound **3b** was obtained as an yellow solid in 70% (32 mg, dr 2.9:1). <u>Major diastereoisomer</u>: ee 82% [HPLC (CHIRALPAK® AD-H, hexane/isopropanol 9:1, 25 °C, 1 mL/min, 210 nm): t_R (major) = 75.6 min, t_R (minor) = 84.9 min].

^{1}H NMR (400 MHz, $CDCl_3$) δ 7.80 (d, *J* = 8.2 Hz, 1H), 7.50-7.07 (m, 11H), 6.89 (td, *J* = 7.6, 1.1 Hz, 1H), 6.43 (d, *J* = 7.6 Hz, 1H), 5.96 (b.s, 1H), 5.41 (d, *J* = 3.7 Hz, 2H), 4.64 (d, *J* = 9.2 Hz, 1H), 4.22 (d, *J* = 9.3 Hz, 1H), 2.73-2.62 (m, 1H), 2.49-2.35 (m, 3H).

^{13}C NMR (101 MHz, $CDCl_3$) δ 203.20, 173.7, 150.8, 148.8, 144.1, 139.89, 137.9, 135.12, 129.2 (2C), 128.9 (2C), 128.79 (4C), 128.3 (2C), 128.0, 126.1, 125.3, 124.3, 115.1, 68.68, 49.0, 47.5, 31.9, 24.7.

<u>Minor diastereoisomer</u>: ee 88% [HPLC (CHIRALPAK® AD-H, hexane:isopropanol 9:1, 25 °C, 1 mL/min, 210 nm): t_R (major) = 117.6 min, t_R (minor) = 71.0 min].

^{1}H NMR (400 MHz, $CDCl_3$) δ 7.75 (d, *J* = 8.1 Hz, 1H), 7.50-7.07 (m, 13H), 6.61 (b.s, 1H), 5.40-5.38 (m, 2H), 4.53 (d, *J* = 5.4 Hz, 1H), 4.51 (d, *J* = 5.5 Hz, 1H), 2.57-2.49 (m, 1H), 2.49-2.36 (m, 2H), 2.36-2.23 (m, 1H).

^{13}C NMR (101 MHz, $CDCl_3$) δ 203.18, 174.7, 150.6, 149.5, 143.8, 139.84, 136.7, 135.06, 128.2 (2C), 128.7 (2C), 128.69 (2C), 128.63 (2C), 128.2 (2C), 127.8, 126.3, 124.8, 124.2, 115.2, 68.7, 48.9, 48.5, 32.0, 25.8.

IR: 3307, 3021, 2902, 1734, 1675, 1567, 1480, 1465, 1384, 1347, 1291, 1226, 1162, 1097, 804, 768, 670 cm^{-1}.

HRMS (ESI): m/z $[M+Na]^+$ calcd for $[C_{28}H_{23}NO_5Na]^+$: 476.1468; found: 476.1464.

(9*H*-Fluoren-9-yl)methyl (*R)-3-((*R**)-(2-hydroxy-3-oxocyclopent-1-en-1-yl)(phenyl)methyl)-2-oxoindoline-1-carboxylate (3c)**

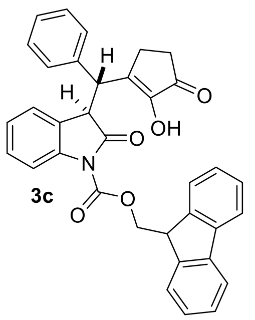

3c

Synthesized according to general procedure I, using cyclopentane-1,2-dione (**1**), (9*H*-fluoren-9-yl)methyl (*E*)-3-benzylidene-2-oxoindoline-1-carboxylate (**2c**) and catalyst **D**. Compound **3c** was obtained as an yellow solid in 52% yield (25.8 mg). <u>Major diastereoisomer</u>: ee 82% [HPLC (CHIRALPAK® AD-H, hexane/isopropanol 9:1, 25 °C, 1 mL/min, 210 nm): t_R (major) = 111.6 min, t_R (minor) = 173.7 min].

^{1}H NMR (400 MHz, $CDCl_3$) δ 7.81-7.75 (m, 2H), 7.73-7.66 (m, 2H), 7.56 (d, *J* = 8.2 Hz, 1H), 7.47-7.37 (m, 2H), 7.36-7.31 (m, 2H), 7.31-7.27 (m, 2H), 7.25-7.19 (m, 2H), 7.18-7.10 (m, 2H), 6.90 (td, *J* = 7.6, 1.1 Hz, 1H), 6.49 (d, *J* = 7.6 Hz, 1H), 5.88 (b.s, 1H), 4.70-4.62 (m, 3H), 4.40-4.34 (m, 1H), 4.3 (d, *J* = 9.0 Hz, 1H), 2.77-2.67 (m, 1H), 2.59-2.28 (m, 3H).

^{13}C NMR (101 MHz, $CDCl_3$) δ 203.07, 173.7, 150.8, 148.8, 143.86, 143.54, 143.4, 141.52 (2C), 141.49 (2C), 139.9, 137.7, 129.2 (2C), 128.90 (2C), 128.09 (2C), 128.08

(2C), 127.37, 126.1, 125.47, 125.4, 125.3, 124.3, 120.18, 115.1, 69.1, 49.98, 47.6, 46.7, 31.9, 24.7.

Minor diastereoisomer: ee 82% [HPLC (CHIRALPAK® AD-H, hexane/isopropanol 9:1, 25 °C, 1 mL/min, 210 nm): t_R (major) = 211.4 min, t_R (minor) = 104.3 min].

^{1}H NMR (400 MHz, $CDCl_3$) δ 7.81-7.75 (m, 2H), 7.73-7.66 (m, 2H), 7.55 (d, *J* = 8.1 Hz, 1H), 7.47-7.37 (m, 2H), 7.36-7.31 (m, 2H), 7.31-7.27 (m, 3H), 7.25-7.19 (m, 3H), 7.18-7.10 (m, 2H), 6.55 (b.s, 1H), 4.70-4.62 (m, 2H), 4.54 (s, 2H) 4.40-4.34 (m, 1H), 2.59-2-28 (m, 4H).

^{13}C NMR (101 MHz, $CDCl_3$) δ 203.11, 174.7, 150.7, 149.5, 143.87, 143.53, 143.3, 141.52 (2C), 141.49 (2C), 139.8, 136.8, 128.8 (2C), 128.7 (2C), 128.5, 127.9, 127.4 (2C), 127.40 (2C), 126.3, 125.5, 124.8, 124.2, 120.17, 115.2, 69.2, 49.0, 48.7, 46.7, 32.0, 25.9.

IR: 3322, 1770, 1727, 1696, 1654, 1605, 1480, 1466, 1451, 1386, 1346, 1292, 1246, 1161, 1095, 1053, 758, 740, 703, 641, 549 cm^{-1}.

HRMS (ESI): m/z $[M+Na]^+$ calcd for $[C_{35}H_{27}NO_5Na]^+$: 564.1781; found: 564.1778.

(*R)-3-((*R**)-(2-Hydroxy-3-oxocyclopent-1-en-1-yl)(phenyl)methyl-1-tosylindolin-2-one (3e)**

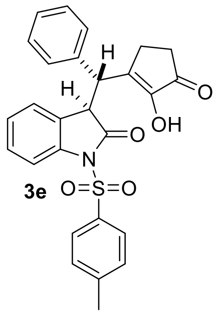

3e

Synthesized according to general procedure I, using cyclopentane-1,2-dione (**1**), (*E*)-3-benzylidene-1-tosylindolin-2-one (**2e**) and catalyst **D**. Compound **3e** was obtained as a yellow solid in 42% yield (20.1 mg, dr 2.1:1). The enantiomeric purity could not be determined.

Major diastereoisomer: ^{1}H NMR (400 MHz, $CDCl_3$) δ 7.88 (d, *J* = 8.4 Hz, 2H), 7.85 (d, *J* = 8.2 Hz, 1H), 7.32-7.26 (m, 3H), 7.24-7.16 (m, 3H), 7.11-7.05 (m, 2H), 6.92 (td, *J* = 7.6, 1.1 Hz, 1H), 6.50 (t, *J* = 7.6 Hz, 1H), 5.84 (b.s, 1H), 4.53 (d, *J* = 8.6 Hz, 1H), 4.11 (d, *J* = 8.6 Hz, 1H), 2.58-2.47 (m, 1H), 2.43 (s, 3H), 2.41-2.37 (m, 2H), 2.33-2.23 (m, 1H).

^{13}C NMR (101 MHz, $CDCl_3$) δ 203.2, 173.8, 148.8, 145.7, 143.7, 139.7, 137.5, 135.3, 129.9 (2C), 129.1 (2C), 129.0, 128.8 (2C), 128.0 (2C), 127.9, 126.2, 125.7, 124.4, 113.5, 48.8, 47.5, 31.9, 24.8, 21.9.

Minor diastereoisomer: ^{1}H NMR (400 MHz, $CDCl_3$) δ 7.92-7.86 (m, 2H), 7.84 (d, *J* = 8.3 Hz, 1H), 7.32-7.26 (m, 3H), 7.24-7.16 (m, 3H), 7.16-7.11 (m, 2H), 7.02-6.95 (m, 2H), 4.49 (d, *J* = 5.3 Hz, 1H), 4.40 (d, *J* = 5.3 Hz, 1H), 2.58-2.47 (m, 1H), 2.45 (m, 3H), 2.41-2.37 (m, 1H), 2.33-2.23 (m, 2H).

^{13}C NMR (101 MHz, $CDCl_3$) δ 203.3, 174.2, 149.2, 145.9, 143.6, 139.5, 136.5, 135.1, 128.9 (2C), 128.9, 128.7 (2C), 128.6 (2C), 128.1 (2C), 127.7, 126.4, 124.77, 124.75, 113.6, 48.6, 47.7, 32.0, 25.7, 21.2.

IR: 2926, 1755, 1706, 1601, 1493, 1462, 1379, 1235, 1177, 1087, 814, 755, 703, 663, 569, 543 cm^{-1}.

HRMS (ESI): m/z $[M+Na]^+$ calcd for $[C_{27}H_{23}NO_5SNa]^+$: 496.1189; found: 496.1187.

tert-Butyl (_R_*)-3-((_S_*)- (2-chlorophenyl)(2-hydroxy-3-oxocyclopent-1-en-1-yl)methyl)-2-oxoindoline-1-carboxylate (3f)

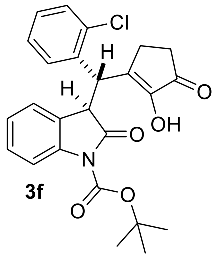

3f

Synthesized according to general procedure I, using cyclopentane-1,2-dione (**1**), *tert*-butyl (*E*)-3-(2-chlorobenzylidene)-2-oxoindoline-1-carboxylate (**2f**) and catalyst **D**.

Compound **3f** was obtained as an orange solid in 72% yield (33.5 mg, dr 1.7:1). Major diastereoisomer: ee 76% [HPLC (CHIRALPAK® AD-H, hexane/ethanol/isopropanol 95:3:2, 25 °C, 1 mL/min, 210 nm): t_R (major) = 32.6 min, t_R (minor) = 99.0 min].

^{1}H NMR (400 MHz, $CDCl_3$) δ 7.77 (d, *J* = 8.2 Hz, 1H), 7.63 (dd, *J* = 7.7, 1.8 Hz, 1H), 7.43-7.16 (m, 3H), 7.08-7.03 (m, 1H), 6.88 (td, *J* = 7.6, 1.1 Hz, 1H), 6.31 (d, *J* = 7.5 Hz, 1H), 5.86 (b.s, 1H), 4.76 (d, *J* = 9.5 Hz, 1H), 4.62 (d, *J* = 9.5 Hz, 1H), 2.61-2.52 (m, 1H), 2.51-2.37 (m, 3H), 1.63 (s, 9H).

^{13}C NMR (101 MHz, $CDCl_3$) δ 203.2, 173.7, 149.32, 149.25, 143.0, 140.23, 136.5, 135.1, 130.1, 130.0, 129.1, 128.62, 127.4, 125.89, 124.5, 124.1, 114.88, 84.62, 46.8, 44.3, 32.0, 28.23, 25.3.

Minor diastereoisomer: ee 68% [HPLC (CHIRALPAK® AD-H, hexane/ethanol/isopropanol 95:3:2, 25 °C, 1 mL/min, 210 nm): t_R (major) = 43.7 min, t_R (minor) = 25.0 min].

^{1}H NMR (400 MHz, $CDCl_3$) δ 7.74 (d, *J* = 8.3 Hz, 1H), 7.43-7.16 (m, 7H), 6.13 (b.s, 1H), 5.08 (d, *J* = 6.6 Hz, 1H), 4.56 (d, *J* = 6.6 Hz, 1H), 2.51-2.37 (m, 3H), 2.23-2.14 (m, 1H), 1.62 (s, 9H).

^{13}C NMR (101 MHz, $CDCl_3$) δ 203.1, 174.4, 149.36, 149.17, 143.2, 140.21, 135.5, 134.6, 129.95, 129.7, 128.95, 128.6, 127.1, 125.86, 124.3, 114.91, 84.6, 47.6, 43.9, 31.97, 28.2, 25.9.

IR: 3333, 2982, 2929, 1732, 1704, 1657, 1479, 1392, 1371, 1349, 1293, 1254, 1151, 1096, 842 cm^{-1}.

HRMS (ESI): m/z $[M+Na]^+$ calcd for $[C_{25}H_{24}ClNO_5Na]^+$: 476.1235; found: 476.1231.

tert-Butyl (R*)-3-((R*)-(3-chlorophenyl)(2-hydroxy-3-oxocyclopent-1-en-1-yl)methyl)-2-oxoindoline-1-carboxylate (3g)

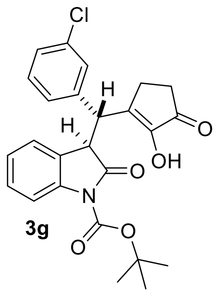

3g

Synthesized according to general procedure I, using cyclopentane-1,2-dione (**1**), *tert*-butyl (*E*)-3-(3-chlorobenzylidene)-2-oxoindoline-1-carboxylate (**2g**) and catalyst **D**. Compound **3g** was obtained as an yellow solid in 66% yield (33.3 mg, dr 2:1). <u>Major diastereoisomer:</u> ee 87% [HPLC (CHIRALPAK® AD-H, hexane/ethanol/isopropanol 95:3:2, 25 °C, 1 mL/min, 210 nm): t_R (major) = 26.6 min, t_R (minor) = 111.4 min].

^{1}H NMR (400 MHz, $CDCl_3$) δ 7.72 (d, *J* = 8.3 Hz, 1H), 7.30-7.06 (m, 5H), 6.93 (td, *J* = 7.6, 1.1 Hz, 1H), 6.52 (d, *J* = 7.6 Hz, 1H), 5.88 (b.s, 1H), 4.57 (d, *J* = 8.9 Hz, 1H), 4.21 (d, *J* = 8.9 Hz, 1H), 2.72-2.61 (m, 1H), 2.55-2.40 (m, 2H), 2.40-2.30 (m, 1H), 1.61 (s, 9H).

^{13}C NMR (101 MHz, $CDCl_3$) δ 202.9, 173.6, 148.9, 148.8, 142.9, 140.3, 139.8, 134.6, 129.9, 129.1, 128.65, 128.0, 127.3, 125.5, 125.1, 123.95, 114.8, 84.6, 48.5, 47.2, 31.8, 28.1, 24.6.

<u>Minor diastereoisomer</u>: ee 90% [HPLC (CHIRALPAK® AD-H, hexane/ethanol/isopropanol 95:3:2, 25 °C, 1 mL/min, 210 nm): t_R (major) = 45.2 min, t_R (minor) = 42.1 min].

^{1}H NMR (400 MHz, $CDCl_3$) δ 7.69 (d, *J* = 8.3 Hz, 1H), 7.30-7.06 (m, 6H), 6.97 (dt, *J* = 7.6, 1.5 Hz, 1H), 6.77 (b.s, 1H), 4.44 (d, *J* = 5.2 Hz, 1H), 4.42 (d, *J* = 5.2 Hz, 1H), 2.44-2.40 (m, 4H), 1.59 (s, 9H).

^{13}C NMR (101 MHz, $CDCl_3$) δ 202.96, 174.8, 149.5, 148.7, 142.4, 140.2, 138.7, 134.4, 129.7, 128.82, 128.77, 127.9, 126.9, 125.8, 124.5, 123.97, 115.0, 84.7, 48.6, 48.4, 31.9, 28.0, 25.7.

IR: 3339, 2982, 2924, 1835, 1735, 1620, 1477, 1370, 1347, 1290, 1254, 1150, 1097, 771, 696 cm^{-1}.

HRMS (ESI): m/z $[M+Na]^+$ calcd for $[C_{25}H_{24}ClNO_5Na]^+$: 476.1235; found: 476.1232.

***tert*-Butyl (*R**)-3-((*R**)-(4-chlorophenyl)(2-hydroxy-3-oxocyclopent-1-en-1-yl)methyl)-2-oxoindoline-1-carboxylate (3h)**

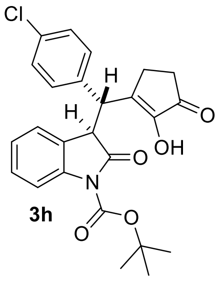

Synthesized according to general procedure I, using cyclopentane-1,2-dione (**1**), *tert*-butyl (*E*)-3-(4-chlorobenzylidene)-2-oxoindoline-1-carboxylate (**2h**) and catalyst **D**. Compound **3h** was obtained as an orange solid in 87% yield (36.1 mg, 2.7:1). <u>Major diastereoisomer</u>: ee 84% [HPLC (CHIRALPAK® AD-H, hexane/ethanol/isopropanol 95:3:2, 25 °C, 1 mL/min, 210 nm): t_R (major) = 30.0 min, t_R (minor) = 80.9 min].

^1H NMR (400 MHz, $CDCl_3$) δ 7.71 (d, *J* = 8.5 Hz, 1H), 7.27-7.24 (m, 2H), 7.16-7.10 (m, 2H), 6.94 (td, *J* = 7.61, 1.08 Hz, 1H), 6.56 (d, *J* = 7.5 Hz, 1H), 5.86 (b.s, 1H), 4.58 (d, *J* = 8.6 Hz, 1H), 4.24 (d, *J* = 8.6 Hz, 1H), 2.69-2.58 (m, 1H), 2.57-2.29 (m, 3H), 1.6 (s, 9H).

^{13}C NMR (101 MHz, $CDCl_3$) δ 203.1, 173.9, 149.0 148.9, 147.9, 143.5, 140.4, 136.4, 133.8, 130.6, 130.2, 129.0, 125.3, 124.1, 114.96, 84.7, 48.3, 47.5, 31.9, 28.2, 24.8.

Minor diastereoisomer: ee 81% [HPLC (CHIRALPAK® AD-H, hexane/ethanol/isopropanol 95:3:2, 25 °C, 1 mL/min, 210 nm): t_R (major) = 23.6 min, t_R (minor) = 35.6 min].

^{1}H NMR (400 MHz, $CDCl_3$) δ 7.66 (d, *J* = 8.2 Hz, 1H), 7.18-7.16 (m, 2H), 7.16-7.10 (m, 1H), 7.04-6.99 (m, 2H), 6.84 (b.s, 1H), 4.44 (d, *J* = 4.9 Hz, 1H), 4.41 (d, *J* = 5.0 Hz, 1H), 2.57-2.29 (m, 4H), 1.58 (s, 9H).

^{13}C NMR (101 MHz, $CDCl_3$) δ 203.2, 175.1, 149.6, 147.2, 145.7, 143.0, 140.3, 135.2, 133.7, 128.93, 128.78, 128.74, 125.7, 124.7, 115.1, 84.9, 48.8, 48.4, 32.1, 28.2, 25.9.

IR: 3306, 2981, 2956, 1784, 1732, 1709, 1620, 1492, 1468, 1393, 1371, 1347, 1291, 1254, 1150, 1094, 1015, 839, 755, 669 cm^{-1}.

HRMS (ESI): m/z $[M+H]^+$ calcd for $[C_{25}H_{25}ClNO_5]^+$: 454.1416; found: 454.1617.

tert-Butyl (_R_*)-3-((_S_*)-2-ethoxy-1-(2-hydroxy-3-oxocyclopent-1-en-1-yl)-2-oxoethyl)-2-oxoindoline-1-carboxylate (3i)

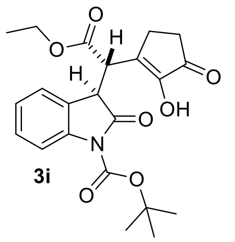

3i

Synthesized according to general procedure I, using cyclopentane-1,2-dione (**1**), *tert*-butyl (*E*)-3-(2-ethoxy-2-oxoethylidene)-2-oxoindoline-1-carboxylate (**2i**) and catalyst **D**. Compound **3i** was obtained as an yellow oil in 87% yield (36.5 mg, dr 1.2:1). Major diastereoisomer: ee 5% [HPLC (CHIRALPAK® AD-H, hexane/isopropanol 93:7, 25 °C, 1 mL/min, 210 nm): t_R (major) = 33.0 min, t_R (minor) = 17.3 min].

^{1}H NMR (400 MHz, $CDCl_3$) δ 7.83-7.76 (m, 1H), 7.34-7.27 (m, 2H), 7.15-7.08 (m, 1H), 6.06 (m, 1H), 4.46-4.39 (m, 1H), 4.26-4.11 (m, 3H), 2.80-2.25 (m, 4H), 1.63 (s, 9H), 1.23-1.14 (m, 3H).

^{13}C NMR (101 MHz, $CDCl_3$) δ 202.8, 174.2, 169.8, 150.4, 149.10, 140.5, 138.7, 128.91, 124.6, 124.5, 124.1, 115.1, 84.7, 61.99, 46.8, 46.4, 32.2, 28.2 (3C), 24.7, 14.1.

Minor diastereoisomer: *racemic* [HPLC (CHIRALPAK® AD-H, hexane/isopropanol 93:7, 25 °C, 1 mL/min, 210 nm):): t_{R1} = 22.9 min, t_{R2} = 28.6 min].

^{1}H NMR (400 MHz, $CDCl_3$) δ 7.83-7.76 (m, 1H), 7.24-7.15 (m, 2H), 6.91-6.85 (m, 1H), 6.06 (m, 1H), 4.38-4.30 (m, 2H), 4.26-4.11 (m, 2H), 2.80-2.25 (m, 4H), 1.63 (s, 9H), 1.23-1.14 (m, 3H).

^{13}C NMR (101 MHz, $CDCl_3$) δ 203.0, 173.7, 169.5, 150.7, 149.09, 140.3, 137.8, 128.94, 125.3, 125.2, 124.1, 115.1, 84.8, 61.98, 46.5, 45.9, 32.1, 28.2 (3C), 24.2, 14.0.

IR: 3335, 2981, 2920, 1805, 1732, 1664, 1608, 1481, 1466, 1370, 1351, 1297, 1252, 1152, 1097, 1028, 845, 745 cm^{-1}.

HRMS (ESI): m/z $[M+Na]^+$ calcd for $[C_{22}H_{25}NO_7Na]^+$: 438.1523; found: 438.1518.

***tert*-Butyl (*R**)-3-((*S**)- (2-hydroxy-3-oxocyclopent-1-en-1-yl)(thiophen-2-yl)methyl)-2-oxoindoline-1-carboxylate (3j)**

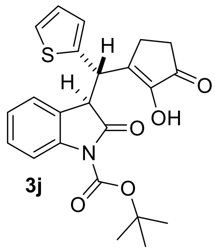

3j

Synthesized according to general procedure I, using cyclopentane-1,2-dione (**1**), *tert*-butyl (*E*)-2-oxo-3-(thiophen-2-ylmethylene)indoline-1-carboxylate (**2j**) and catalyst **D**. Compound **3j** was obtained as an yellow solid in 39% yield (16.8 mg, dr 2.1:1). Major diastereoisomer: ee 75% [HPLC (CHIRALPAK® AD-H, hexane/isopropanol 95:5, 25 °C, 1 mL/min, 210 nm): t_R (major) = 63.2 min, t_R (minor) = 46.0 min].

^{1}H NMR (400 MHz, $CDCl_3$) δ 7.75 (d, *J* = 8.2 Hz, 1H), 7.32-7.26 (m, 1H), 7.18 (dd, *J* = 5.2, 1.2 Hz, 1H), 7.00 (td, *J* = 7.6, 1.1 Hz, 1H), 6.92 (dd, *J* = 5.1, 3.5 Hz, 1H), 6.84 (dd,

J = 3.5, 1.2 Hz, 1H), 6.67 (d, J = 7.6 Hz, 1H), 5.86 (b.s, 1H), 4.73 (d, J = 8.0 Hz, 1H), 4.49 (d, J = 7.9 Hz, 1H), 2.75-2.64 (m, 1H), 2.51-2.38 (m, 3H), 1.60 (s, 9H).

^{13}C NMR (101 MHz, $CDCl_3$) δ 203.0, 173.4, 149.1, 148.6, 143.4, 140.7, 139.6, 128.89, 127.5, 126.8, 125.6, 125.4, 125.1, 124.2, 115.0, 84.6, 48.6, 43.4, 31.9, 28.2 (3C), 24.7.

Minor diastereoisomer: ee 85% [HPLC (CHIRALPAK® AD-H, hexane/isopropanol 95:5, 25 °C, 1 mL/min, 210 nm): t_R (major) = 28.6 min, t_R (minor) = 58.2 min].

^{1}H NMR (400 MHz, $CDCl_3$) δ 7.70 (d, J = 8.2 Hz, 1H), 7.32-7.26 (m, 1H), 7.21 (d, J = 7.4 Hz, 1H), 7.15-7.09 (m, 2H), 6.88 (dd, J = 5.1, 3.6 Hz, 1H), 6.82 (dt, J = 3.6, 1.0 Hz, 1H), 6.49 (b.s, 1H), 4.98 (d, J = 4.1 Hz, 1H), 4.37 (d, J = 4.1 Hz, 1H), 2.63-2.53 (m, 1H), 2.51-5.38 (m, 2H), 2.38-2.31 (m, 1H), 1.61 (s, 9H).

^{13}C NMR (101 MHz, $CDCl_3$) δ 203.1, 174.7, 149.3, 149.0, 143.3, 140.5, 138.9, 128.87, 127.0, 126.5, 125.8, 125.3, 124.9, 124.5, 115.1, 84.7, 50.2, 42.5, 32.2, 28.2 (3C), 25.5.

IR: 3263, 2980, 2927, 1770, 1723, 1700, 1661, 1500, 1480, 1413, 1391, 1370, 1350, 1292, 1256, 1151, 1095, 838, 755, 697 cm^{-1}.

HRMS (ESI): m/z $[M+Na]^+$ calcd for $[C_{23}H_{23}NO_5SNa]^+$: 448.1189; found: 448.1184.

tert-Butyl (_R*_)-5-bromo-3-((_R*_)-(2-hydroxy-3-oxocyclopent-1-en-1-yl)(phenyl)methyl)-2-oxoindoline-1-carboxylate (3k)

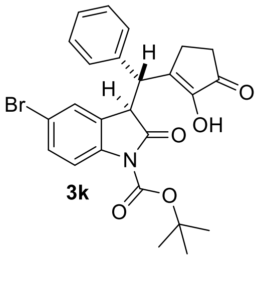

Synthesized according to general procedure I, using cyclopentane-1,2-dione (**1**), _tert_-butyl (_E_)-3-benzylidene-5-bromo-2-oxoindoline-1-carboxylate (**2k**) and catalyst **D**. Compound **3k** was obtained as an yellow solid in 70 yield (35.5 mg, dr 3.2:1). Major diastereoisomer: ee 76% [HPLC (CHIRALPAK® AD-H, hexane/ethanol/isopropanol 95:3:2, 25 °C, 1 mL/min, 210 nm): t_R (major) = 45.5 min, t_R (minor) = 73.3 min].

^{1}H NMR (400 MHz, $CDCl_3$) δ 7.60 (d, *J* = 8.7 Hz, 1H), 7.37-7.33 (m, 1H), 7.34-7.29 (m, 2H), 7.25-7.21 (m, 1H), 7.21-7.16 (m, 2H), 6.49-6.46 (m, 1H), 5.8 (b.s, 1H), 4.59 (d, *J* = 8.9 Hz, 1H), 4.16 (d, *J* = 8.9 Hz, 1H), 2.70-2.59 (m, 1H), 2.49-2.38 (m, 3H), 1.59 (s, 9H).

^{13}C NMR (101 MHz, $CDCl_3$) δ 203.1, 173.2, 148.9, 148.7, 143.4, 139.39, 137.6, 131.4, 129.2 (2C), 129.0 (2C), 128.6, 128.2, 128.1, 116.9, 116.3, 84.9, 49.07, 47.6, 31.8, 28.19 (3C), 24.9.

<u>Minor diastereoisomer</u>: ee 76% [HPLC (CHIRALPAK® AD-H, hexane/ethanol/isopropanol 95:3:2, 25 °C, 1 mL/min, 210 nm): t_R (major) = 94.2 min, t_R (minor) = 61.3 min].

^{1}H NMR (400 MHz, $CDCl_3$) δ 7.56 (d, *J* = 8.7 Hz, 1H), 7.40-7.37 (m, 1H), multiplet, 7.29-7.27 (m, 1H), 7.11-7.07 (m, 2H), 6.59 (b.s, 1H), 4.47 (d, *J* = 4.8 Hz, 1H), 4.44 (d, *J* = 4.8 Hz, 1H), 2.57-2.49 (m, 1H), 2.49-2.38 (m, 2H), 2.38-2.28 (m, 1H), 1.57 (s, 9H).

^{13}C NMR (101 MHz, $CDCl_3$) δ 203.0, 174.3, 149.5, 149.0, 143.0, 139.37, 136.3, 131.6, 129.1, 128.78 (2C), 128.76 (2C), 128.0, 127.3, 117.4, 116.6, 85.0, 48.99, 48.7, 32.0, 28.15 (3C), 26.0.

IR: 3356, 2980, 2930, 1769, 1758, 1702, 1657, 1471, 1391, 1371, 1337, 1296, 1255, 1152, 1067, 821, 735, 702, 636 cm^{-1}.

HRMS (ESI): m/z $[M+Na]^+$ calcd for $[C_{25}H_{24}NO_5BrNa]^+$: 520.0730; found: 520.0722.

tert-Butyl (_R_*)-4-bromo-3-((_R_*)-(2-hydroxy-3-oxocyclopent-1-en-1-yl)(phenyl)methyl)-2-oxoindoline-1-carboxylate (3l)

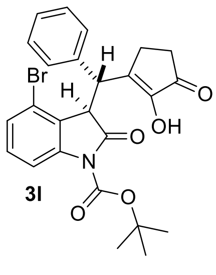

3l

Synthesized according to general procedure I, using cyclopentane-1,2-dione (**1**), *tert*-butyl (*E*)-3-benzylidene-4-bromo-2-oxoindoline-1-carboxylate (**2l**) and catalyst **D**. Compound **3l** was obtained as an yellow solid in 21% yield (11.1 mg, dr 4.7:1). Major diastereoisomer: ee 62% [HPLC (CHIRALPAK® AD-H, hexane/isopropanol 97:3, 25 °C, 1 mL/min, 210 nm): t_R (major) = 46.9 min, t_R (minor) = 60.8 min].

^{1}H NMR (400 MHz, $CDCl_3$) δ 7.55 (s, 1H), 7.52 (d, *J* = 8.1 Hz, 1H), 7.42-7.29 (m, 2H), 7.20-7.05 (m, 3H), 6.92-6.86 (m, 2H), 4.91 (d, *J* = 2.9 Hz, 1H), 4.50 (d, *J* = 2.9 Hz, 1H), 2.63-2.57 (m, 1H), 2.54-2.50 (m, 1H), 2.49-2.44 (m, 2H), 1.52 (s, 9H).

^{13}C NMR (101 MHz, $CDCl_3$) δ 203.5, 175.1, 149.8, 148.1, 142.7, 141.7, 135.1, 130.4, 128.9 (2C), 128.7, 128.29 (2C), 125.98, 118.9, 113.9, 85.5, 50.7, 47.1, 31.92, 28.07 (3C), 26.5.

Minor diastereoisomer: ee 60% [HPLC (CHIRALPAK® AD-H, hexane/isopropanol 97:3, 25 °C, 1 mL/min, 210 nm): t_R (major) = 36.8 min, t_R (minor) = 31.5 min].

^{1}H NMR (400 MHz, $CDCl_3$) δ 7.66 (d, *J* = 8.1 Hz, 1H), 7.61-7.56 (m, 2H), 7.42-7.29 (m, 1H), 7.20-7.05 (m, 4H), 5.22 (d, *J* = 2.4 Hz, 1H), 5.07 (b.s, 1H), 4.32 (d, *J* = 2.5 Hz, 1H), 2.67-2.62 (m, 2H), 2.29-2.23 (m, 2H), 1.58 (s, 9H).

^{13}C NMR (101 MHz, $CDCl_3$) δ 203.2, 176.4, 151.5, 148.0, 142.5, 141.0, 133.8, 130.2, 129.3, 128.26 (2C), 128.0 (2C), 125.87, 119.5, 113.6, 84.9, 51.5, 45.6, 32.07, 28.17 (3C), 24.8.

IR: 3325, 2981, 2919, 1830, 1737, 1682, 1651, 1601, 1447, 1391, 1371, 1343, 1289, 1256, 1155, 1118, 844, 759, 703, 626 cm^{-1}.

HRMS (ESI): m/z $[M+Na]^+$ calcd for $[C_{25}H_{24}NO_5BrNa]^+$: 520.0730; found: 520.0721.

tert-Butyl (_R*_)-3-((_R*_)-(2-hydroxy-3-oxocyclopent-1-en-1-yl)(4-methoxyphenyl)methyl)-2-oxoindoline-1-carboxylate (3m)

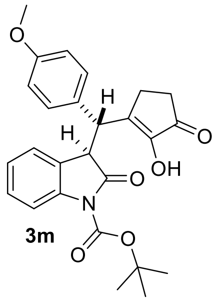

3m

Synthesized according to general procedure I, using cyclopentane-1,2-dione **1**, _tert_-butyl (_E_)-3-(4-methoxybenzylidene)-2-oxoindoline-1-carboxylate **2m** and catalyst **D**. Compound **3m** was obtained as an yellow amorphous solid in 36% yield (16.5 mg, dr 3.6:1). The enantiomeric purity could not be determined.

Major diastereoisomer: ^{1}H NMR (400 MHz, $CDCl_3$) δ 7.71 (d, _J_ = 8.1 Hz, 1H), 7.28-7.18 (m, 1H), 7.16-7.08 (m, 2H), 6.90 (td, _J_ = 7.6, 1.1 Hz, 1H), 6.85-6.79 (m, 2H), 6.47 (d, _J_ = 7.7 Hz, 1H), 5.86 (b.s, 1H), 4.55 (d, _J_ = 9.2 Hz, 1H), 4.16 (d, _J_ = 9.3 Hz, 1H), 3.8 (s, 3H), 2.74-2.62 (m, 1H), 2.55-2.32 (m, 3H), 1.60 (s, 9H).

^{13}C NMR (101 MHz, $CDCl_3$) δ 203.1, 173.9, 158.9, 149.0, 148.4, 144.8, 140.22, 130.1 (2C), 129.9, 128.4, 126.1, 125.3, 123.8, 114.7, 114.1 (2C), 84.4, 55.3, 48.0, 47.5, 31.8, 28.1 (3C), 24.5.

Minor diastereoisomer: ^{1}H NMR (400 MHz, $CDCl_3$) δ 7.65 (d, _J_ = 8.1Hz, 1H), 7.28-7.18 (m, 1H), 7.16-7.08 (m, 2H), 7.02-6.94 (m, 2H), 6.85-6.79 (m, 1H), 6.74-6.70 (m, 2H), 4.41 (s, 2H), 3.73 (s, 3H), 2.55-2.32 (m, 4H), 1.58 (s, 9H).

^{13}C NMR (101 MHz, $CDCl_3$) δ 203.2, 175.5, 159.1, 149.2, 148.7, 144.1, 140.16, 130.7, 129.7 (2C), 128.6, 126.4, 124.4, 124.0, 114.9, 113.8 (2C), 84.5, 55.1, 49.2, 48.2, 31.9, 28.0 (3C), 25.9.

IR: 2980, 2933, 1738, 1761, 1610, 1514, 1466, 1370, 1347, 1288, 1253, 1149, 1098, 1033, 837, 770 cm^{-1}.

HRMS (ESI): m/z $[M+Na]^+$ calcd for $[C_{26}H_{27}NO_6Na]^+$: 472.1731; found: 472.1727.

tert-Butyl (_R_*)-3-((_R_*)-(2-hydroxy-3-oxocyclopent-1-en-1-yl)(_p_-tolyl)methyl)-2-oxoindoline-1-carboxylate (3n)

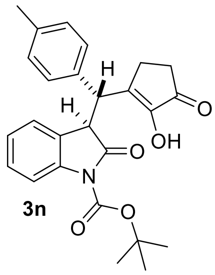

3n

Synthesized according to general procedure I, using cyclopentane-1,2-dione (**1**), *tert*-butyl (*E*)-3-(4-methylbenzylidene)-2-oxoindoline-1-carboxylate (**2n**) and catalyst **D**. Compound **3n** was obtained as an yellow solid in 59% yield (26.2 mg, dr 2.8:1). <u>Major diastereoisomer</u>: ee 80% [HPLC (CHIRALPAK® AD-H, hexane/ethanol/isopropanol 95:3:2, 25 °C, 1 mL/min, 210 nm): t_R (major) = 32.4 min, t_R (minor) = 76.1 min].

^{1}H NMR (400 MHz, $CDCl_3$) δ 7.71 (d, *J* = 8.2 Hz, 1H), 7.25-7.16 (m, 1H), 7.15-7.07 (m, 4H), 6.88 (td, *J* = 7.6 Hz, 1H), 6.44 (d, *J* = 7.6 Hz, 1H), 5.81 (b.s, 1H), 4.55 (d, *J* = 9.4 Hz, 1H), 4.17 (d, *J* = 9.5 Hz, 1H), 2.77-2.64 (m, 1H), 2.49-2.35 (m, 3H), 2.34 (s, 3H), 1.61 (s, 9H).

^{13}C NMR (101 MHz, $CDCl_3$) δ 203.2, 174.0, 149.2, 148.6, 144.7, 140.32, 137.6, 135.0, 129.6 (2C), 129.0 (2C), 128.5, 126.2, 125.4, 123.9, 114.8, 84.5, 48.45, 47.4, 31.9, 28.22 (3C), 24.5, 21.25.

<u>Minor diastereoisomer</u>: ee 79% [HPLC (CHIRALPAK® AD-H, hexane/ethanol/isopropanol 95:3:2, 25 °C, 1 mL/min, 210 nm): t_R (major) = 95.0 min, t_R (minor) = 39.1 min].

^{1}H NMR (400 MHz, $CDCl_3$) δ 7.66 (d, *J* = 8.2 Hz, 1H), 7.25-7.16 (m, 2H), 7.15-7.07 (m, 1H), 7.03-6.94 (m, 5H), 4.45 (d, *J* = 4.9 Hz, 1H), 4.43 (d, *J* = 4.9 Hz, 1H), 2.55-2.46 (m, 1H), 2.49-2.35 (m, 2H), 2.31-2.28 (m, 1H), 2.26 (s, 3H), 1.57 (s, 9H).

^{13}C NMR (101 MHz, $CDCl_3$) δ 203.4, 175.2, 149.5, 148.9, 144.1, 140.31, 137.4, 133.5, 129.3 (2C), 128.7, 128.6 (2C), 126.5, 124.5, 124.2, 115.0, 84.6, 49.1, 48.53, 32.0, 28.15 (3C), 25.9, 21.16.

IR: 3285, 2980, 2921, 1771, 1724, 1701, 1659, 1513, 1479, 1465, 1392, 1371, 1350, 1292, 1254, 1150, 1093, 842, 756, 624 cm^{-1}.

HRMS (ESI): m/z $[M+Na]^+$ calcd for $[C_{26}H_{27}NO_5Na]^+$: 456.1781; found: 456.1776.

***tert*-Butyl 3-(1-(2-hydroxy-3-oxocyclopent-1-en-1-yl)pentyl)-2-oxoindoline-1-carboxylate (3o)**

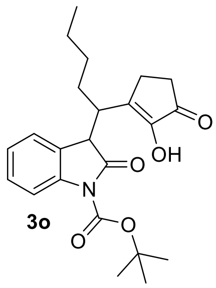

Synthesized according to general procedure I using cyclopentane-1,2-dione (**1**), *tert*-butyl (*E*)-2-oxo-3-pentylideneindoline-1-carboxylate (**2o**) and catalyst **D**. Compound **3o** was obtained as an yellow oil in 64% yield (26.2 mg, dr 1:1). <u>Diastereomer 1</u>: ee 74% [HPLC (CHIRALPAK® AD-H, hexane/isopropanol 98:2, 25 °C, 1 mL/min, 210 nm): t_{R1} = 31.9 min, t_{R2} = 44.7 min].

^1H NMR (400 MHz, $CDCl_3$) δ 7.79-7.72 (m, 1H), 7.35-7.22 (m, 2H), 7.19-7.07 (m, 1H), 5.65 (b.s, 1H), 3.83 (d, *J* = 4.8 Hz, 1H), 3.53-3.42 (m, 1H), 2.56-2.22 (m, 4H), 1.63 (s, 9H), 1.34-1.17 (m, 6H), 0.89-0.79 (m, 3H).

^{13}C NMR (101 MHz, $CDCl_3$) δ 203.5, 175.0, 149.9, 149.3, 145.5, 140.0, 128.7, 126.0, 124.5, 124.3, 114.8, 84.6, 49.7, 41.7, 32.0, 30.30, 28.25 (3C), 28.1, 23.9, 22.5, 14.05.

<u>Diastereomer 2</u>: ee 74% [HPLC (CHIRALPAK® AD-H, hexane/isopropanol 98:2, 25 °C, 1 mL/min, 210 nm): t_{R1} = 65.4 min, t_{R2} = 74.4 min].

^{1}H NMR (400 MHz, $CDCl_3$) δ 7.79-7.72 (m, 1H), 7.35-7.22 (m, 2H), 7.19-7.07 (m, 1H), 5.97 (b.s, 1H), 3.79 (d, *J* = 3.9 Hz, 1H), 3.53-3.42 (m, 1H), 2.56-2.22 (m, 4H), 1.63 (s, 9H), 1.34-1.17 (m, 6H), 0.89-0.79 (m, 3H).

^{13}C NMR (101 MHz, $CDCl_3$) δ 203.3, 174.9, 150.0, 149.2, 146.9, 140.5, 128.5, 126.6, 124.5, 124.3, 115.0, 84.7, 48.5, 41.1, 32.2, 30.27, 29.0, 28.2 (3C), 24.0, 22.7, 14.13.

IR: 3332, 2991, 2930, 1804, 1731, 1572, 1510, 1466, 1369, 1347, 1292, 1251, 1152, 1096, 854, 754, 668 cm^{-1}.

HRMS (ESI): m/z $[M+Na]^+$ calcd for $[C_{23}H_{29}NO_5Na]^+$: 422.1938; found: 422.1935.

tert-Butyl (_R*_)-3-((R*)-(2-hydroxy-3-oxocyclopent-1-en-1-yl)(3-nitrophenyl)methyl)-2-oxoindoline-1-carboxylate (3q)

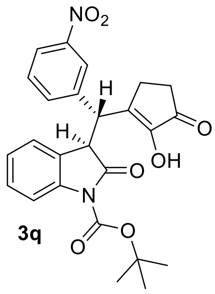

3q

Synthesized according to general procedure I, using cyclopentane-1,2-dione (**1**), *tert*-butyl (*E*)-3-(3-nitrobenzylidene)-2-oxoindoline-1-carboxylate (**2q**) or *tert*-butyl (*Z*)-3-(3-nitrobenzylidene)-2-oxoindoline-1-carboxylate **2q'** and catalyst **D**. Compound **3q** from **2q** was obtained as an yellow solid in 57% yield (27 mg, dr 1.4:1). Major diastereoisomer: ee 91% [HPLC (CHIRALPAK® AD-H, hexane/ethanol 95:5, 25 °C, 1 mL/min, 210 nm): t_R (major) = 204.8 min, t_R (minor) = 60.1 min].

^{1}H NMR (400 MHz, $CDCl_3$) δ 8.13 (ddd, *J* = 8.2, 2.3, 1.1 Hz, 1H), 8.03-7.99 (m, 1H), 7.69 (d, *J* = 8.2 Hz, 1H), 7.57-7.50 (m, 1H), 7.49-7.40 (m, 1H), 7.31-7.23 (m, 1H), 6.97 (td, *J* = 7.6, 1.1 Hz, 1H), 6.65 (d, *J* = 7.6 Hz, 1H), 5.98 (b.s, 1H), 4.66 (d, *J* = 7.8 Hz, 1H), 4.50-4.43 (m, 1H), 2.65-2.34 (m, 4H), 1.59 (s, 9H).

^{13}C NMR (101 MHz, $CDCl_3$) δ 202.82, 174.1, 149.4, 148.9, 148.29, 141.5, 140.4, 139.8, 135.5, 129.7, 129.1, 125.08, 125.06, 124.3, 124.0, 123.0, 115.1, 84.9, 48.5, 47.6, 31.8, 28.2 (3C), 25.0.

<u>Minor diastereoisomer</u>: ee 93% [HPLC (CHIRALPAK® AD-H, hexane/ethanol 95:5, 25 °C, 1 mL/min, 210 nm): t_R (major) = 134.3 min, t_R (minor) = 78.3 min].

^1H NMR (400 MHz, $CDCl_3$) δ 8.09 (ddd, *J* = 8.1, 2.3, 1.1 Hz, 1H), 8.03-7.99 (m, 1H), 7.69 (d, *J* = 8.3 Hz, 1H), 7.57-7.50 (m, 1H), 7.49-7.40 (m, 1H), 7.31-7.23 (m, 1H), 7.19 (d, *J* = 7.3 Hz, 1H), 7.13 (td, *J* = 7.5, 1.1 Hz, 1H), 6.48 (b.s, 1H), 4.52 (d, *J* = 6.3 Hz, 1H), 4.50-4.43 (m, 1H), 2.65-2.34 (m, 4H), 1.58 (s, 9H).

^{13}C NMR (101 MHz, $CDCl_3$) δ 202.79, 173.7, 149.7, 148.8, 148.26, 141.6, 140.2, 139.2, 135.0, 129.6, 129.2, 125.4, 124.8, 124.1, 123.8, 122.8, 115.2, 85.0, 48.6, 48.1, 32.0, 28.1 (3C), 25.6.

IR: 3386, 2981, 2924, 1789, 1765, 1708, 1657, 1531, 1480, 1466, 1392, 1350, 1293, 1254, 1150, 1096, 841, 756 cm^{-1}.

HRMS (ESI): m/z $[M+Na]^+$ calcd for $[C_{25}H_{24}N_2O_7Na]^+$: 487.1476; found: 487.1468.

Compound **3q** from **2q'** was obtained as an yellow solid in 53% yield (25 mg, dr 1.6:1). Major diastereoisomer: *ee* -77% [HPLC (CHIRALPAK® AD-H, hexane/ethanol 95:5, 25 °C, 1 mL/min, 210 nm): t_R (major) = 58.8 min, t_R (minor) = 213.3 min]. Minor diastereoisomer: ee −76% [HPLC (CHIRALPAK® AD-H, hexane/ethanol 95:5, 25 °C, 1 mL/min, 210 nm): t_R (major) = 76.6 min, t_R (minor) = 136.3 min].

2. ^{1}H NMR and ^{13}C NMR spectra

Catalyst **A** ^{1}H NMR

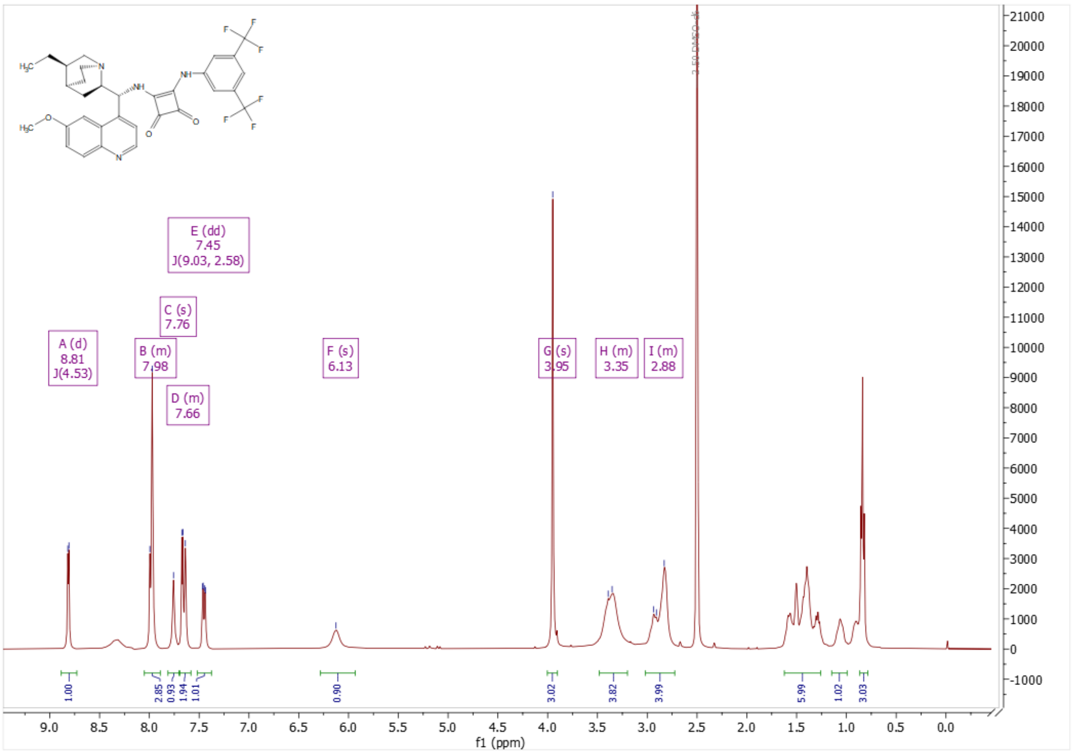

Catalyst **B** ^{1}H NMR

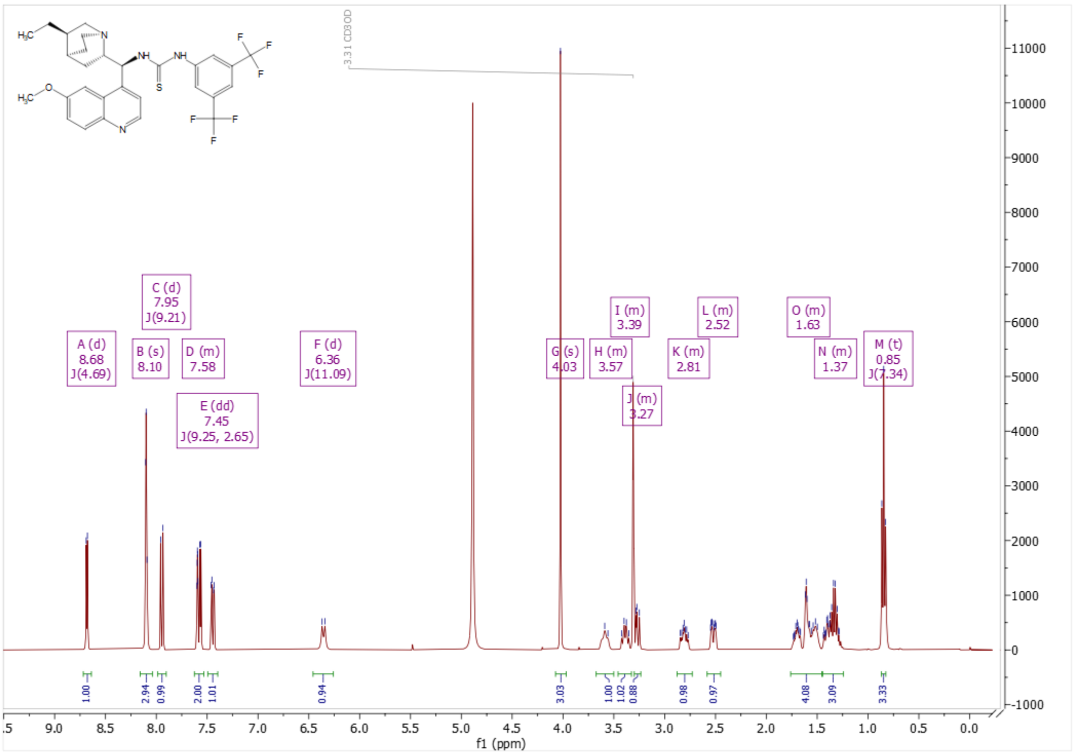

Catalyst **C** ^{1}H NMR

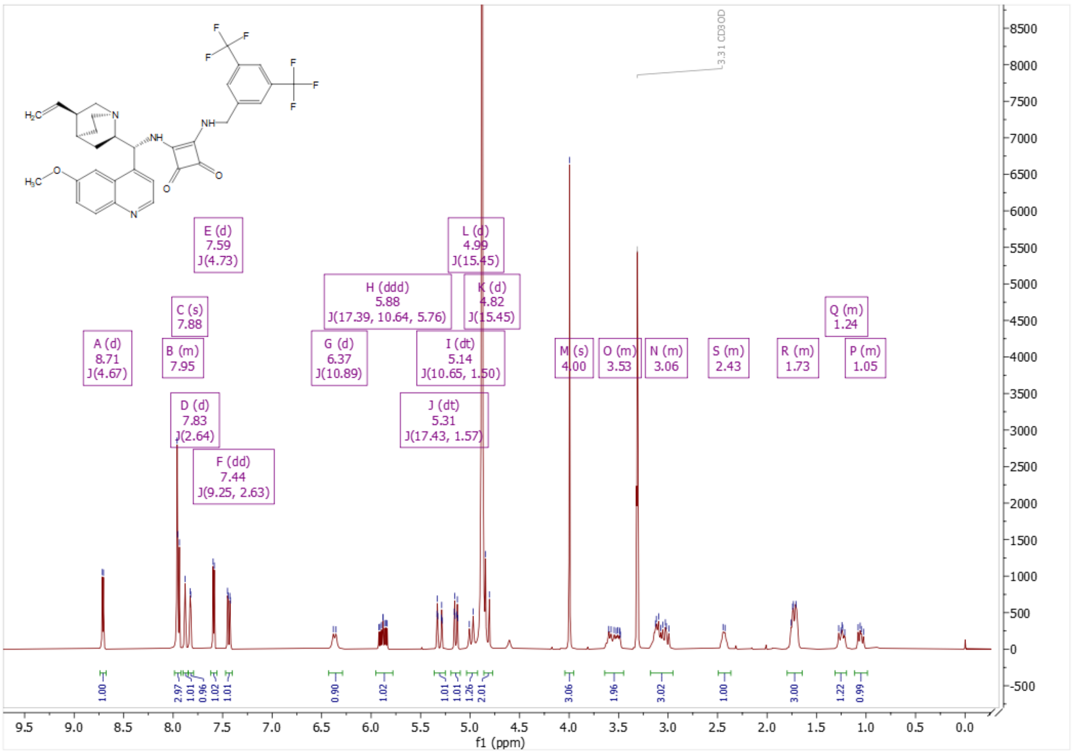

Catalyst **D** ^{1}H NMR

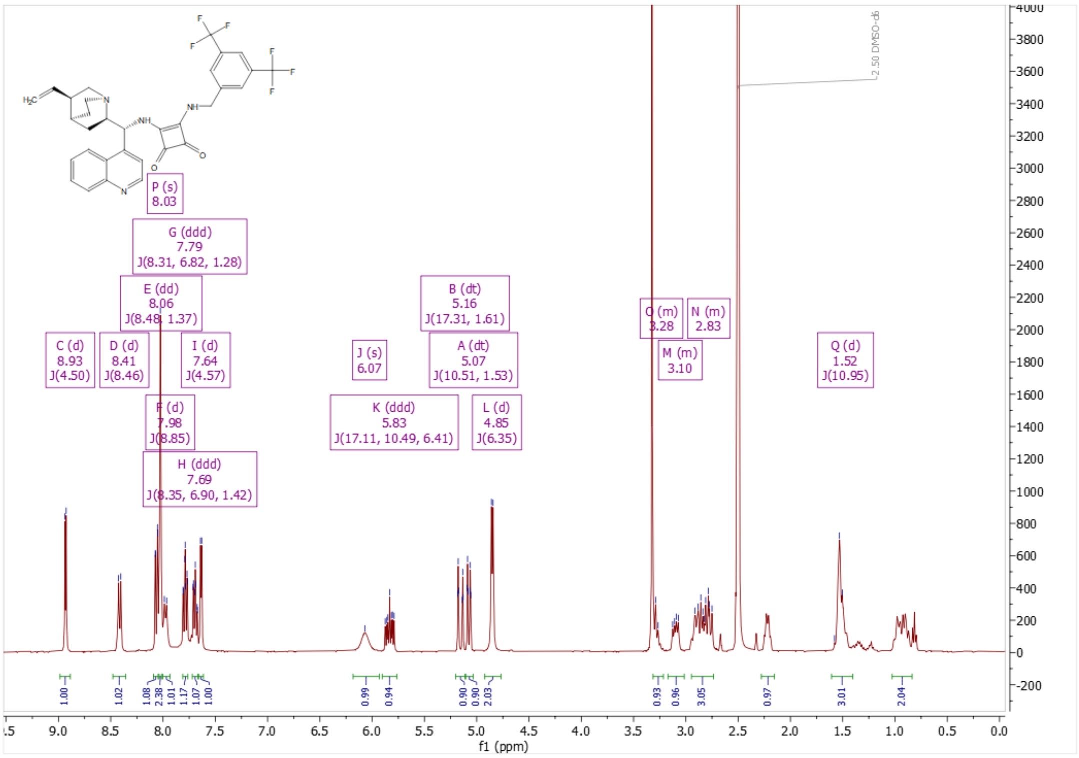

Benzyl (*E*)-3-benzylidene-2-oxoindoline-1-carboxylate (**2b**) ^{1}H, ^{13}C NMR

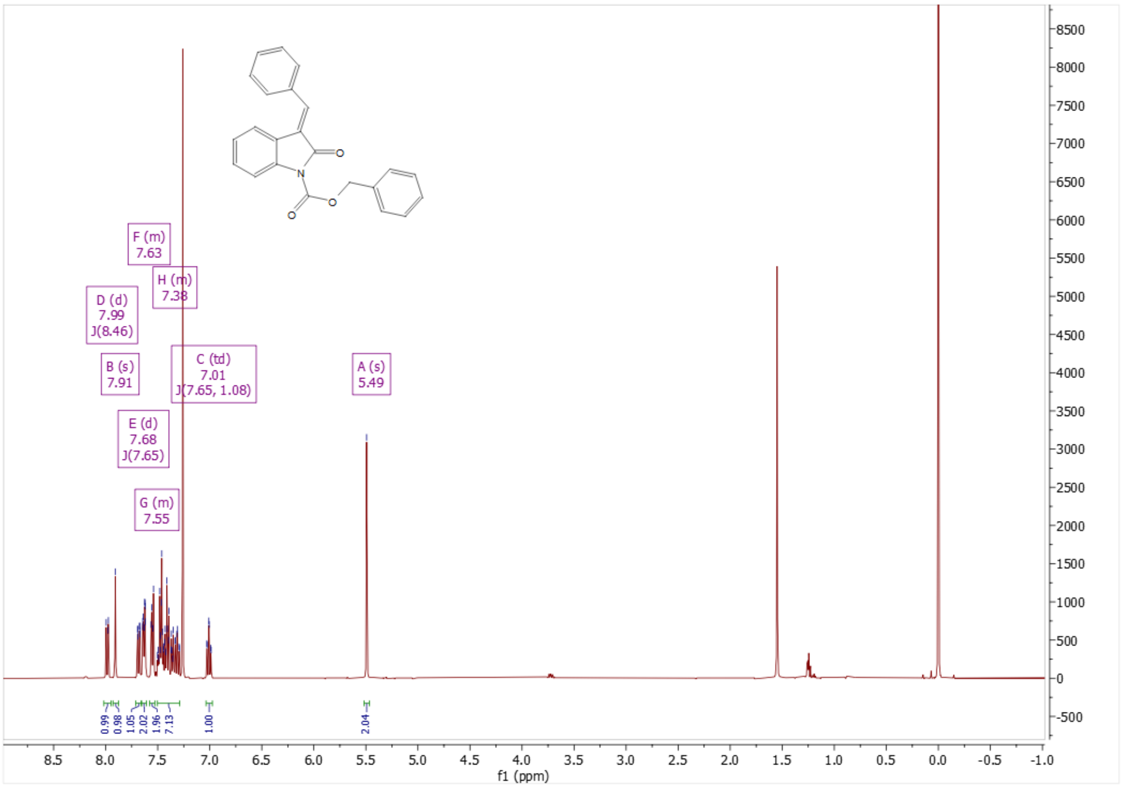

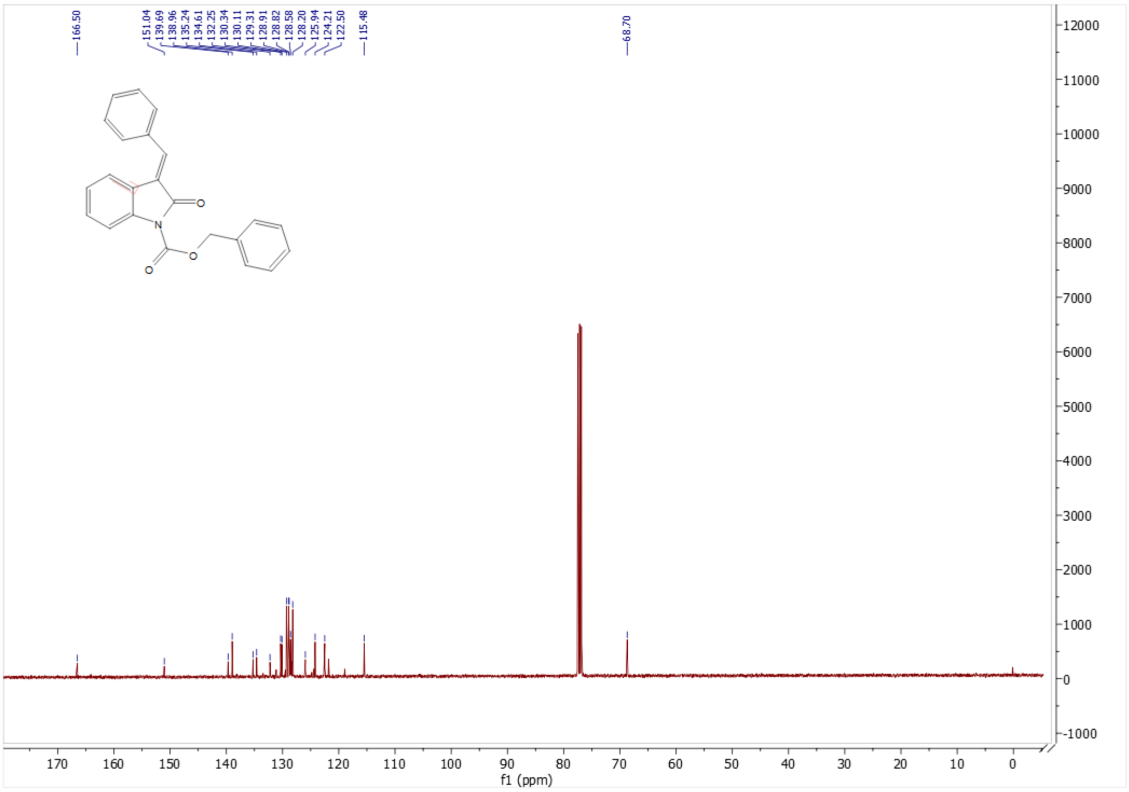

(9*H*-Fluoren-9-yl)methyl (*E*)-3-benzylidene-2-oxoindoline-1-carboxylate (**2c**) ^{1}H, ^{13}C NMR

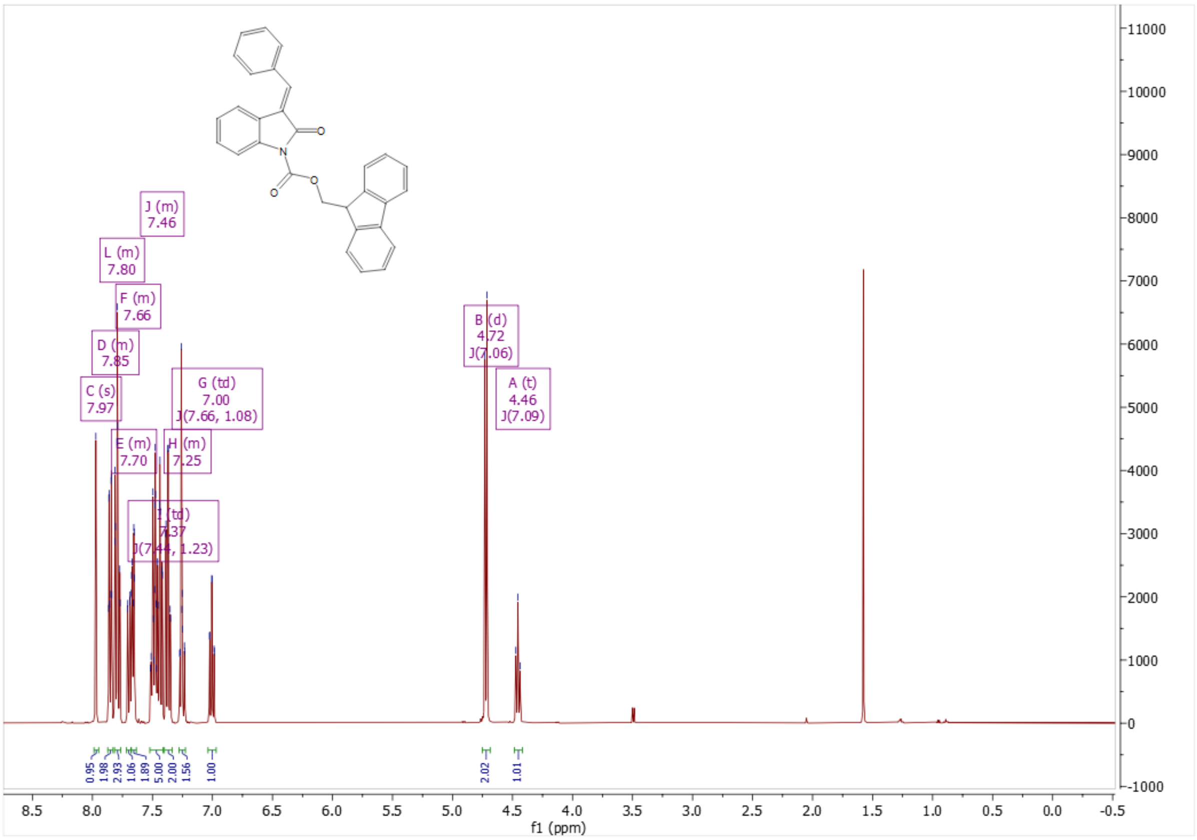

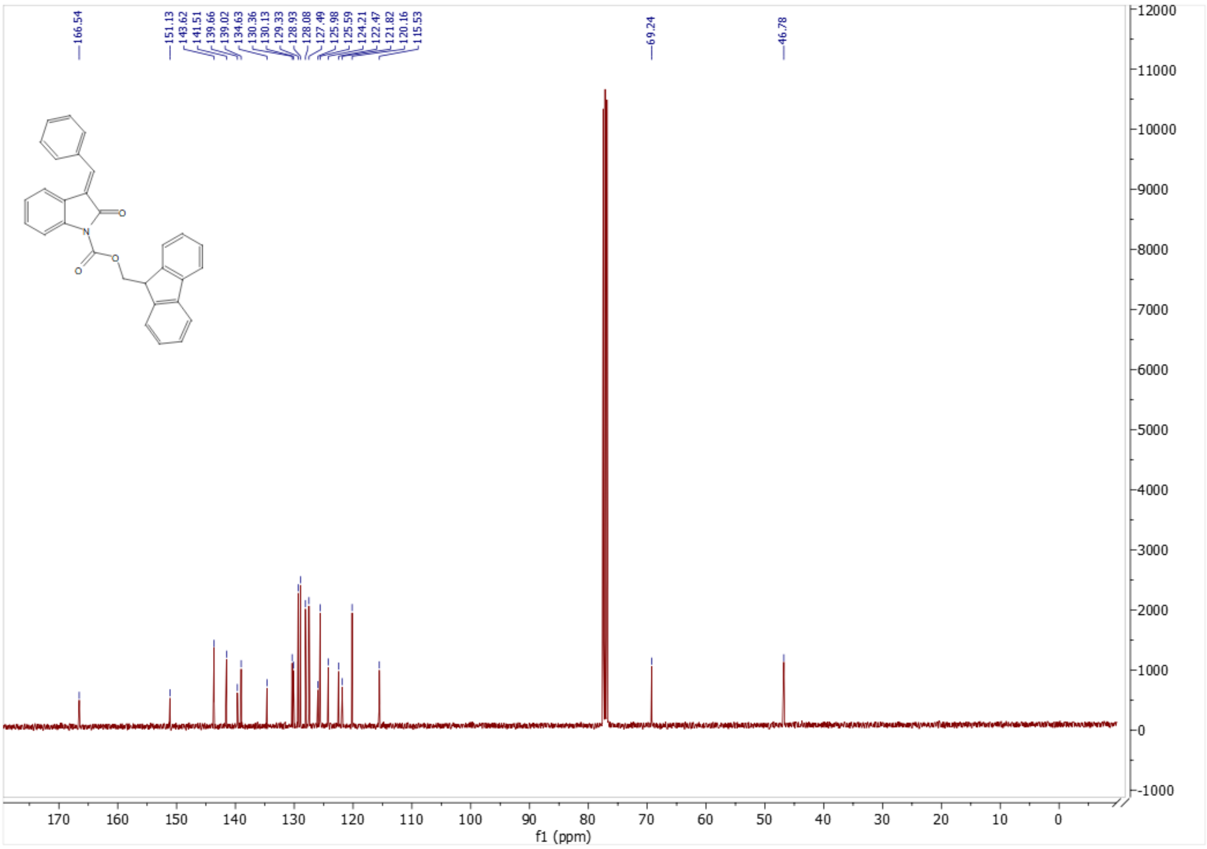

tert-Butyl (*E*)-2-oxo-3-(thiophen-2-ylmethylene)indoline-1-carboxylate (**2j**)

^{1}H, ^{13}C NMR

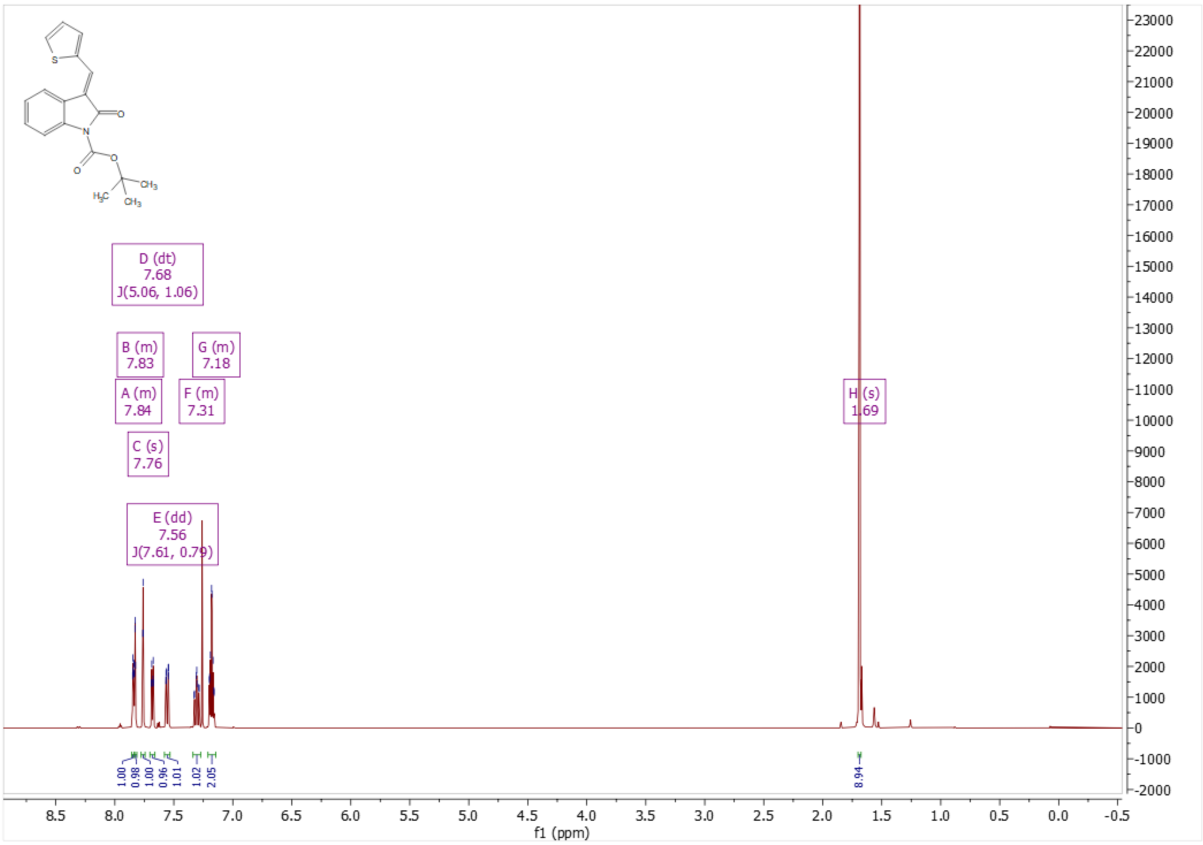

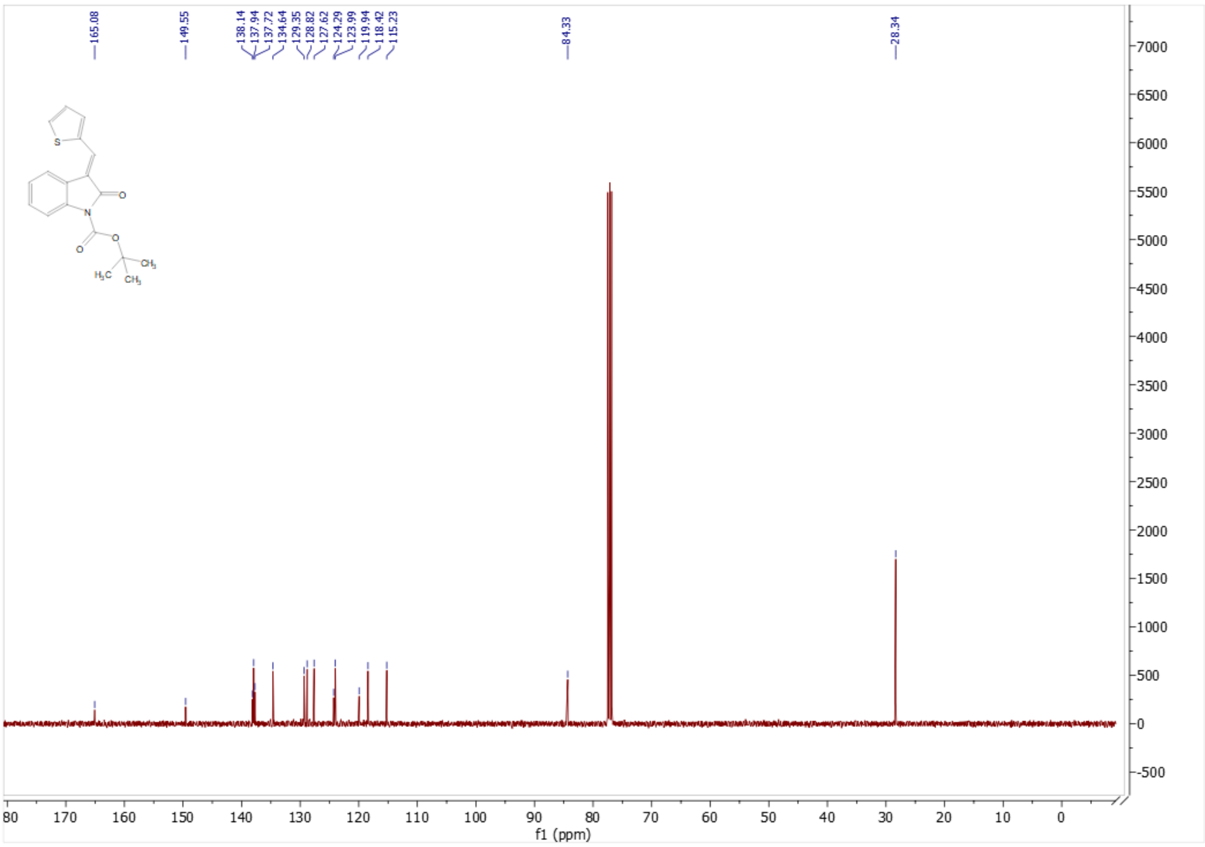

tert-Butyl (*E*)-3-benzylidene-4-bromo-2-oxoindoline-1-carboxylate (**2l**) ^{1}H, ^{13}C NMR

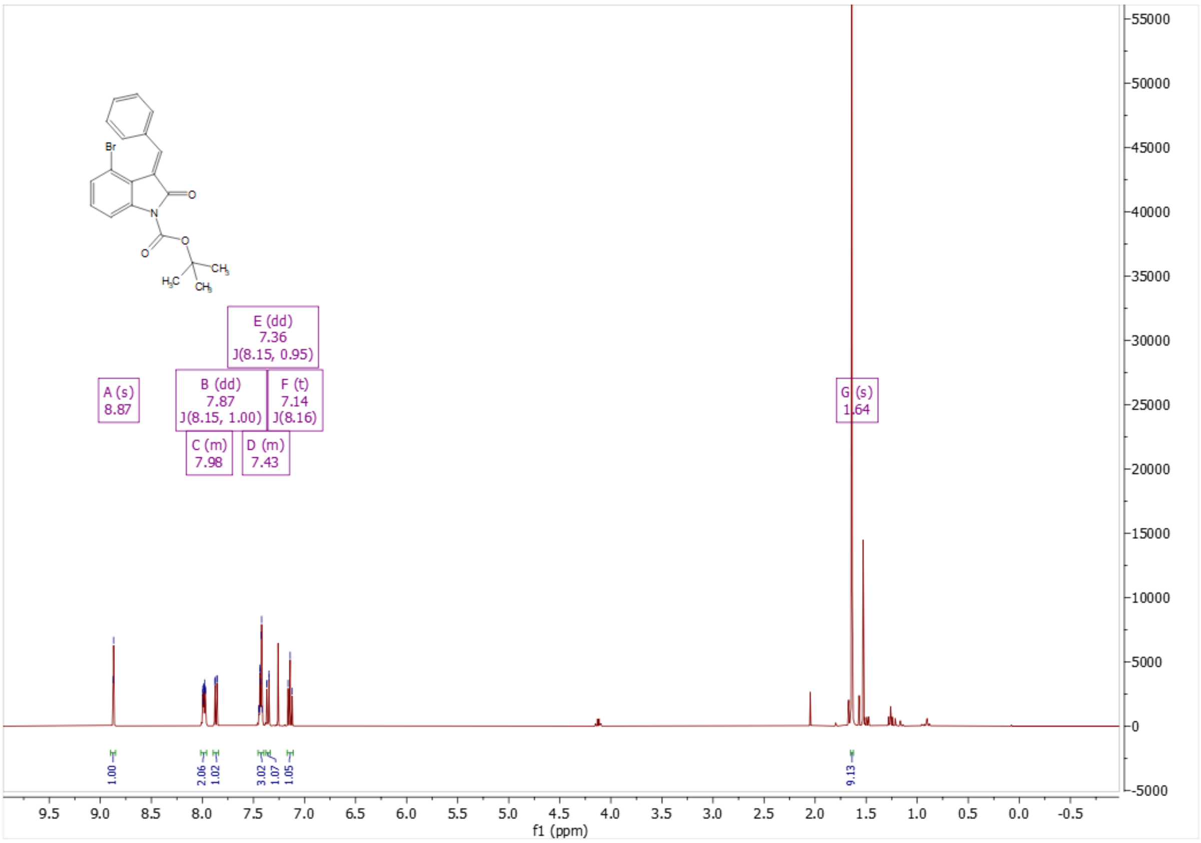

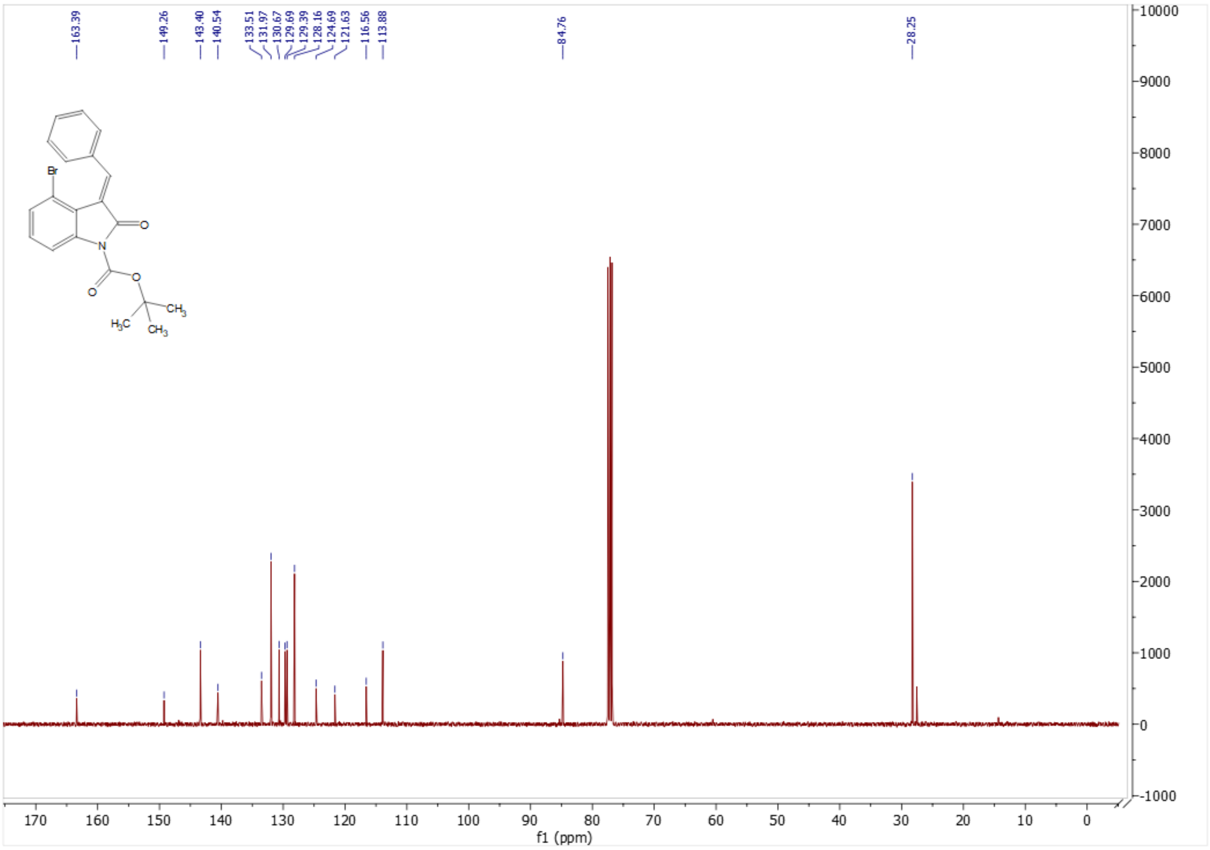

tert-Butyl (*E*)-2-oxo-3-pentylideneindoline-1-carboxylate (**2o**) ^{1}H, ^{13}C NMR

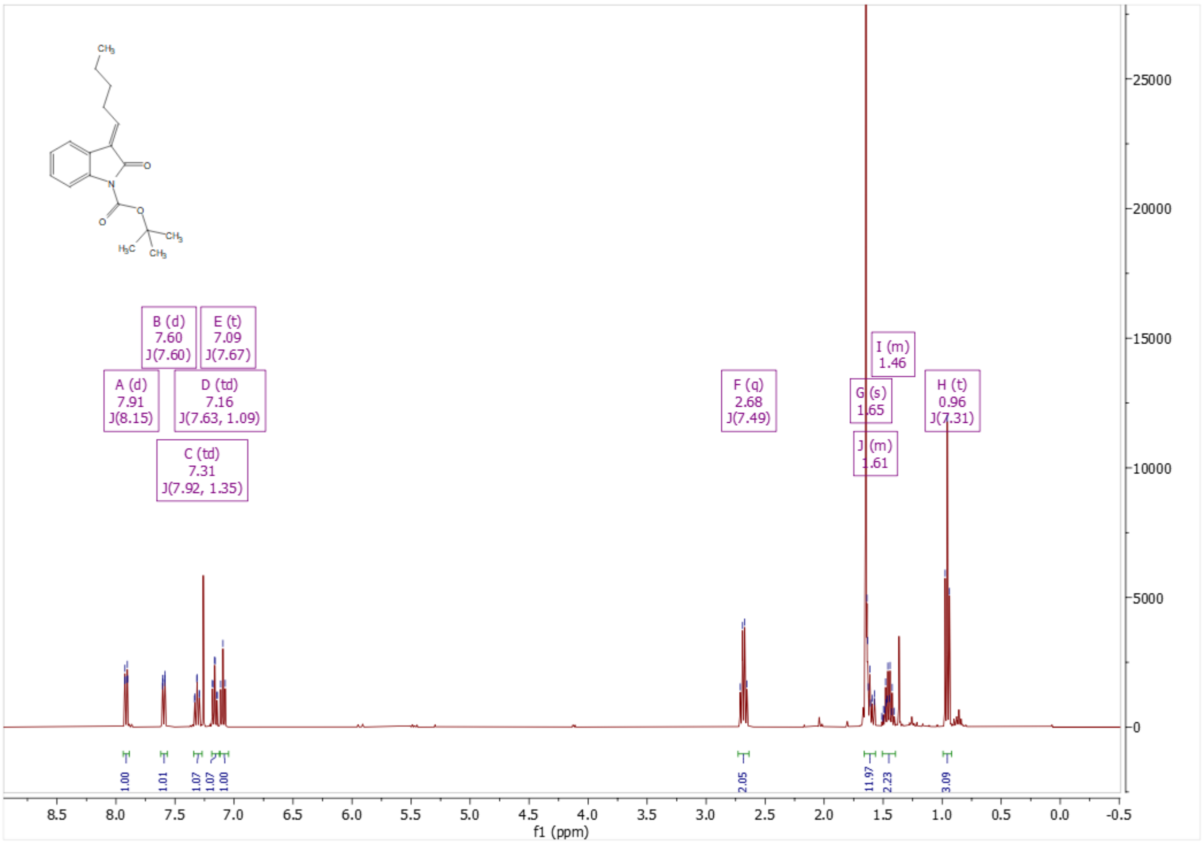

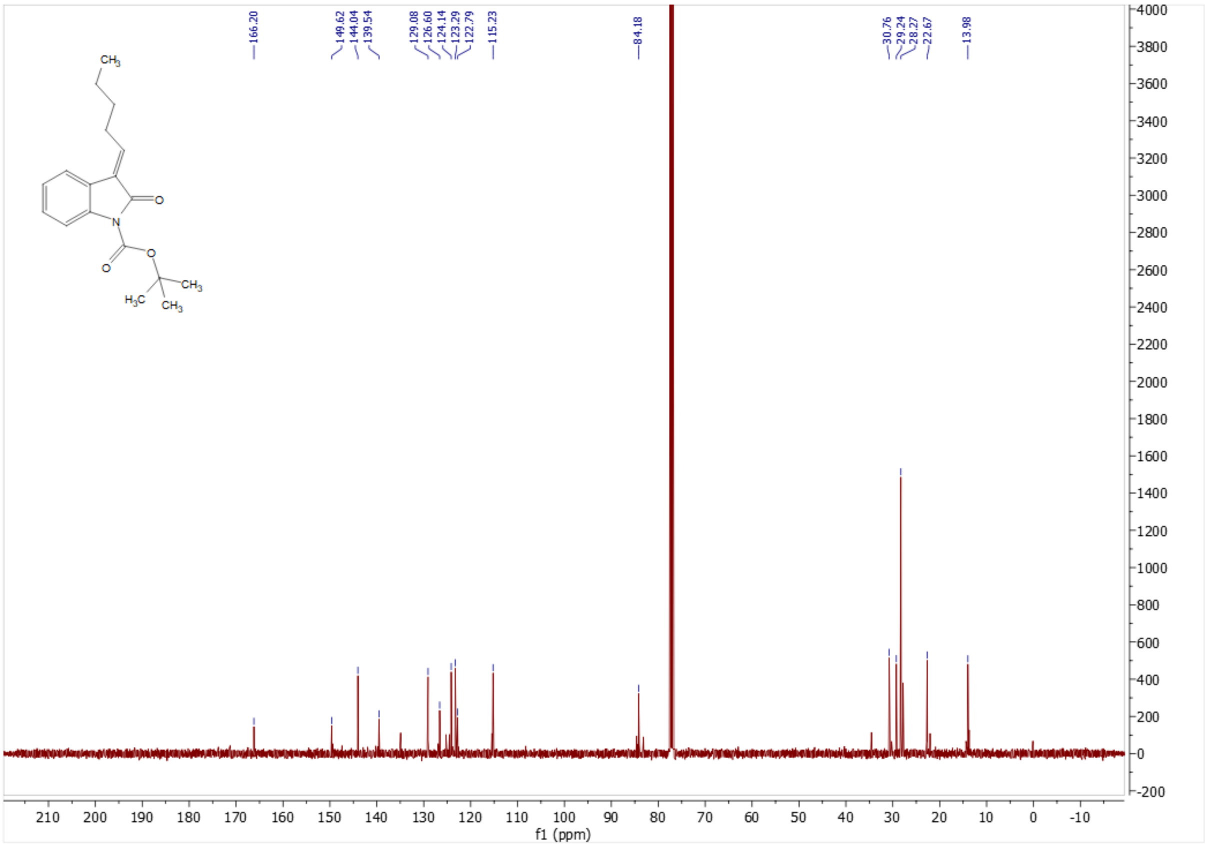

tert-Butyl 3-((2-hydroxy-3-oxocyclopent-1-en-1-yl)(phenyl)methyl)-2-oxoindolin-1-carboxylate (**3a**), mixture of diastereoisomers, ^{1}H, ^{13}C NMR

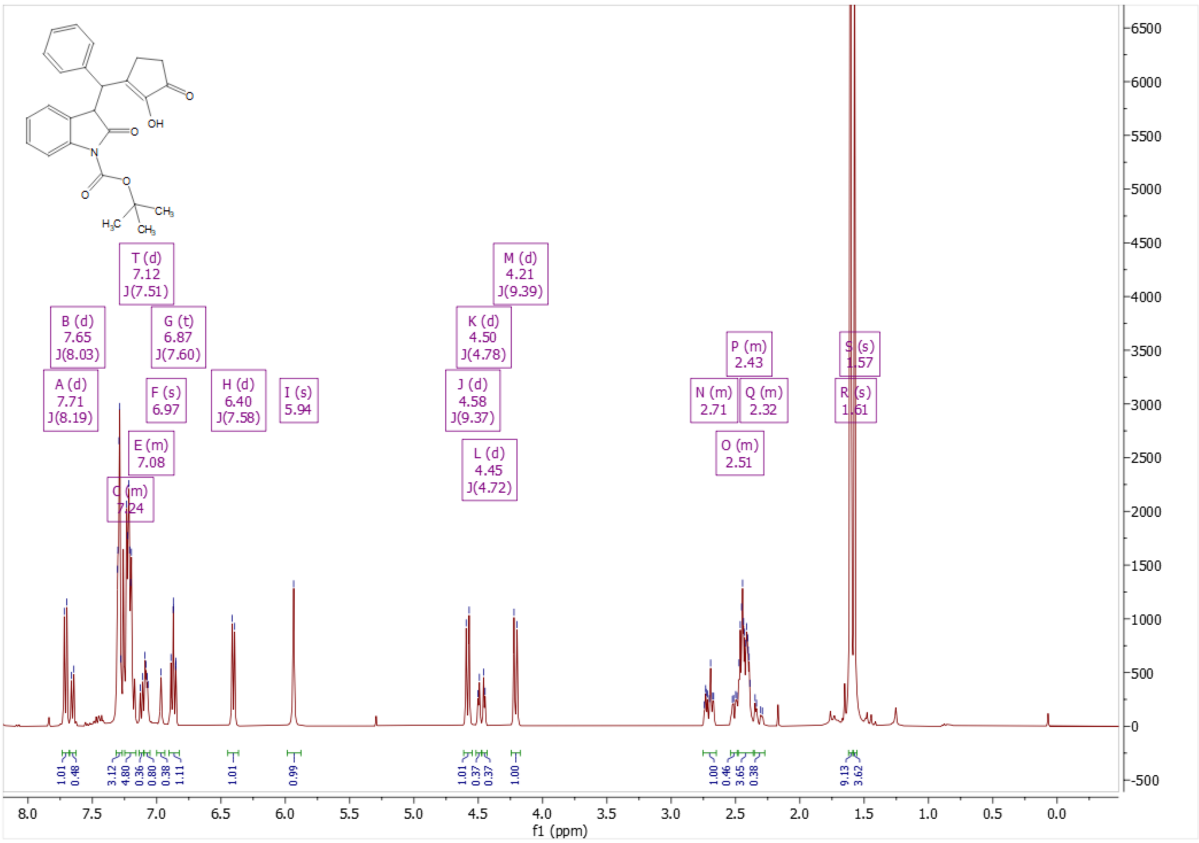

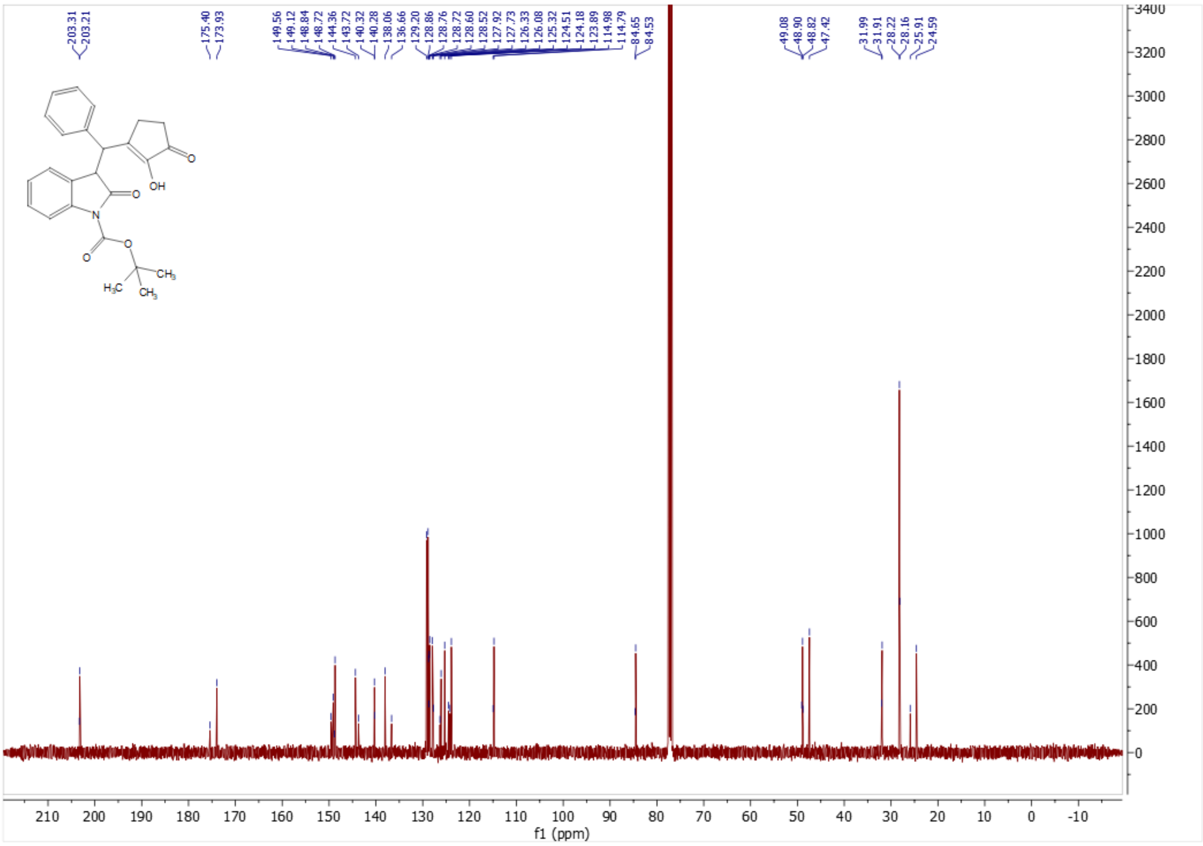

Benzyl 3-((2-hydroxy-3-oxocyclopent-1-en-1-yl)(phenyl)methyl)-2-oxoindoline-1-carbocylate (**3b**), mixture of diastereoisomers, ^{1}H, ^{13}C NMR

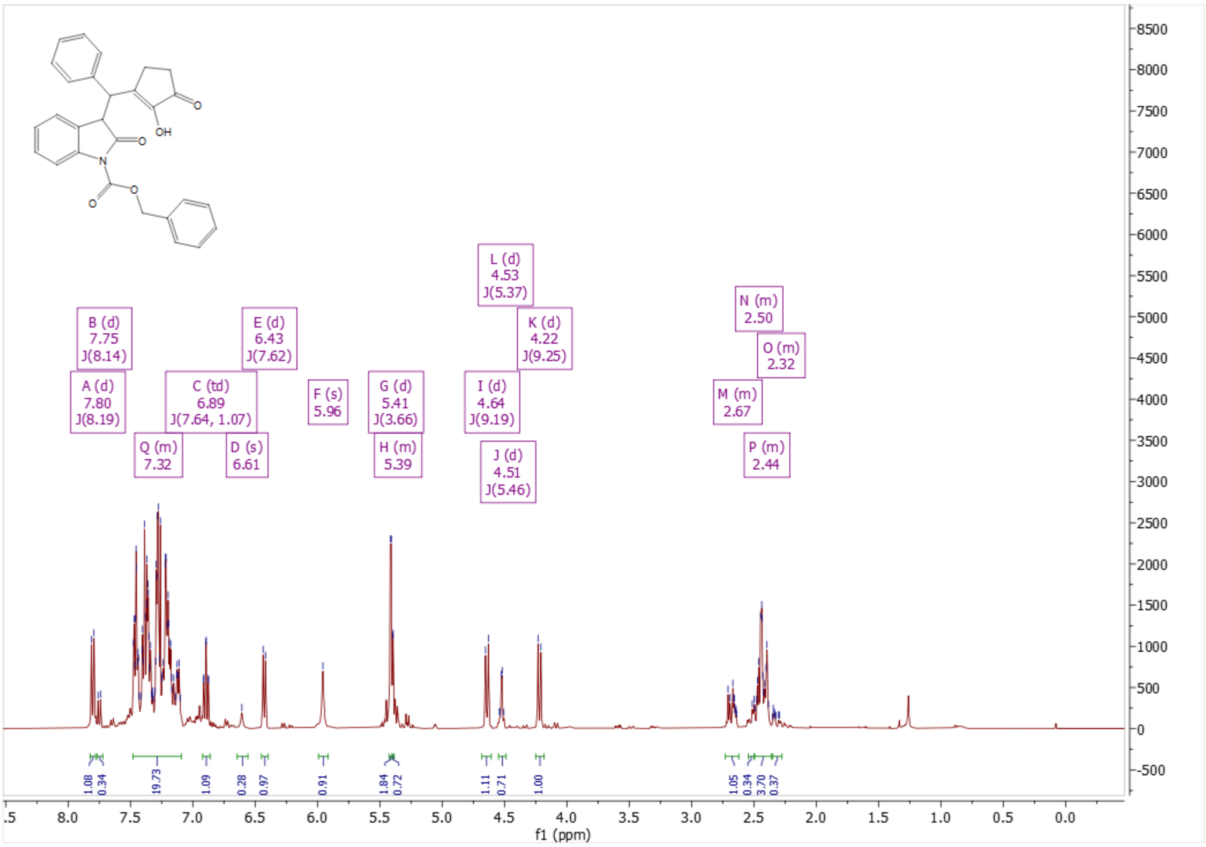

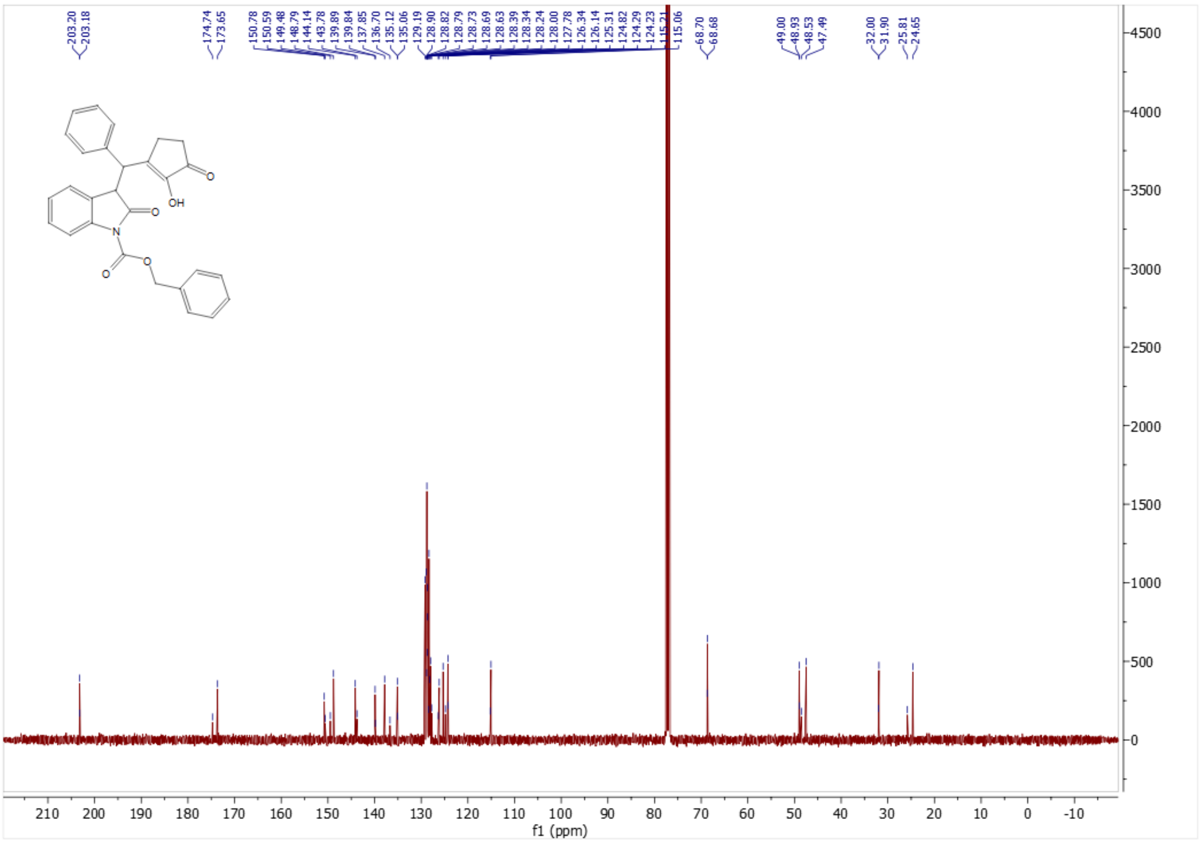

(9*H*-Fluoren-9-yl)methyl 3-((2-hydroxy-3-oxocyclopent-1-en-1-yl)(phenyl)methyl)-2-oxoindoline-1-carboxylate (**3c**), mixture of diastereoisomers, ^{1}H, ^{13}C NMR

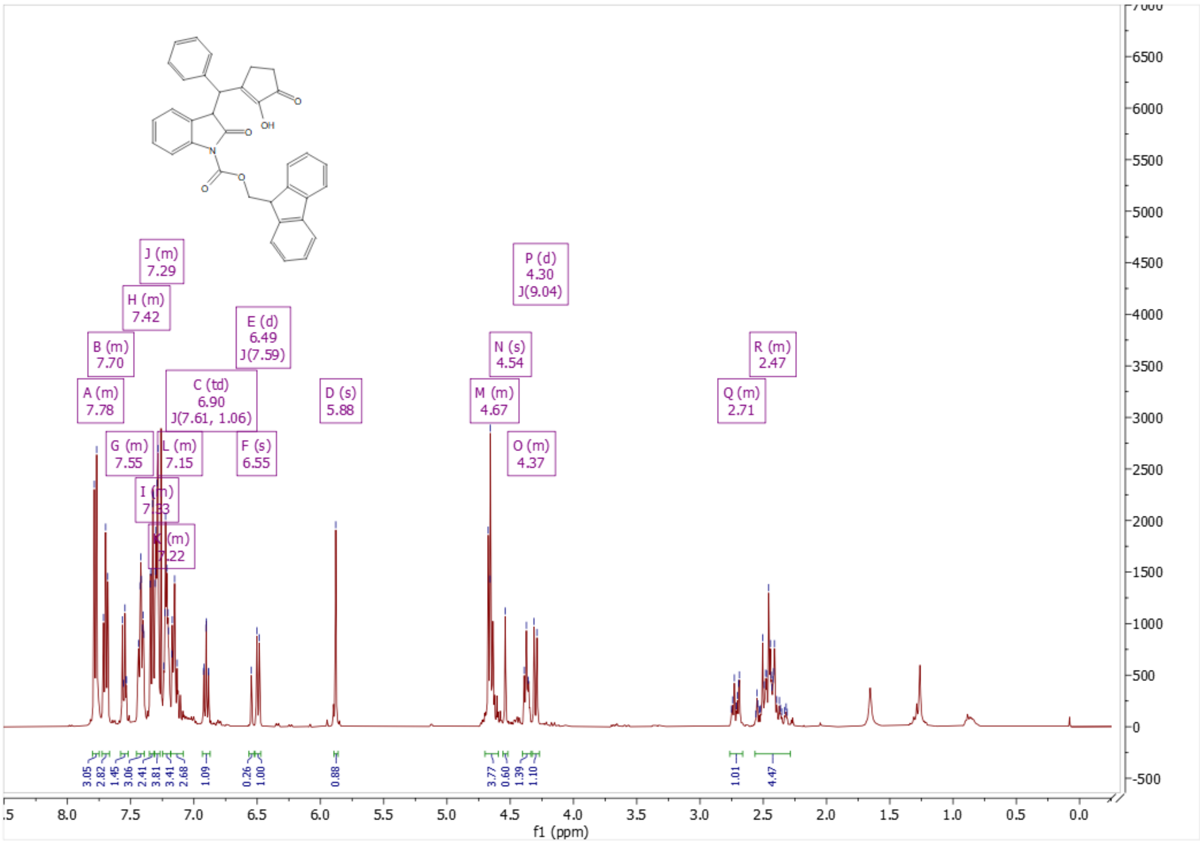

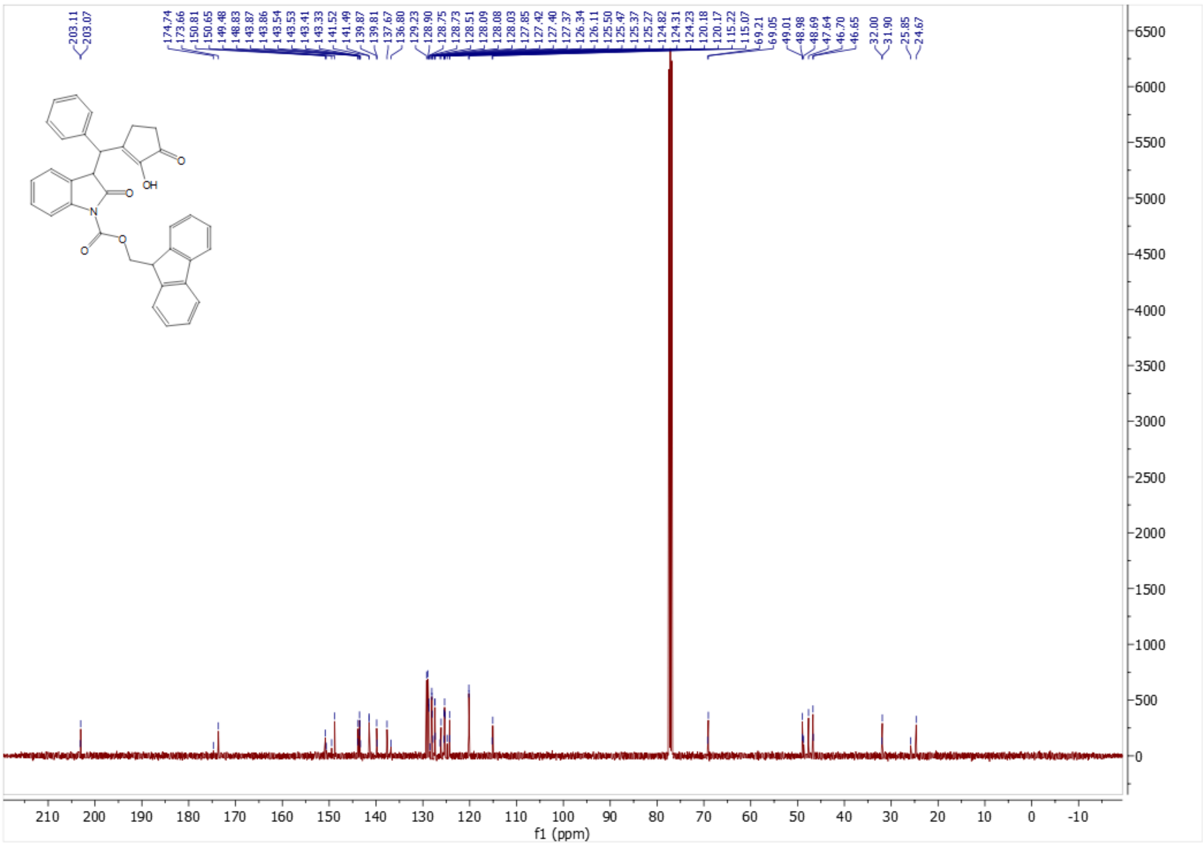

3-((2-Hydroxy-3-oxocyclopent-1-en-1-yl)(phenyl)methyl-1-tosylindolin-2-one (**3e**), mixture of diastereoisomers, ^{1}H, ^{13}C NMR

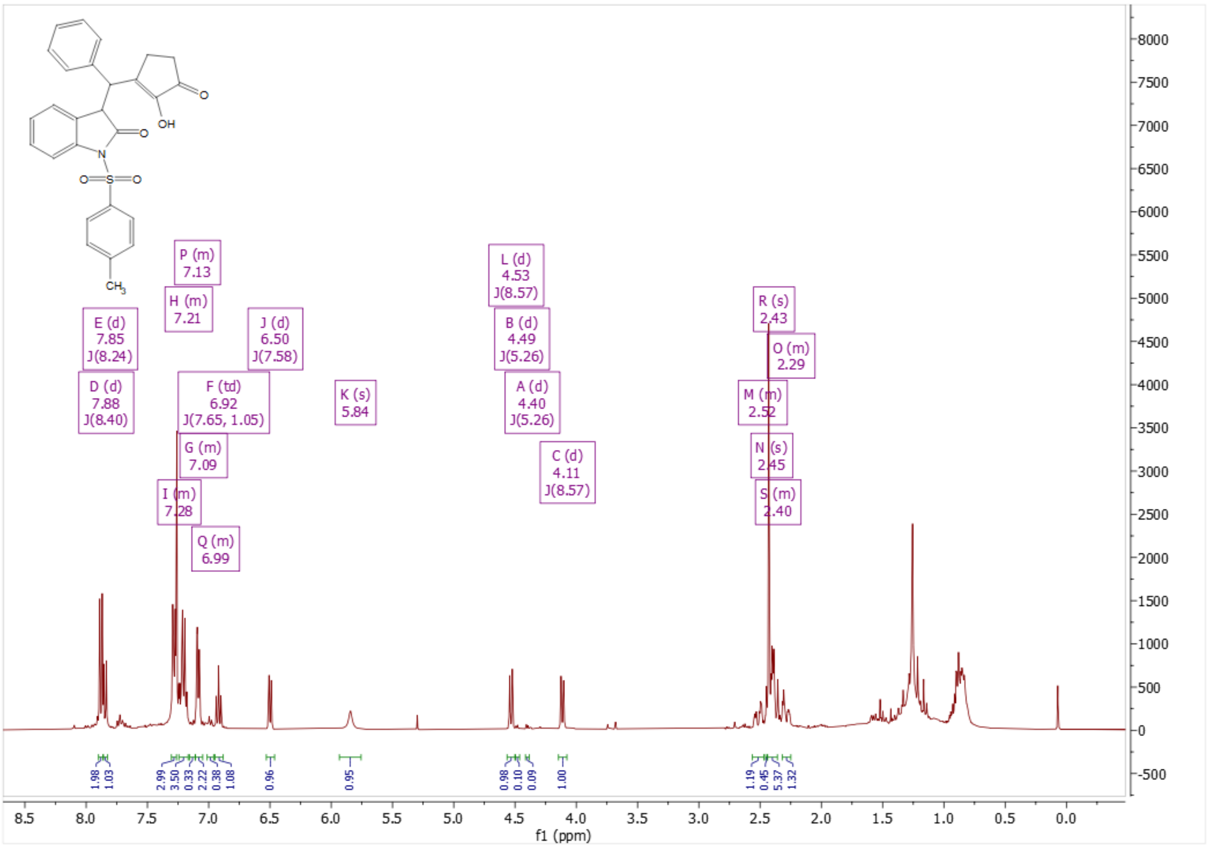

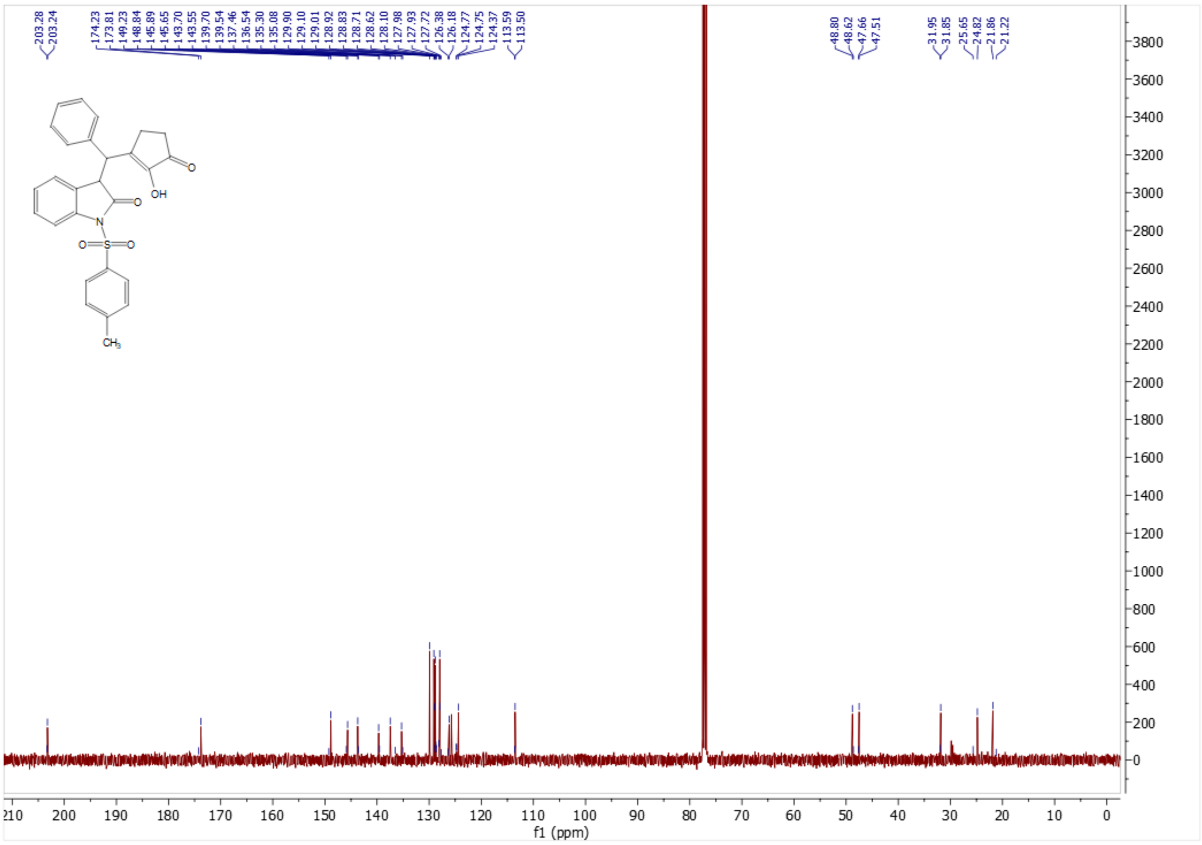

tert-Butyl 3-((2-chlorophenyl)(2-hydroxy-3-oxocyclopent-1-en-1-yl)methyl)-2-oxoindoline-1-carboxylate (**3f**), mixture of diastereoisomers, ^{1}H, ^{13}C NMR

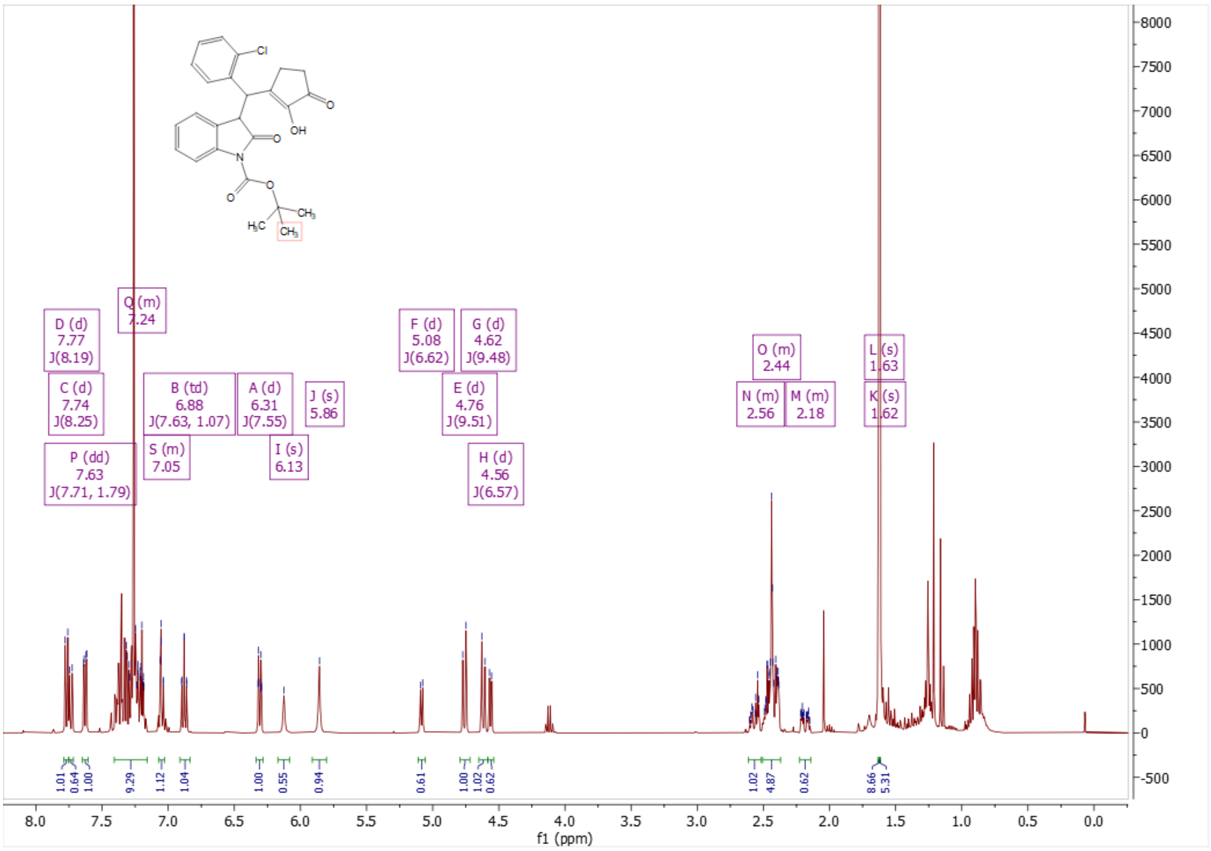

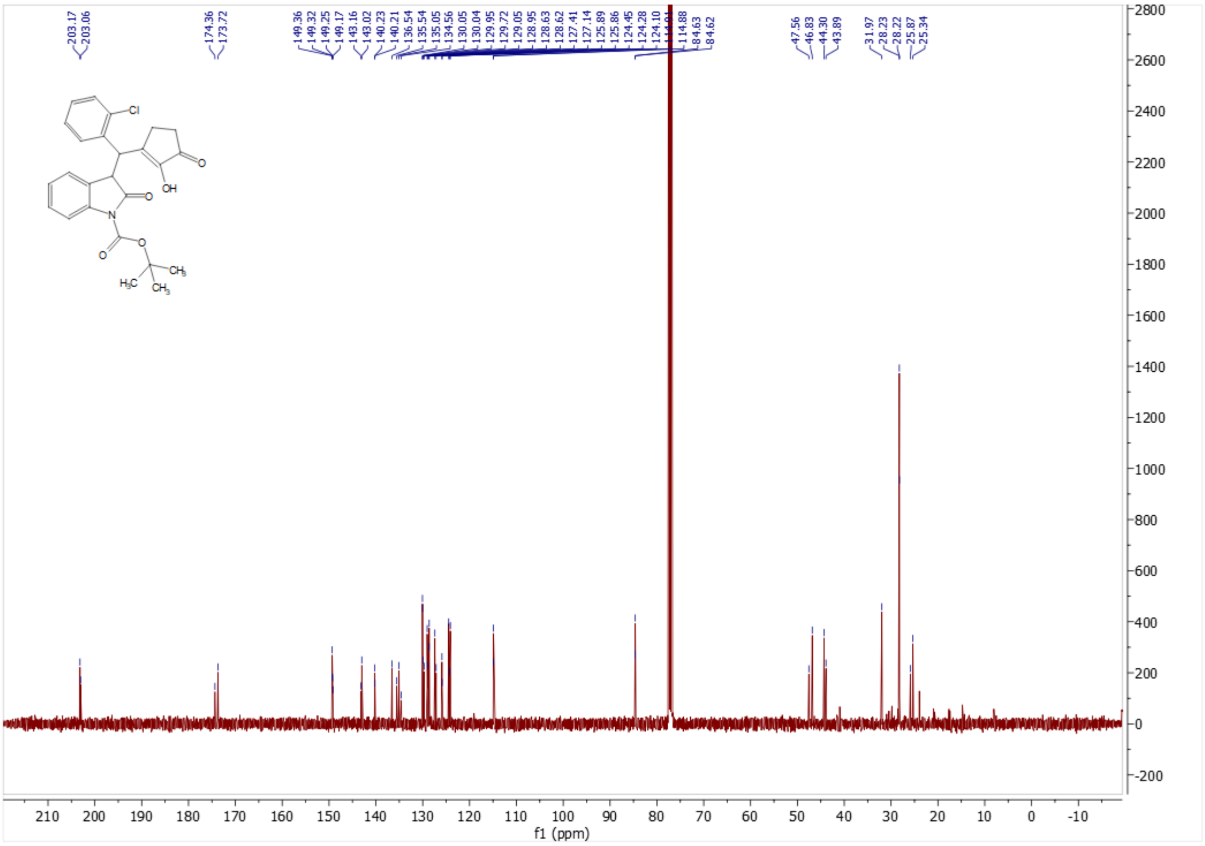

tert-Butyl 3-((3-chlorophenyl)(2-hydroxy-3-oxocyclopent-1-en-1-yl)methyl)-2-oxoindoline-1-carboxylate (**3g**), mixture of diastereoisomers, ^{1}H, ^{13}C NMR

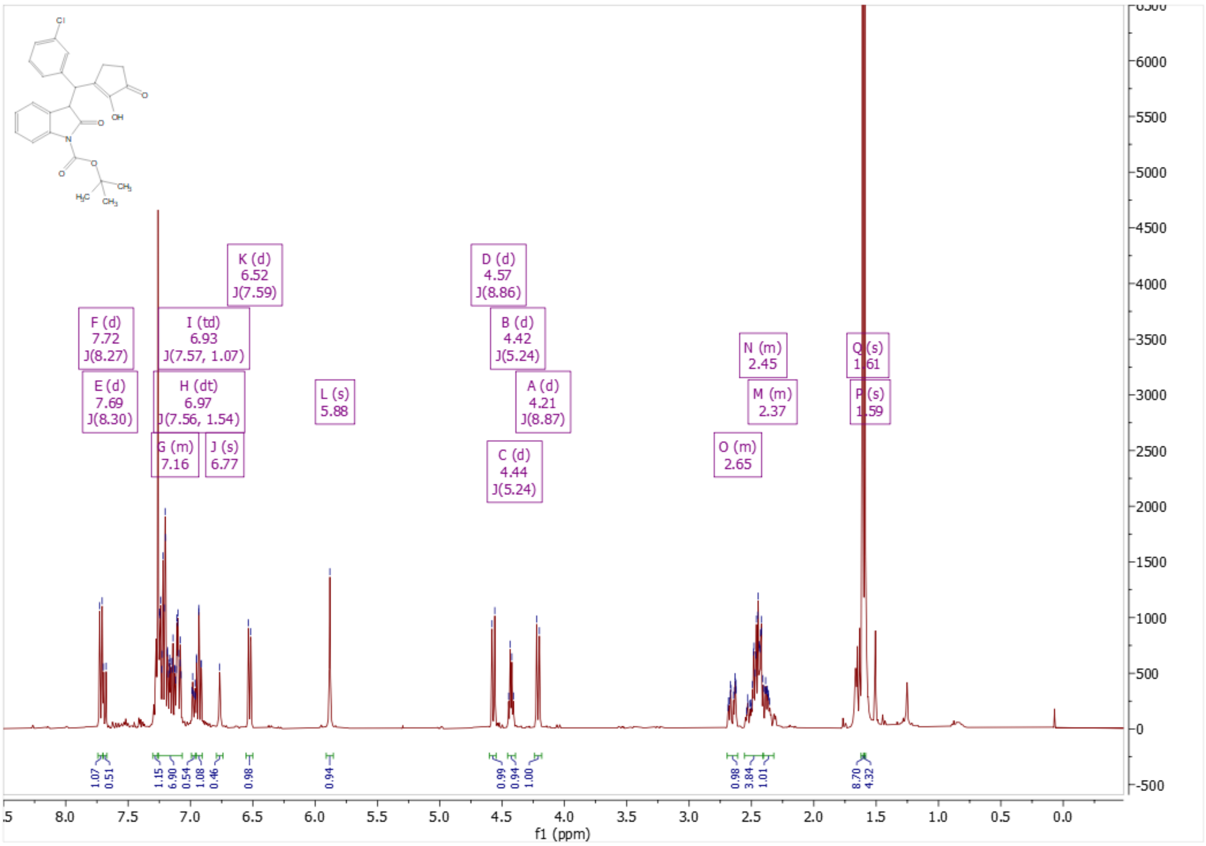

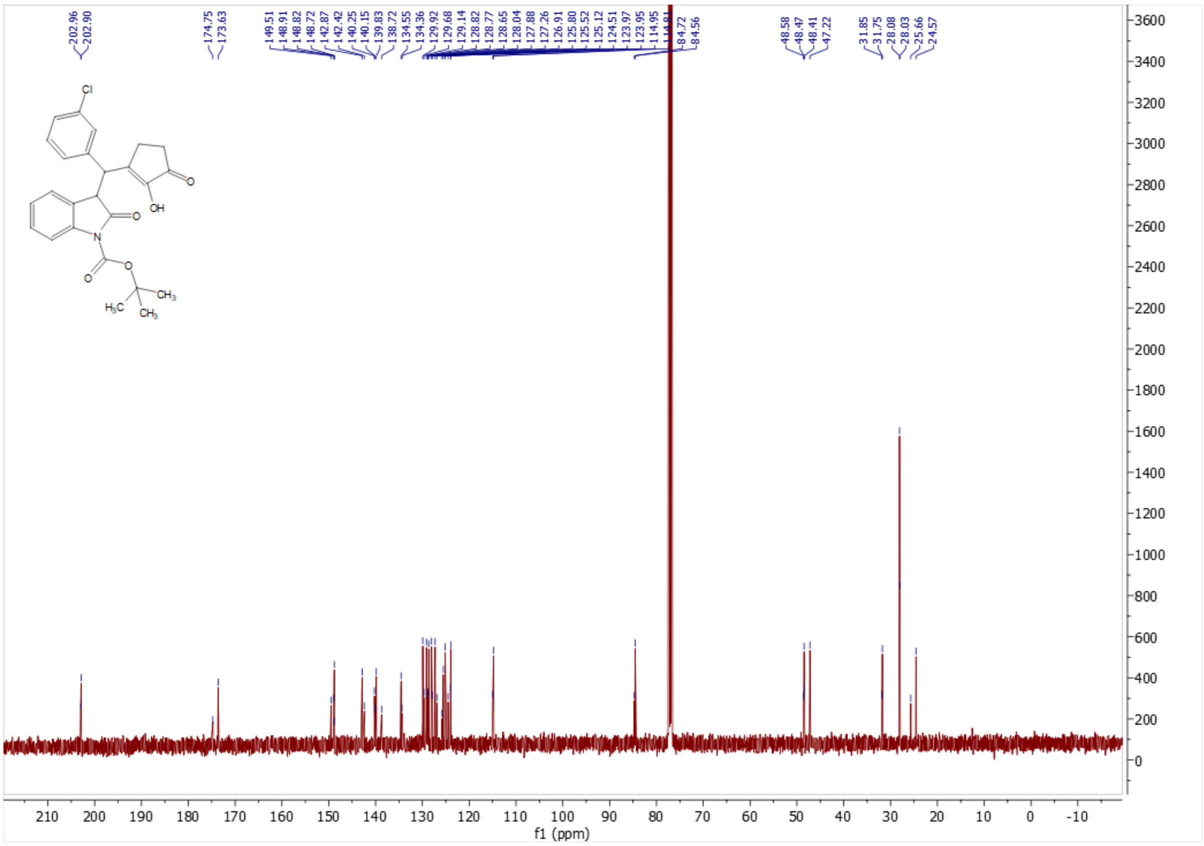

tert-Butyl 3-((4-chlorophenyl)(2-hydroxy-3-oxocyclopent-1-en-1-yl)methyl)-2-oxoindoline-1-carboxylate (**3h**), mixture of diastereoisomers, ^{1}H, ^{13}C NMR

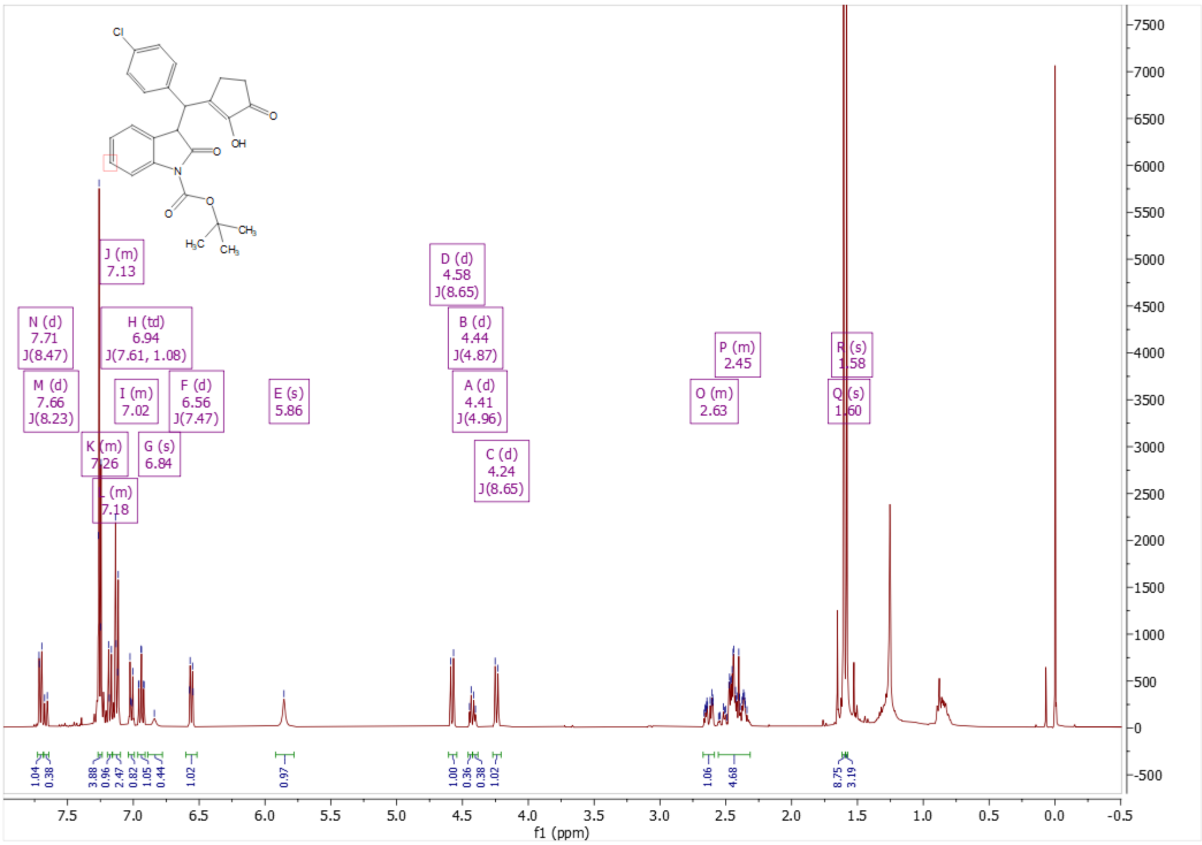

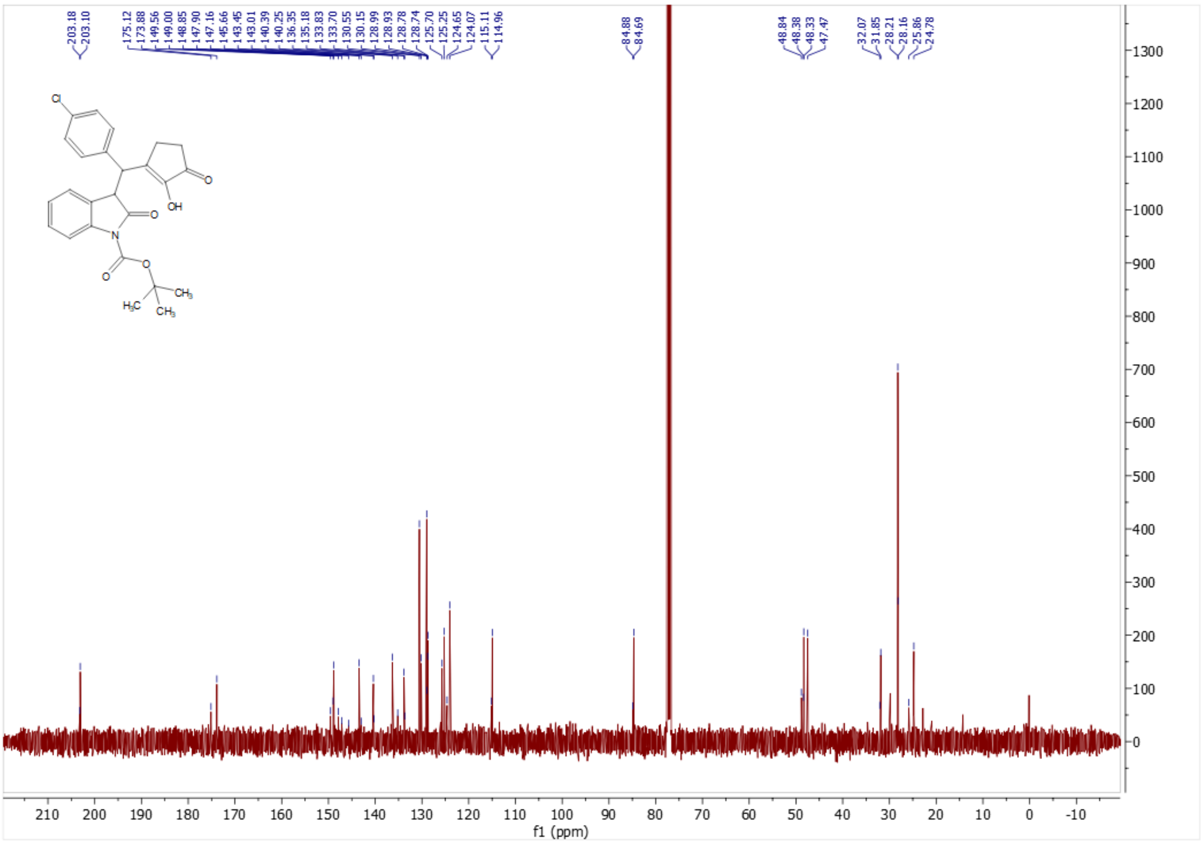

tert-Butyl 3-(2-ethoxy-1-(2-hydroxy-3-oxocyclopent-1-en-1-yl)-2-oxoethyl)-2-oxoindoline-1-carboxylate (**3i**), mixture of diastereoisomers, ^{1}H, ^{13}C NMR

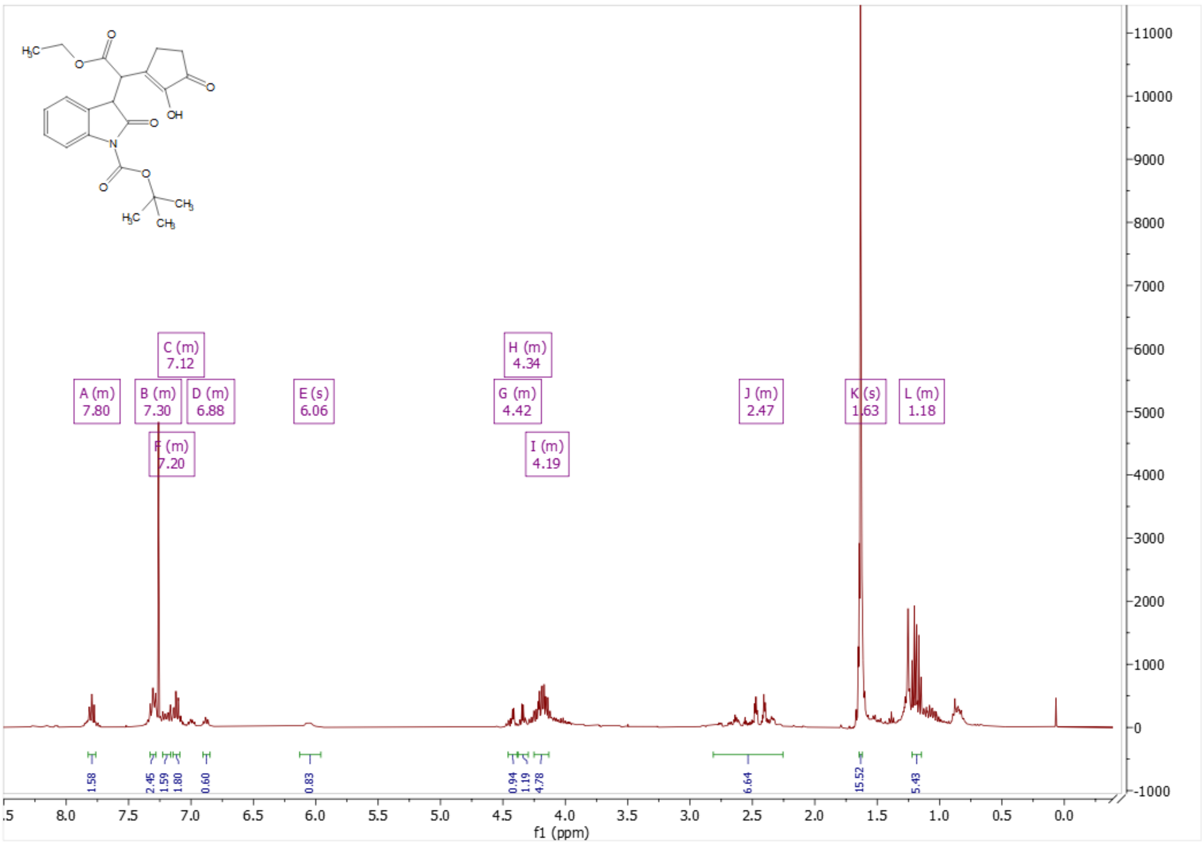

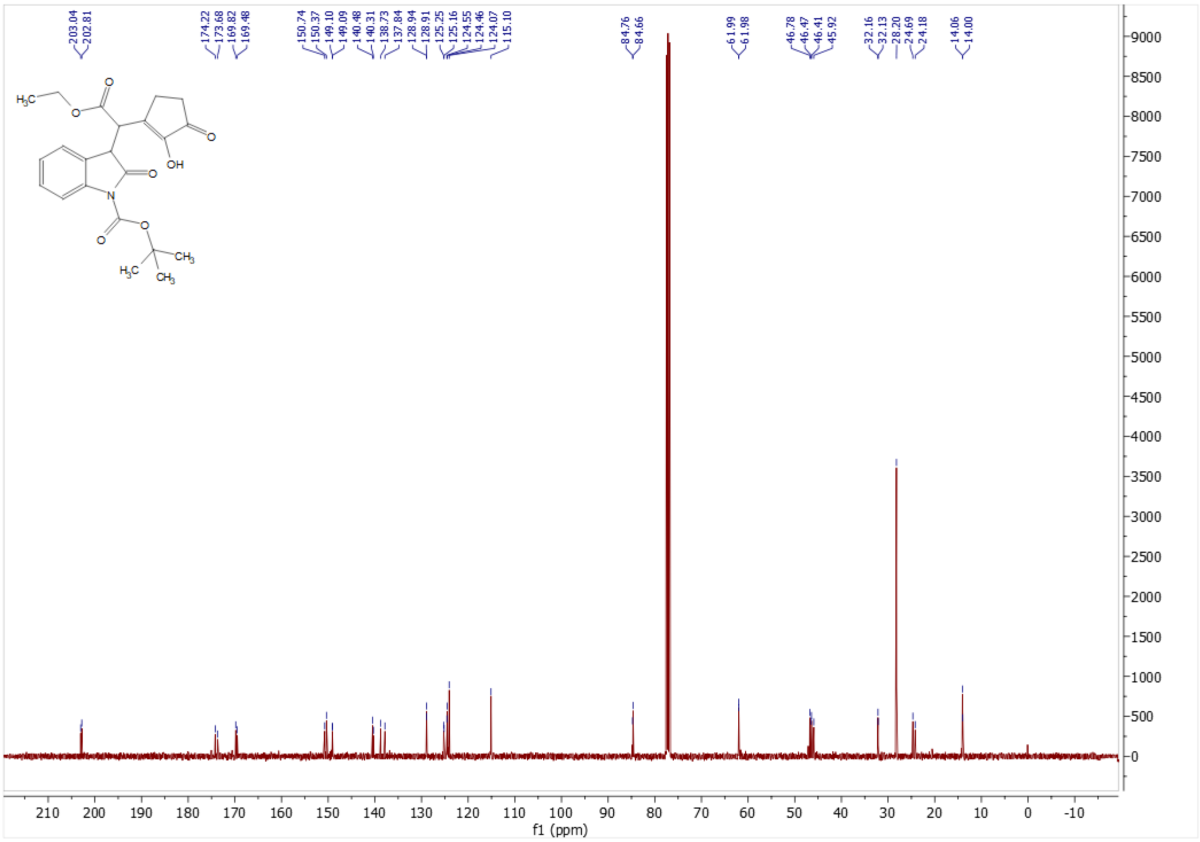

tert-Butyl 3-((2-hydroxy-3-oxocyclopent-1-en-1-yl)(thiophen-2-yl)methyl)-2-oxoindoline-1-carboxylate (**3j**), mixture of diastereoisomers, ^{1}H, ^{13}C NMR

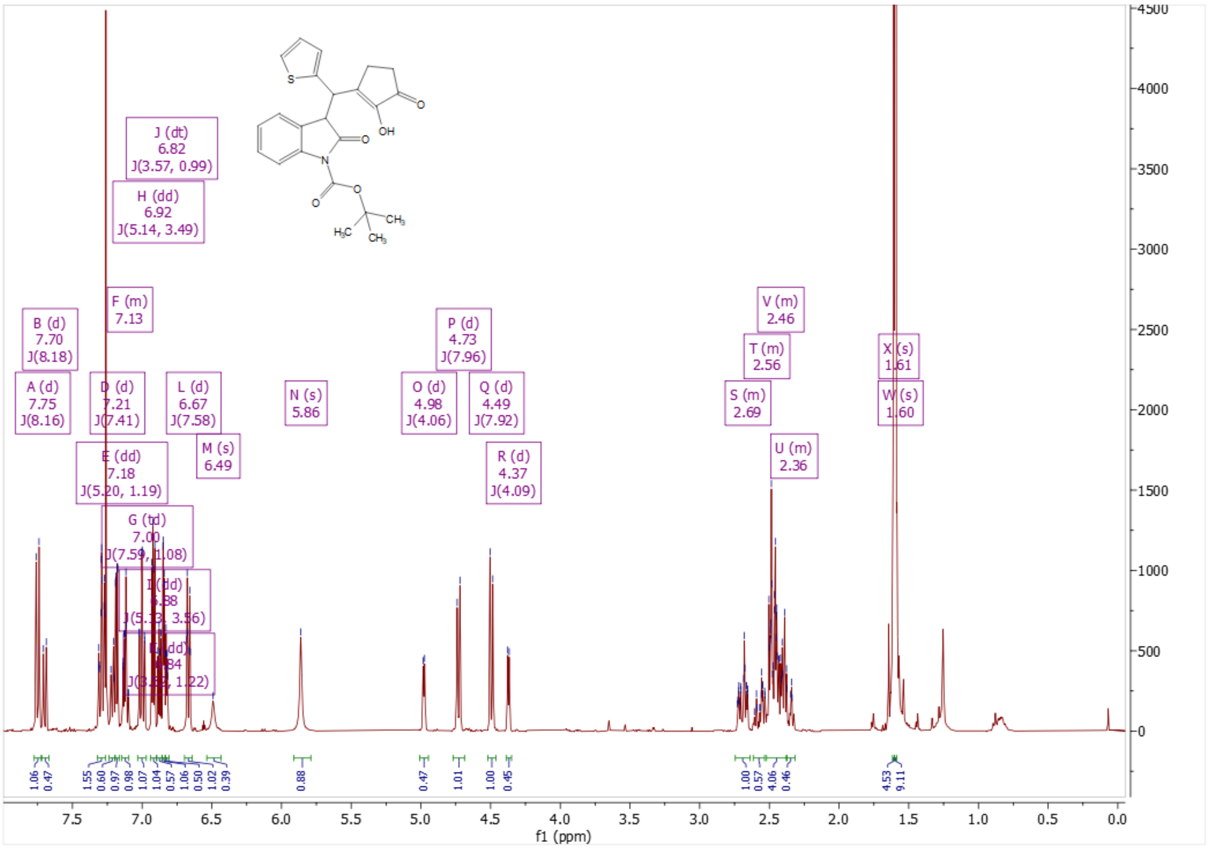

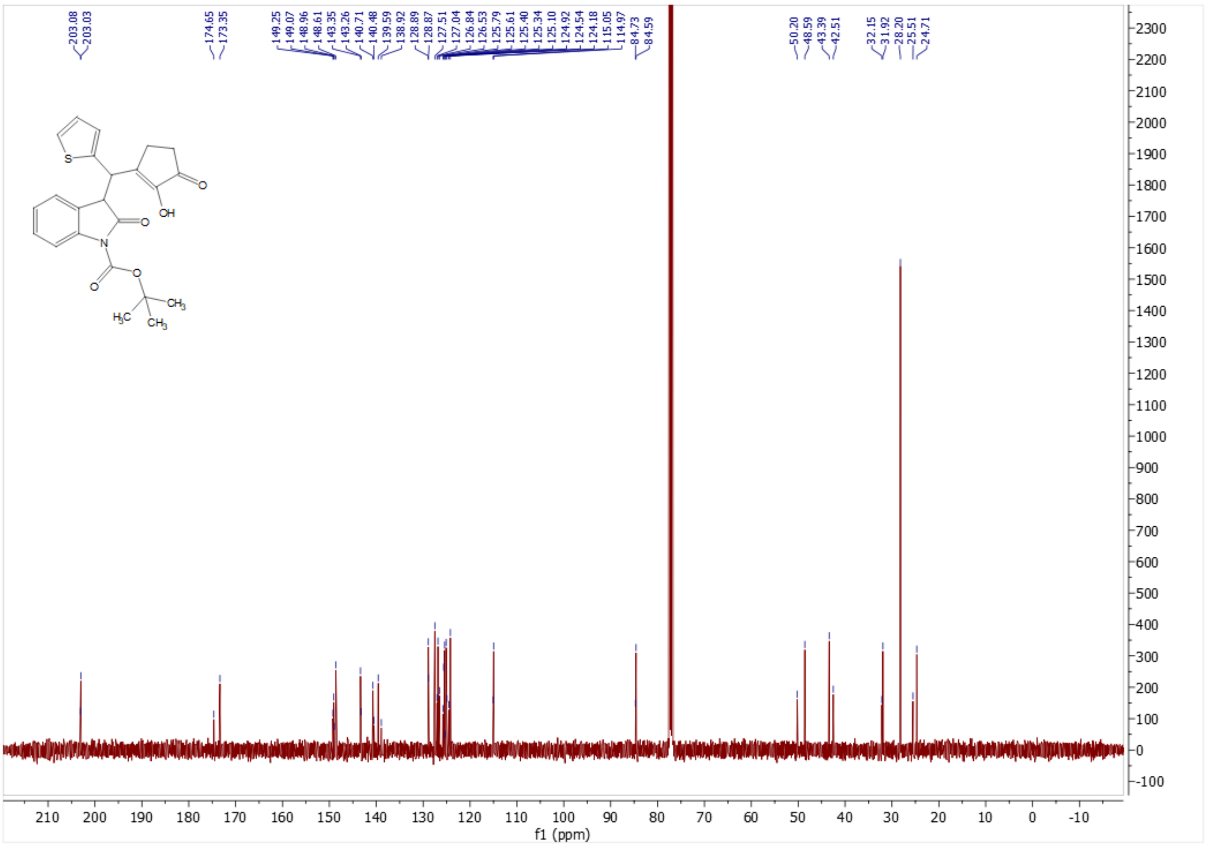

tert-Butyl 5-bromo-3-((2-hydroxy-3-oxocyclopent-1-en-1-yl)(phenyl)methyl)-2-oxoindoline-1-carboxylate (**3k**), mixture of diastereoisomers, ^{1}H, ^{13}C NMR

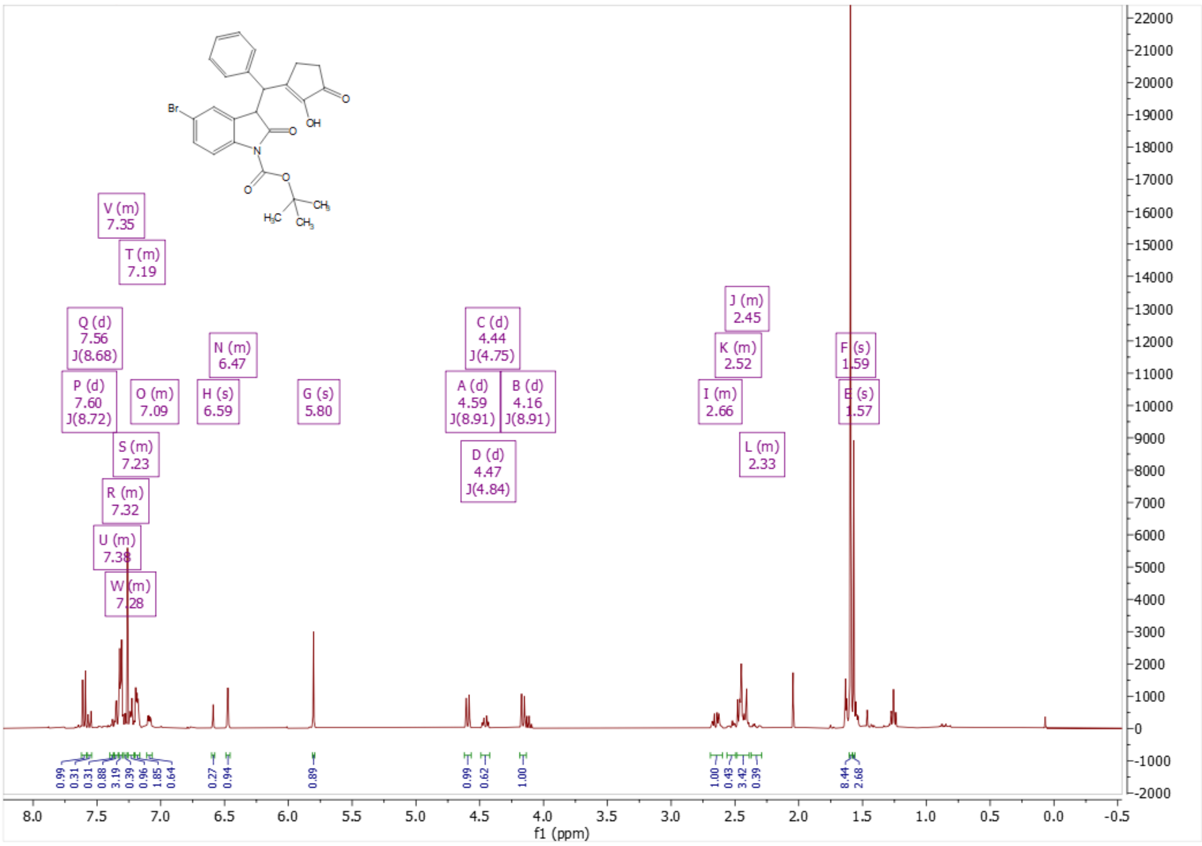

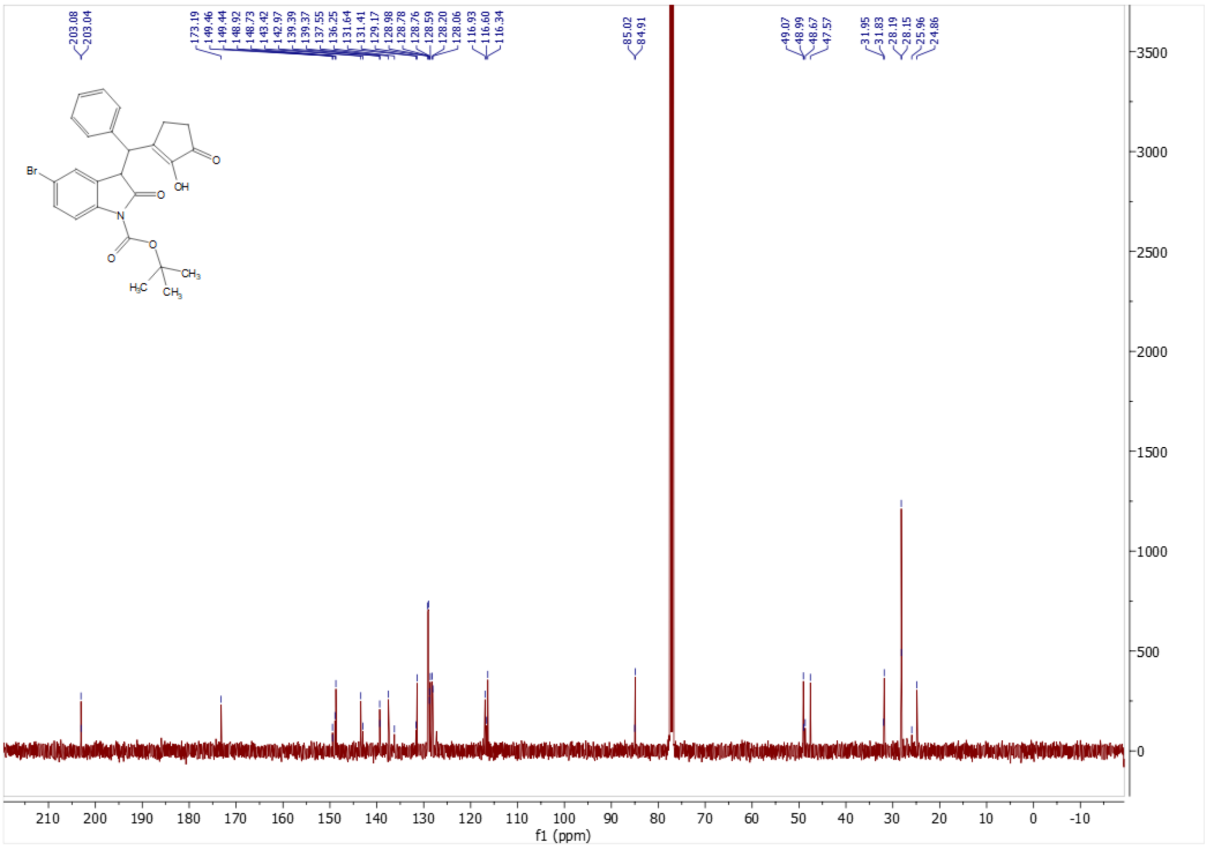

tert-Butyl 4-bromo-3-((2-hydroxy-3-oxocyclopent-1-en-1-yl)(phenyl)methyl)-2-oxoindoline-1-carboxylate (**3l**), mixture of diastereoisomers, ^{1}H, ^{13}C NMR

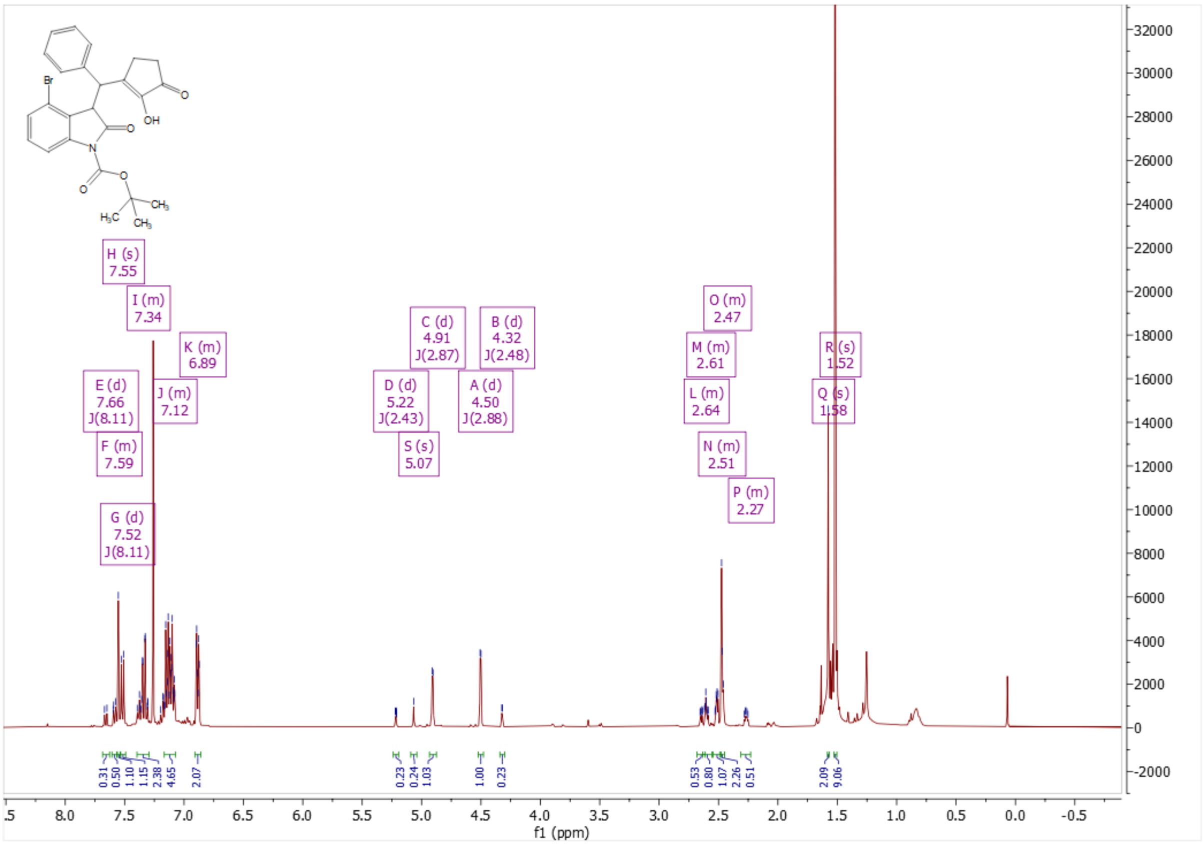

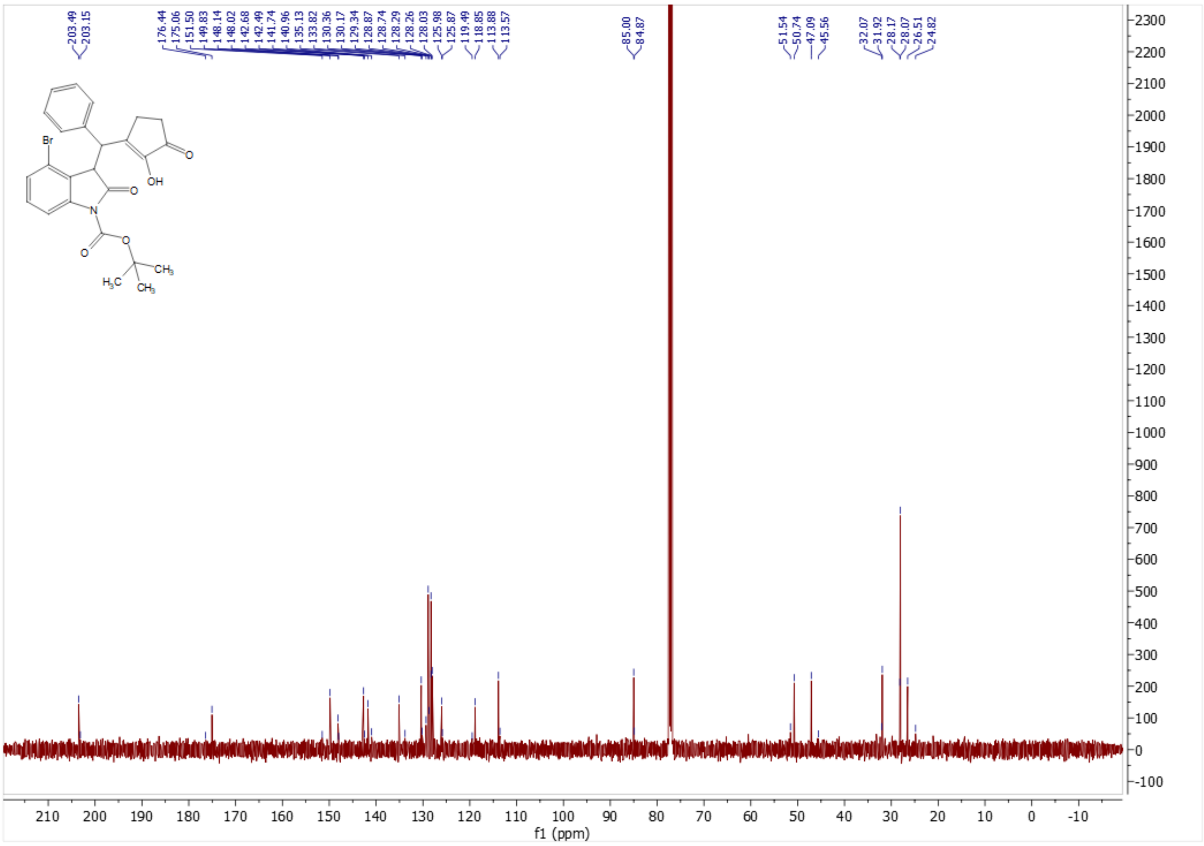

tert-Butyl 3-((2-hydroxy-3-oxocyclopent-1-en-1-yl)(4-methoxyphenyl)methyl)-2-oxoindoline-1-carboxylate (**3m**), mixture of diastereoisomers, ^{1}H, ^{13}C NMR

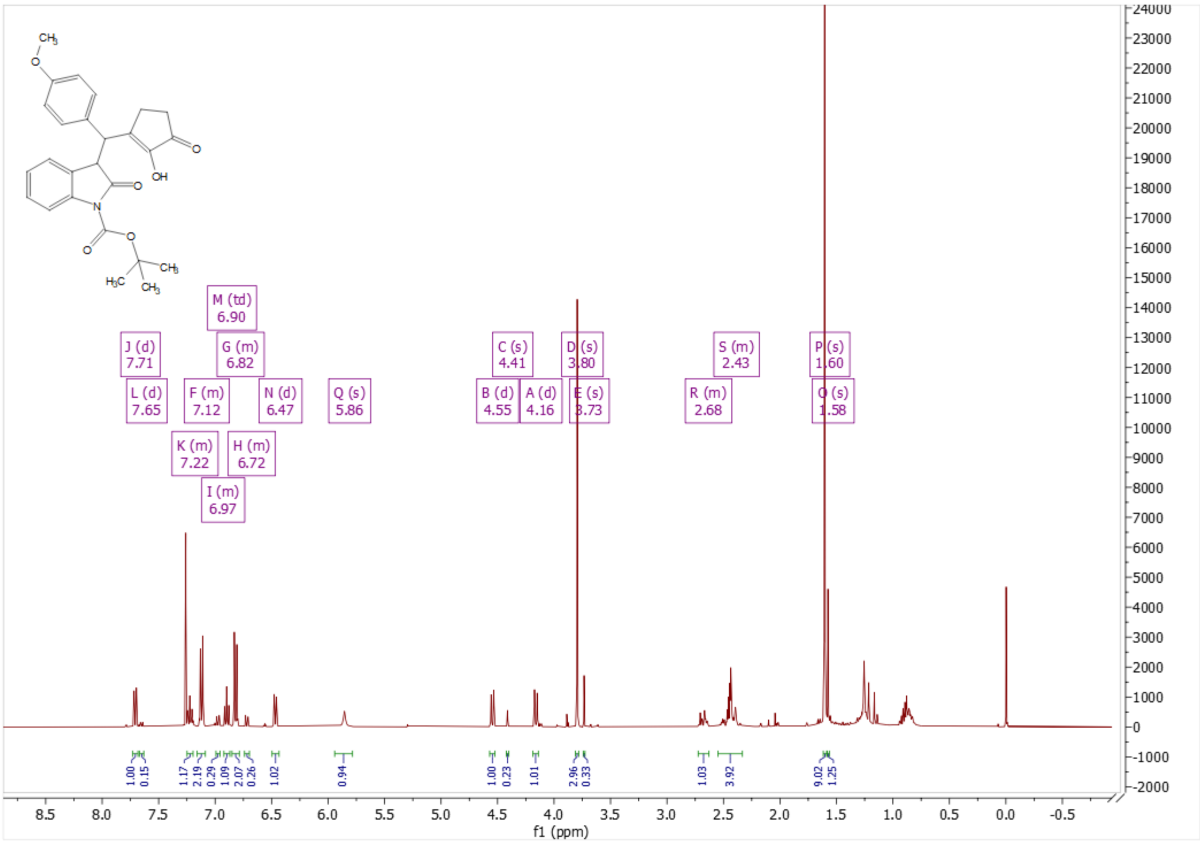

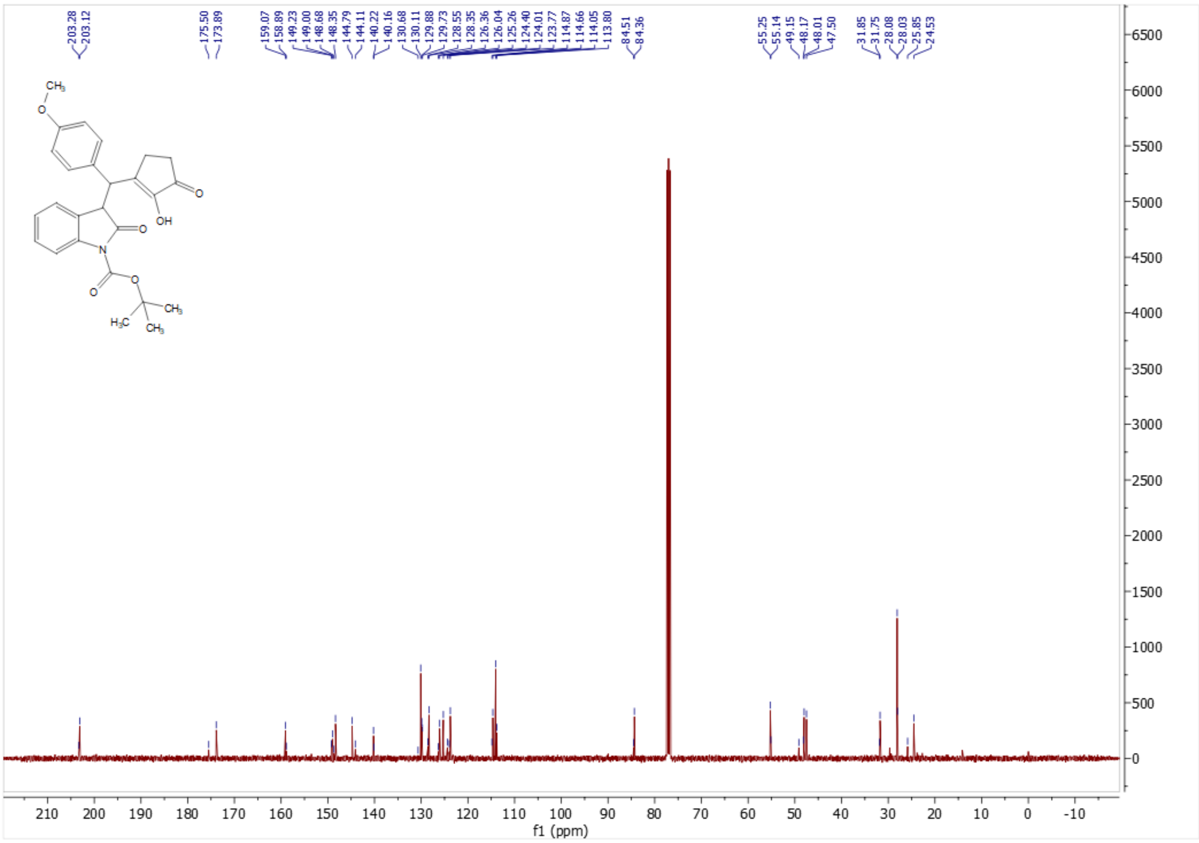

tert-Butyl 3-((2-hydroxy-3-oxocyclopent-1-en-1-yl)(*p*-tolyl)methyl)-2-oxoindoline-1-carboxylate (**3n**), mixture of diastereoisomers, ^{1}H, ^{13}C NMR

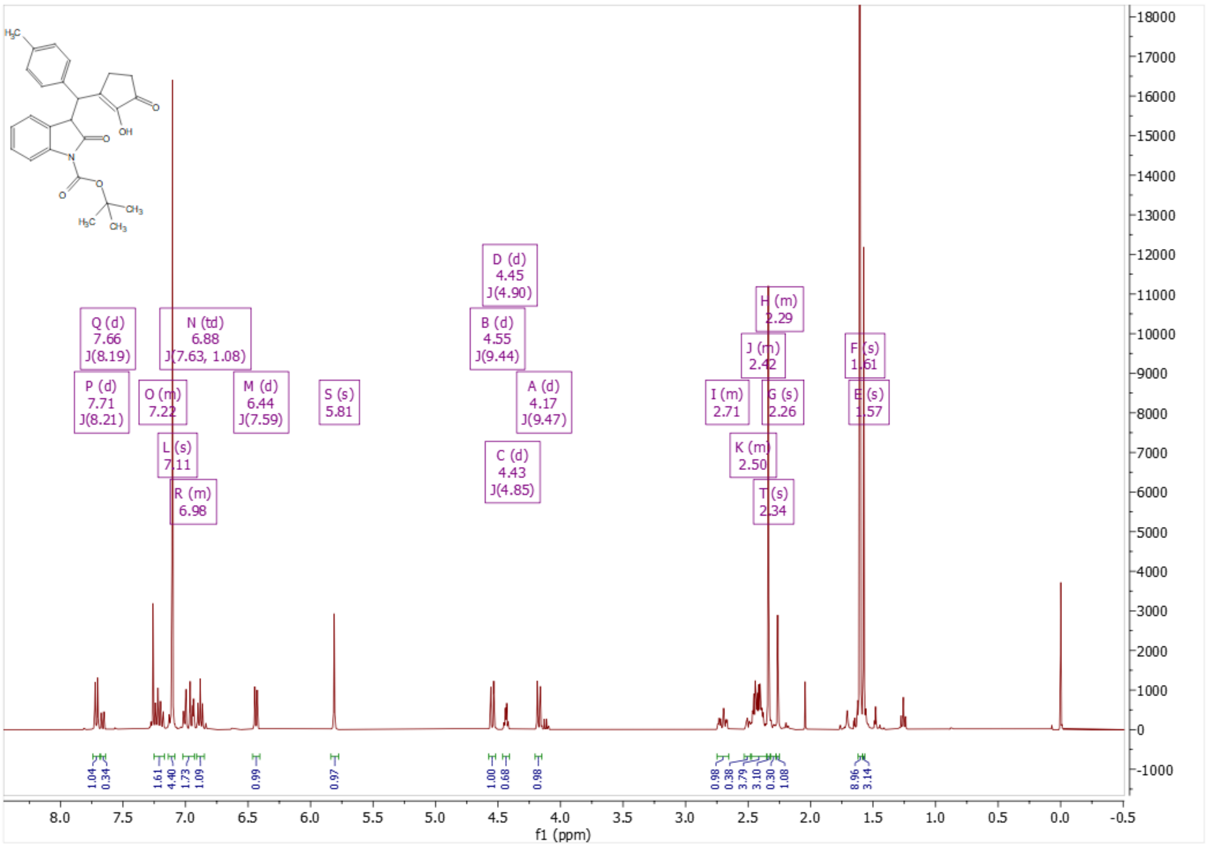

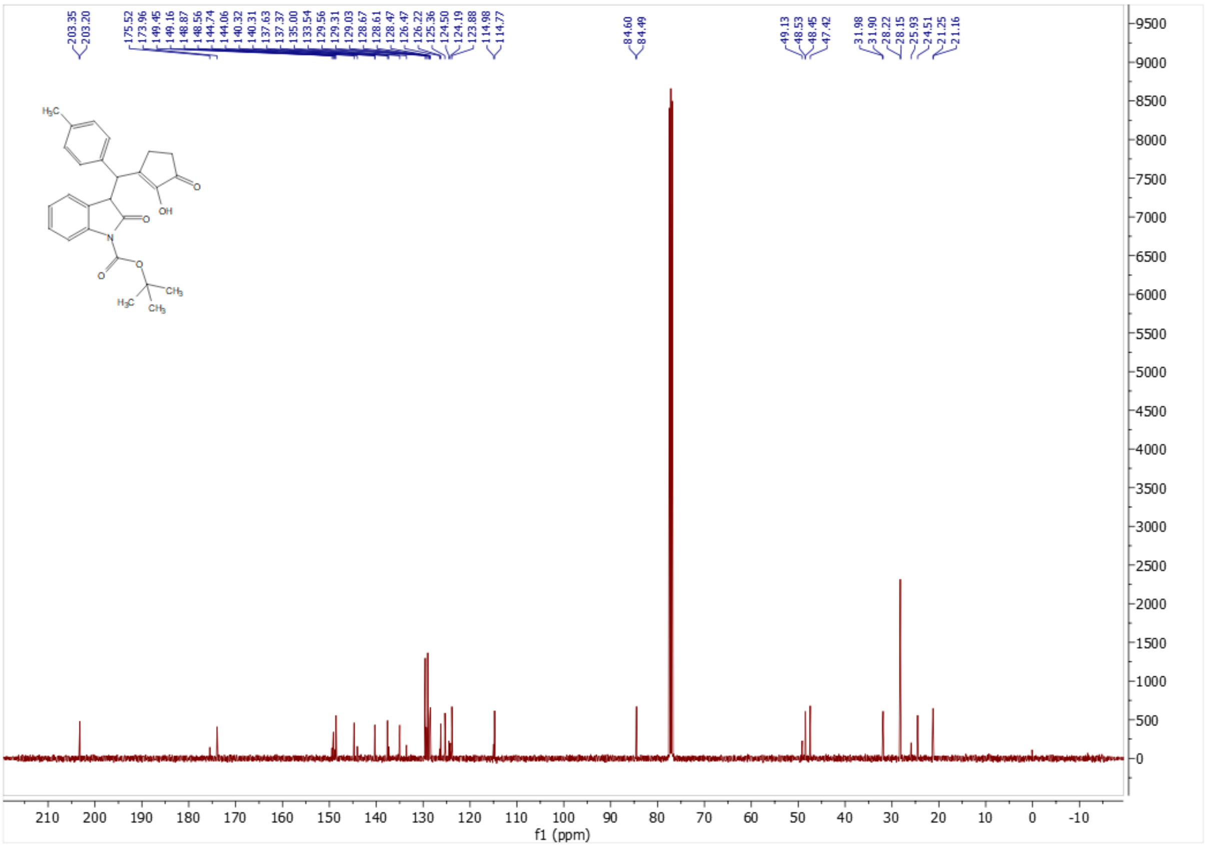

tert-Butyl 3-(1-(2-hydroxy-3-oxocyclopent-1-en-1-yl)pentyl)-2-oxoindoline-1-carboxylate (**3o**), mixture of diastereoisomers, ^{1}H, ^{13}C NMR

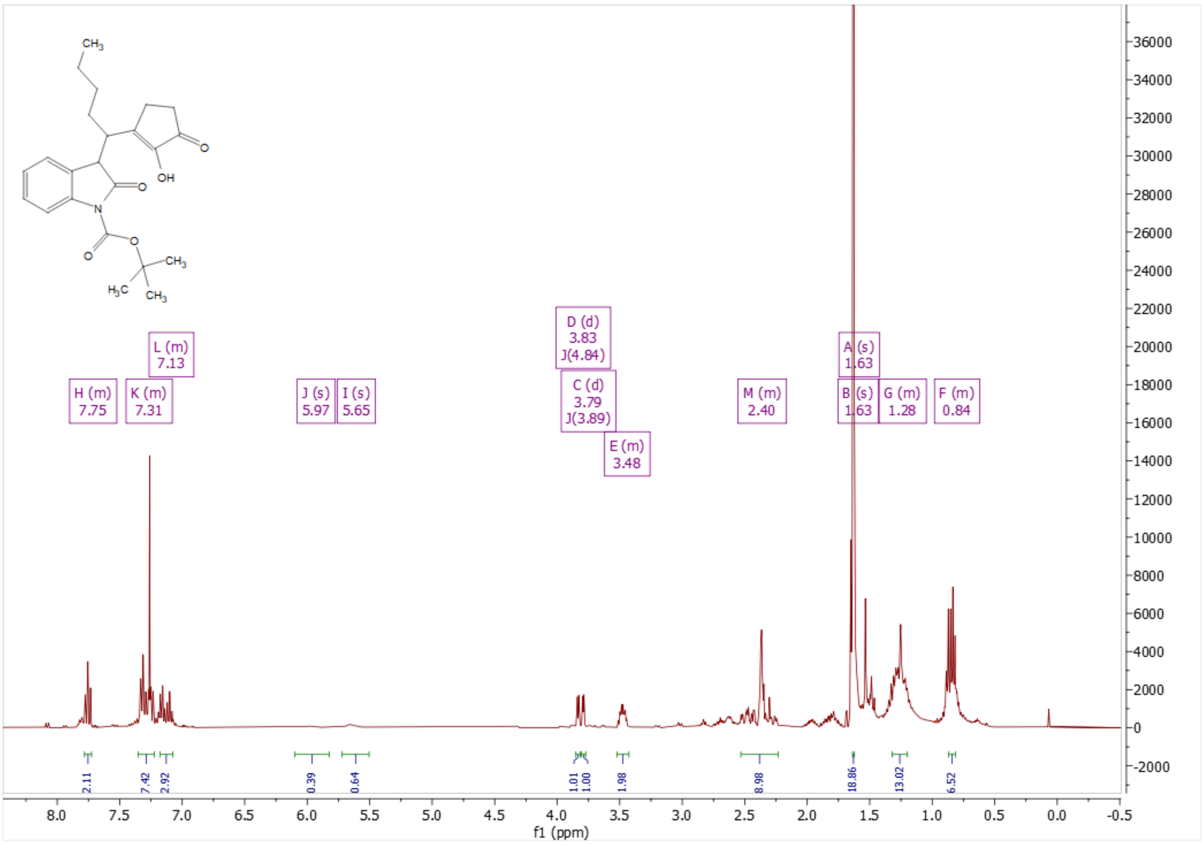

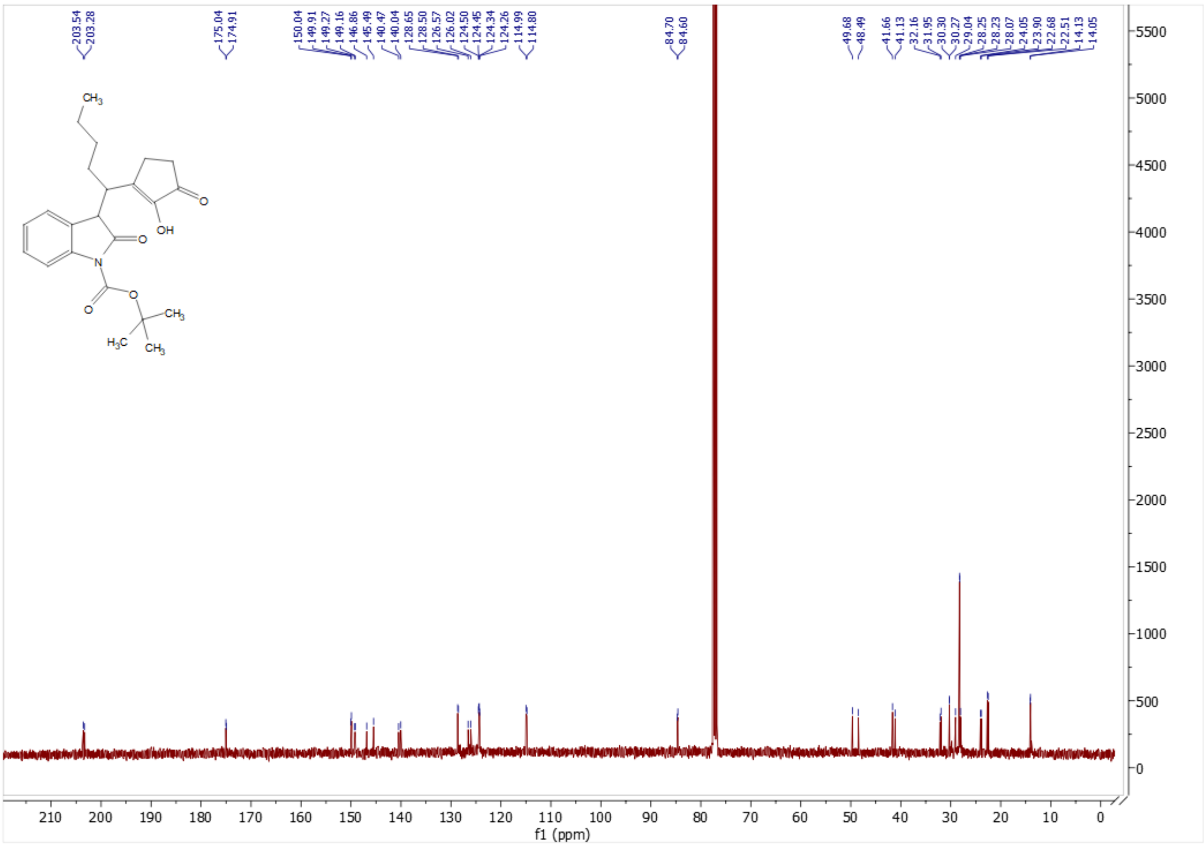

tert-Butyl 3-((2-hydroxy-3-oxocyclopent-1-en-1-yl)(3-nitrophenyl)methyl)-2-oxoindoline-1-carboxylate (**3q**), mixture of diastereoisomers, ^{1}H, ^{13}C NMR

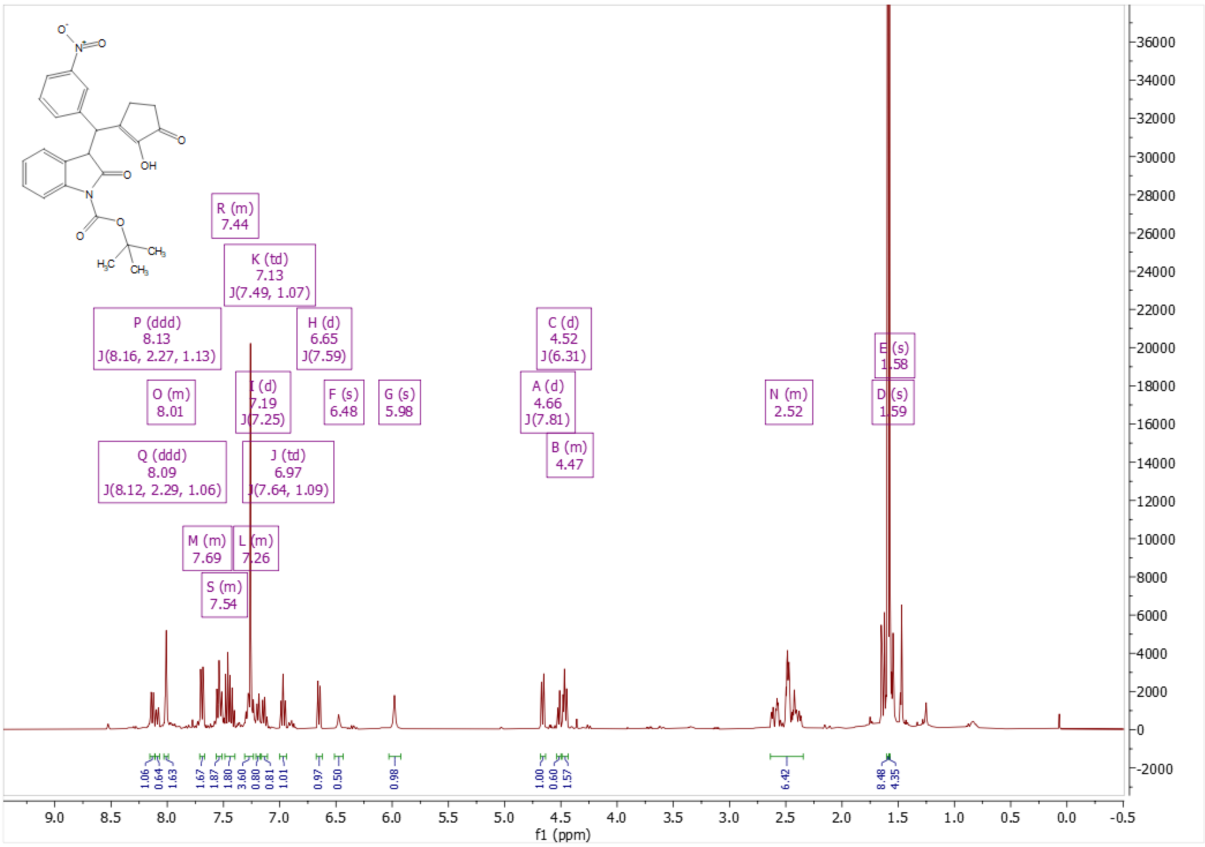

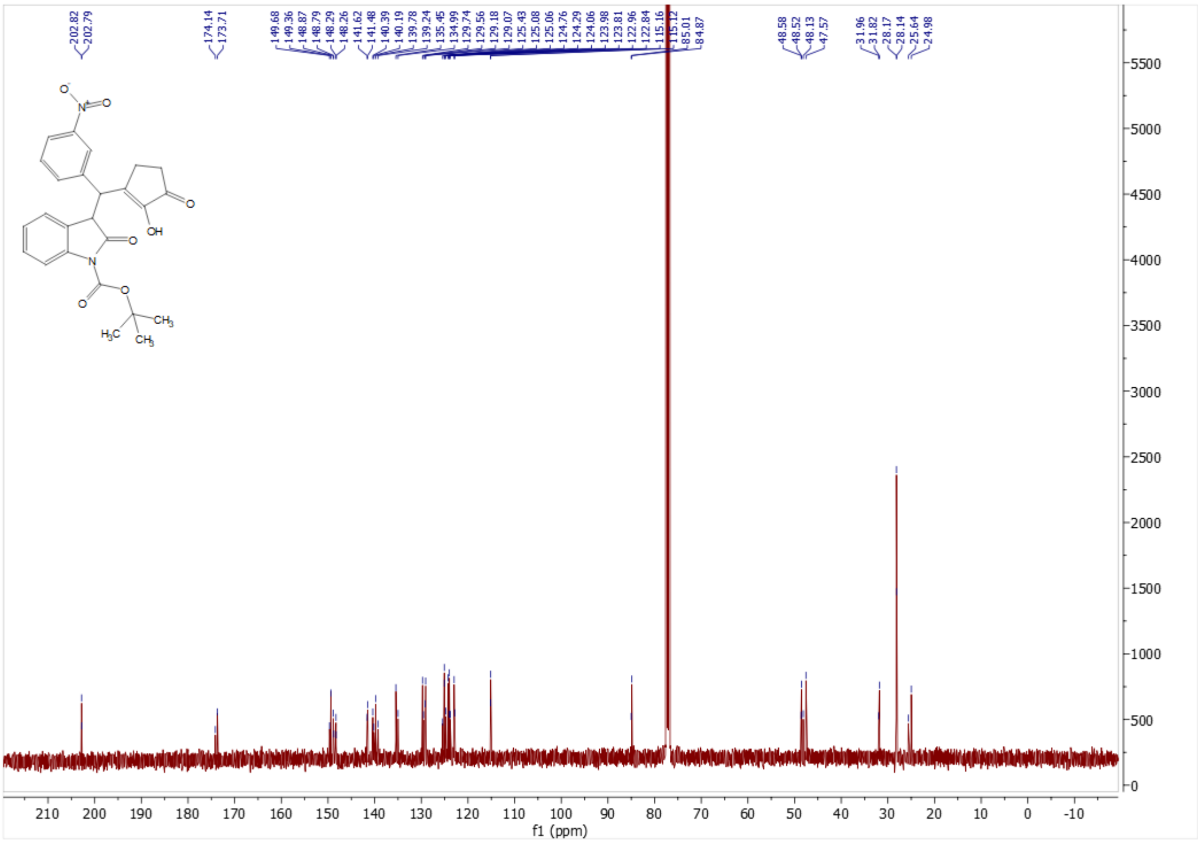

3. HPLC chromatograms

tert-Butyl 3-((2-hydroxy-3-oxocyclopent-1-en-1-yl)(phenyl)methyl)-2-oxoindolin-1-carboxylate (**3a**) HPLC chromatograms

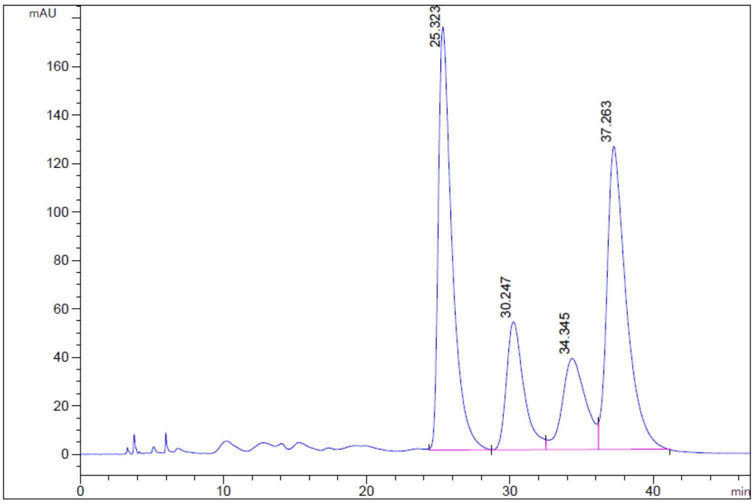

Signal 1: VWD1 A, Wavelength=254 nm

Peak #	RT [min]	Type	Width [min]	Area	Area %	Name
1	25.323	MF	1.109	11626.188	36.261	
2	30.247	MF	1.407	4455.152	13.895	
3	34.345	FM	1.894	4265.373	13.303	
4	37.263	FM	1.562	11716.183	36.541	

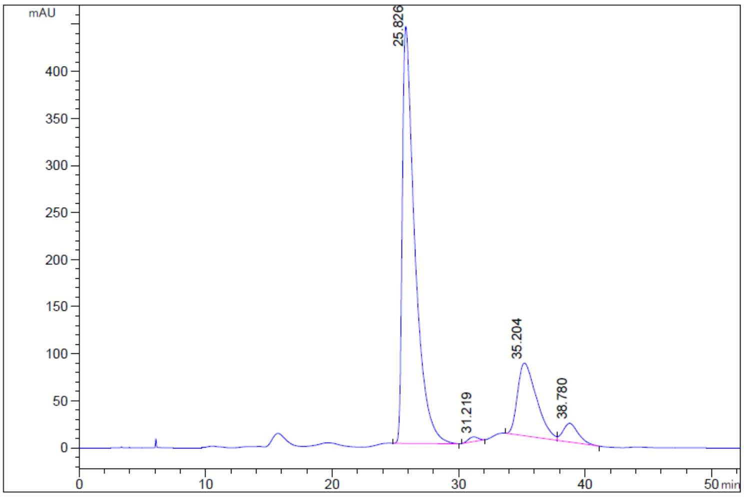

Signal 1: VWD1 A, Wavelength=254 nm

Peak #	RT [min]	Type	Width [min]	Area	Area %	Name
1	25.826	BB	1.013	30561.203	75.349	
2	31.219	BB	0.825	263.947	0.651	
3	35.204	BV	1.568	8079.364	19.920	
4	38.780	VB	1.234	1655.200	4.081	

Benzyl 3-((2-hydroxy-3-oxocyclopent-1-en-1-yl)(phenyl)methyl)-2-oxoindoline-1-carbocylate (**3b**) HPLC chromatograms

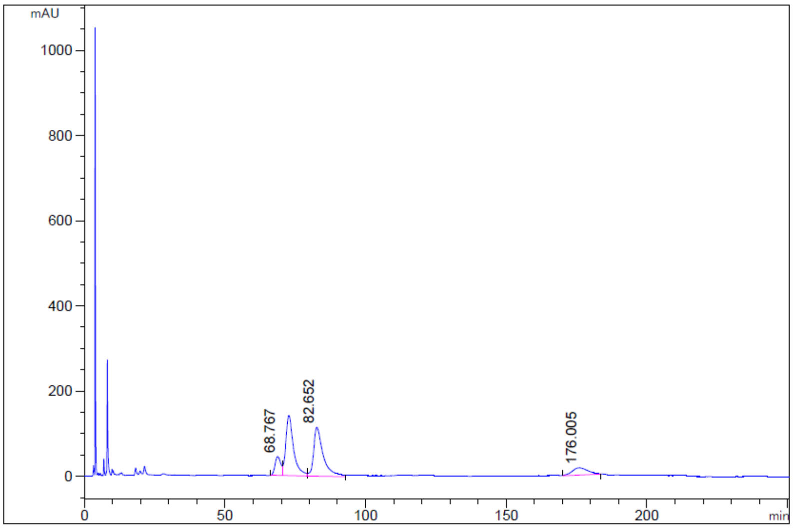

Signal 1: VWD1 A, Wavelength=210 nm

Peak #	RT [min]	Type	Width [min]	Area	Area %	Name
1	68.767	MF	2.363	6275.033	9.330	
2	72.703	FM	3.201	27119.754	40.325	
3	82.652	FM	3.951	27208.939	40.457	
4	176.005	MM	6.444	6649.613	9.887	

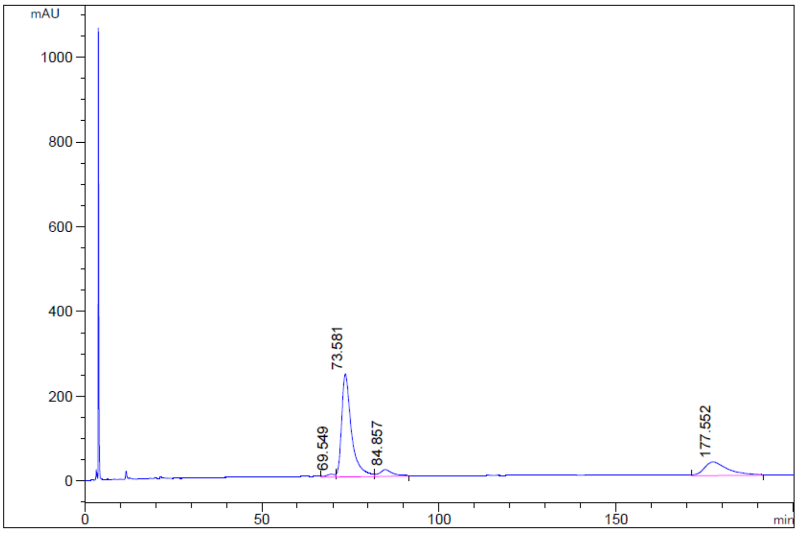

Signal 1: VWD1 A, Wavelength=210 nm

Peak #	RT [min]	Type	Width [min]	Area	Area %	Name
1	69.549	MF	2.926	1238.882	1.835	
2	73.581	FM	3.208	46846.590	69.376	
3	84.857	FM	4.265	4195.224	6.213	
4	177.552	MM	7.903	15244.678	22.576	

(9*H*-Fluoren-9-yl)methyl 3-((2-hydroxy-3-oxocyclopent-1-en-1-yl)(phenyl)methyl)-2-oxoindoline-1-carboxylate (**3c**) HPLC chromatograms

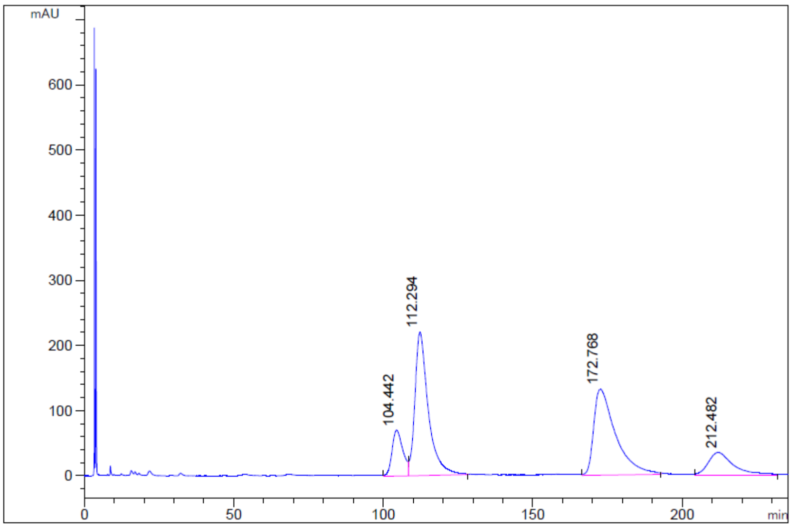

Signal 1: VWD1 A, Wavelength=210 nm

Peak #	RT [min]	Type	Width [min]	Area	Area %	Name
1	104.442	MF	4.203	17895.303	10.188	
2	112.294	FM	5.192	68678.016	39.098	
3	172.768	MM	8.492	67491.508	38.422	
4	212.482	MM	10.151	21591.742	12.292	

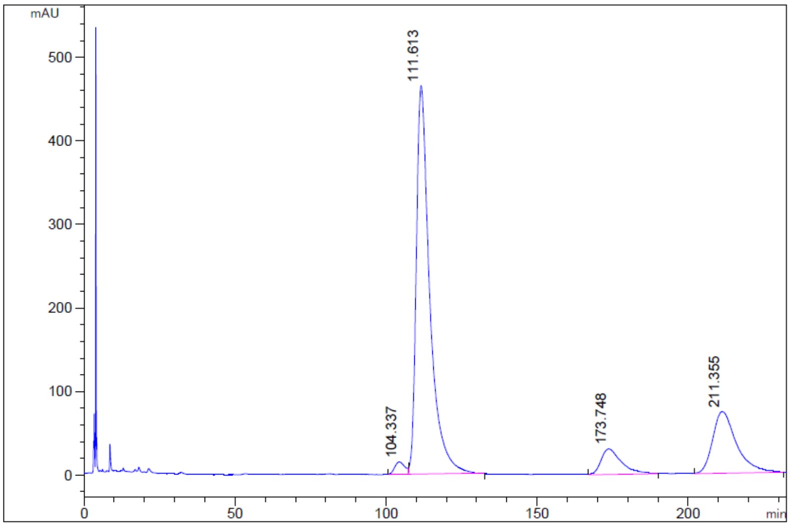

Signal 1: VWD1 A, Wavelength=210 nm

Peak #	RT [min]	Type	Width [min]	Area	Area %	Name
1	104.337	MF	3.872	3433.465	1.699	
2	111.613	FM	5.148	143488.219	71.007	
3	173.748	MM	7.868	14513.068	7.182	
4	211.355	MM	9.150	40641.004	20.112	

tert-Butyl 3-((2-chlorophenyl)(2-hydroxy-3-oxocyclopent-1-en-1-yl)methyl)-2-oxoindoline-1-carboxylate (**3f**) HPLC chromatograms

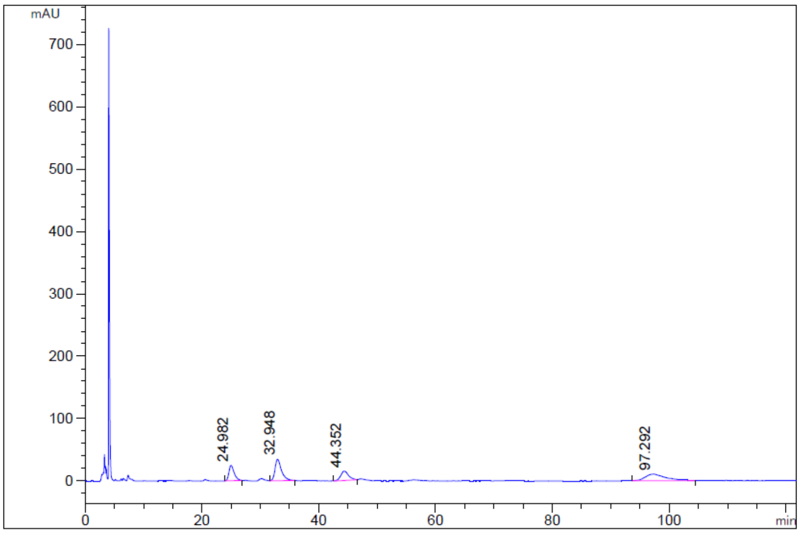

Signal 1: VWD1 A, Wavelength=210 nm

Peak #	RT [min]	Type	Width [min]	Area	Area %	Name
1	24.982	MM	1.047	1545.182	18.602	
2	32.948	MM	1.287	2663.619	32.066	
3	44.352	MM	1.591	1434.078	17.264	
4	97.292	MM	4.139	2663.830	32.068	

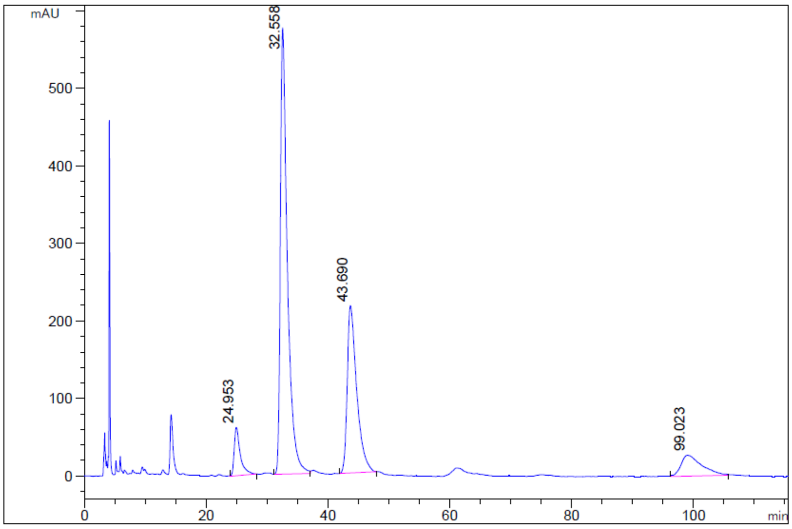

Signal 1: VWD1 A, Wavelength=210 nm

Peak #	RT [min]	Type	Width [min]	Area	Area %	Name
1	24.953	MM	1.153	4312.792	5.365	
2	32.558	BV	1.151	46278.965	57.574	
3	43.690	BB	1.504	23359.273	29.060	
4	99.023	MM	3.967	6430.982	8.001	

tert-Butyl 3-((3-chlorophenyl)(2-hydroxy-3-oxocyclopent-1-en-1-yl)methyl)-2-oxoindoline-1-carboxylate (**3g**) HPLC chromatograms

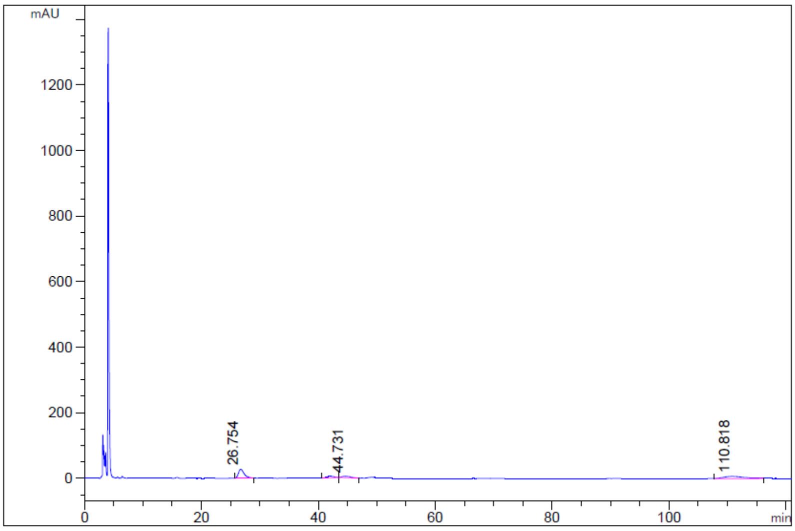

Signal 1: VWD1 A, Wavelength=210 nm

Peak #	RT [min]	Type	Width [min]	Area	Area %	Name
1	26.754	BB	1.003	1788.785	41.151	
2	42.026	BB	1.034	385.484	8.868	
3	44.731	BB	1.193	402.538	9.260	
4	110.818	MM	4.384	1770.079	40.721	

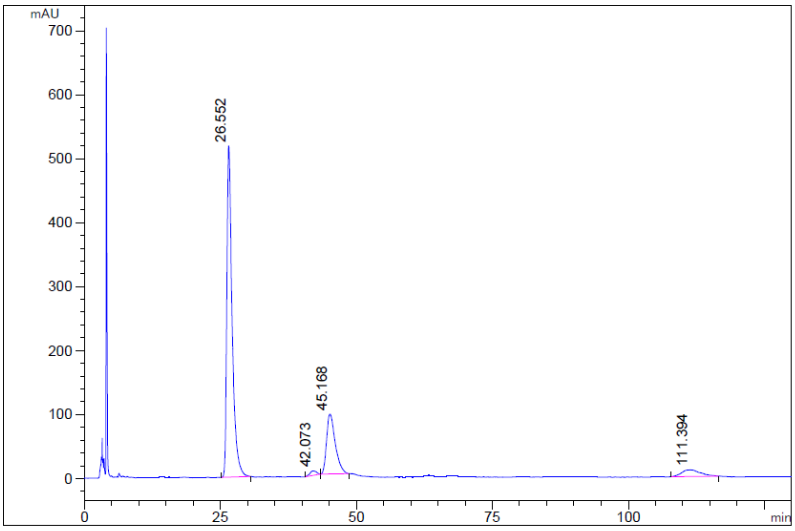

Signal 1: VWD1 A, Wavelength=210 nm

Peak #	RT [min]	Type	Width [min]	Area	Area %	Name
1	26.552	BB	0.995	35140.191	72.288	
2	42.073	BB	1.001	566.907	1.166	
3	45.168	BB	1.627	10412.548	21.420	
4	111.394	BB	2.807	2491.873	5.126	

tert-Butyl 3-((4-chlorophenyl)(2-hydroxy-3-oxocyclopent-1-en-1-yl)methyl)-2-oxoindoline-1-carboxylate (**3h**) HPLC chromatograms

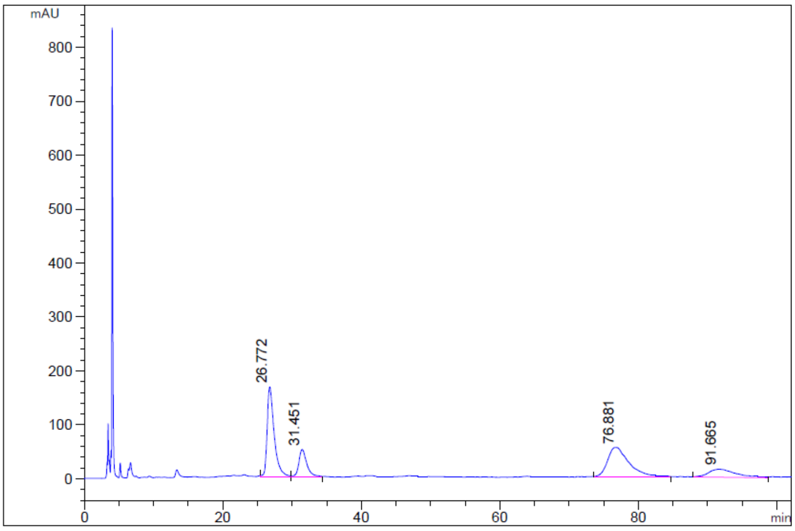

Signal 1: VWD1 A, Wavelength=210 nm

Peak #	RT [min]	Type	Width [min]	Area	Area %	Name
1	26.772	MF	1.187	11864.861	36.883	
2	31.451	FM	1.355	4150.425	12.902	
3	76.881	MM	3.637	11909.096	37.021	
4	91.665	MM	4.709	4244.223	13.194	

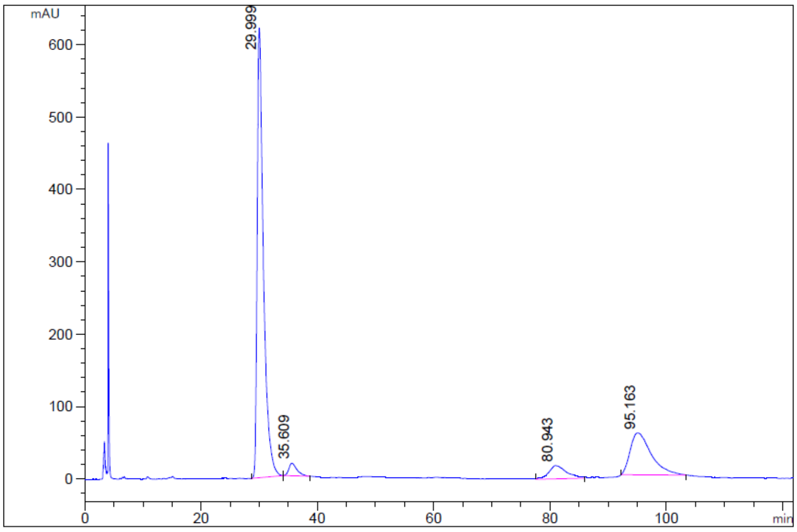

Signal 1: VWD1 A, Wavelength=210 nm

Peak #	RT [min]	Type	Width [min]	Area	Area %	Name
1	29.999	BB	1.103	46240.594	69.608	
2	35.609	BB	1.344	1660.336	2.499	
3	80.943	MM	3.564	3944.380	5.938	
4	95.163	BB	3.006	14584.229	21.954	

tert-Butyl 3-(2-ethoxy-1-(2-hydroxy-3-oxocyclopent-1-en-1-yl)-2-oxoethyl)-2-oxoindoline-1-carboxylate (**3i**) HPLC chromatograms

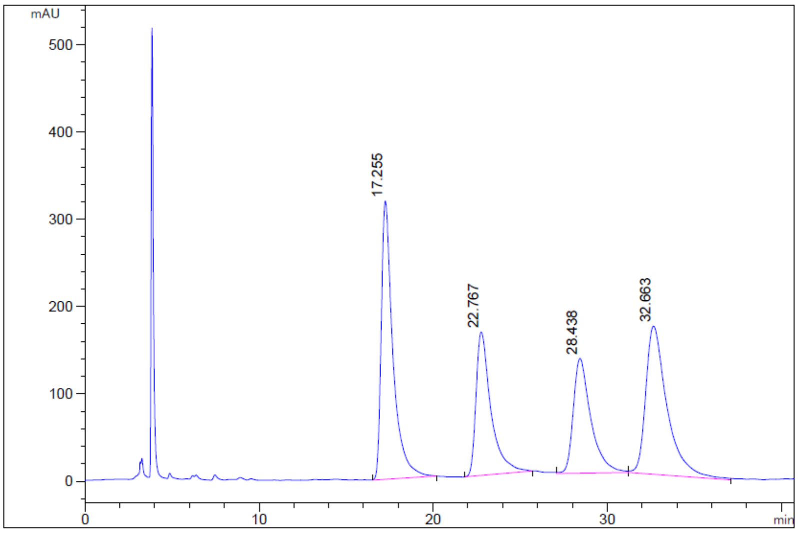

Signal 1: VWD1 A, Wavelength=210 nm

Peak #	RT [min]	Type	Width [min]	Area	Area %	Name
1	17.255	MM	0.776	14833.880	30.715	
2	22.767	BB	0.874	9774.354	20.239	
3	28.438	MM	1.170	9230.505	19.113	
4	32.663	MM	1.419	14455.896	29.933	

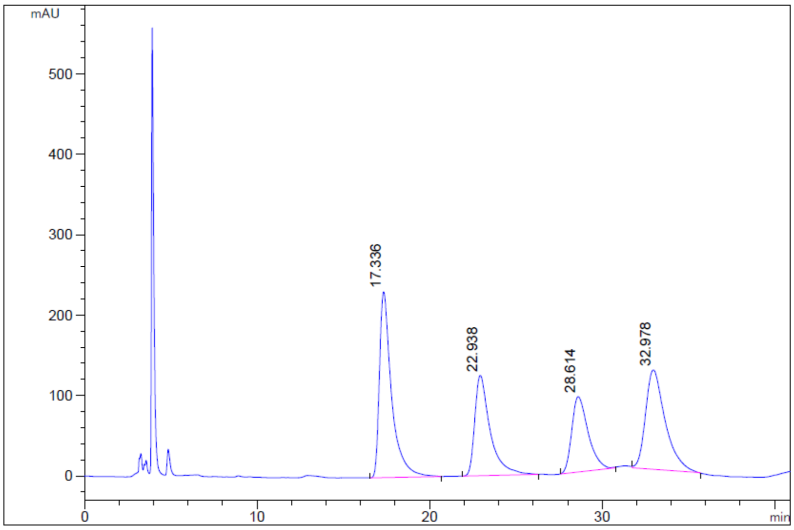

Signal 1: VWD1 A, Wavelength=210 nm

Peak #	RT [min]	Type	Width [min]	Area	Area %	Name
1	17.336	BB	0.715	11358.809	32.414	
2	22.938	BB	0.903	7749.295	22.114	
3	28.614	MM	1.097	6157.303	17.571	
4	32.978	MM	1.319	9777.777	27.902	

tert-Butyl 3-((2-hydroxy-3-oxocyclopent-1-en-1-yl)(thiophen-2-yl)methyl)-2-oxoindoline-1-carboxylate (**3j**) HPLC chromatogram

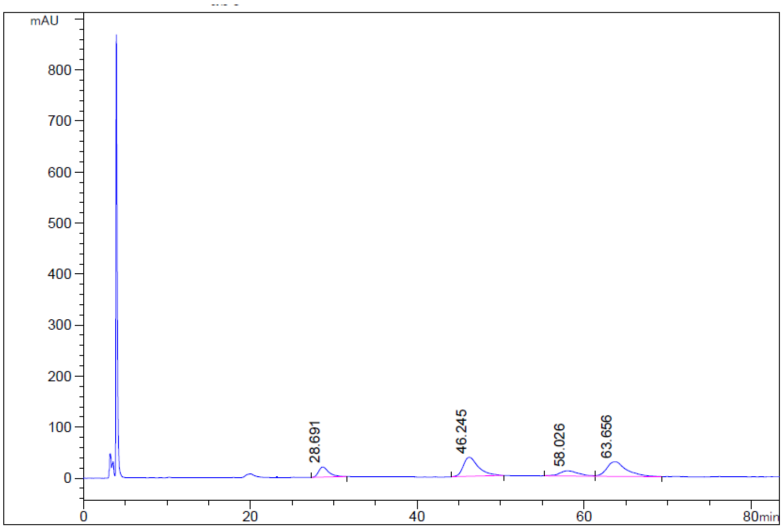

Signal 1: VWD1 A, Wavelength=210 nm

Peak #	RT [min]	Type	Width [min]	Area	Area %	Name
1	28.691	BB	1.177	1673.310	12.998	
2	46.245	BB	1.663	4730.728	36.747	
3	58.026	MF	2.705	1666.726	12.947	
4	63.656	FM	2.787	4803.116	37.309	

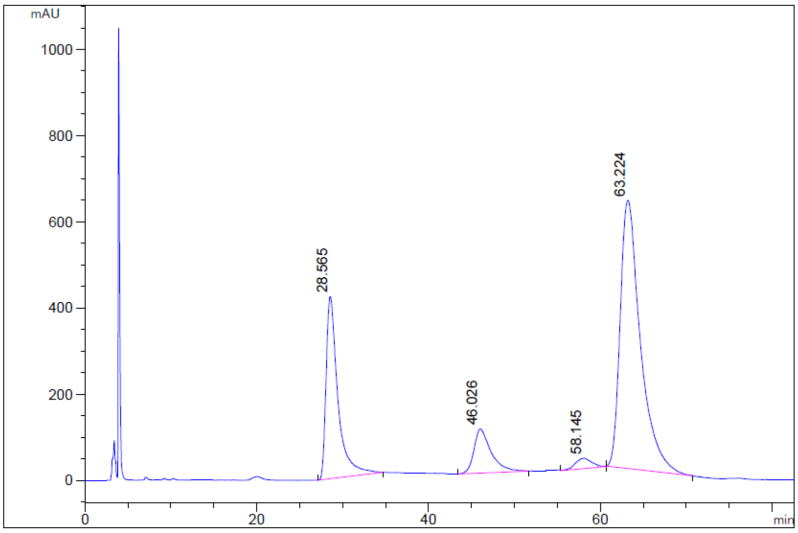

Signal 1: VWD1 A, Wavelength=210 nm

Peak #	RT [min]	Type	Width [min]	Area	Area %	Name
1	28.565	BB	1.393	40446.363	25.391	
2	46.026	BB	1.977	14228.920	8.932	
3	58.145	MM	2.215	3167.262	1.988	
4	63.224	MM	2.717	101452.188	63.688	

tert-Butyl 5-bromo-3-((2-hydroxy-3-oxocyclopent-1-en-1-yl)(phenyl)methyl)-2-oxoindoline-1-carboxylate (**3k**) HPLC chromatograms

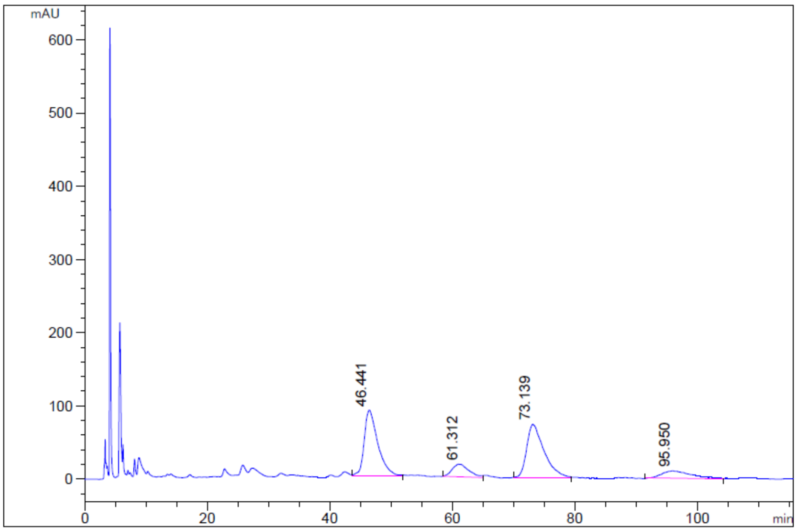

Signal 1: VWD1 A, Wavelength=210 nm

Peak #	RT [min]	Type	Width [min]	Area	Area %	Name
1	46.441	MM	2.574	13916.131	40.044	
2	61.312	MM	3.169	3227.537	9.287	
3	73.139	MM	3.258	14197.988	40.855	
4	95.950	MM	5.587	3410.850	9.815	

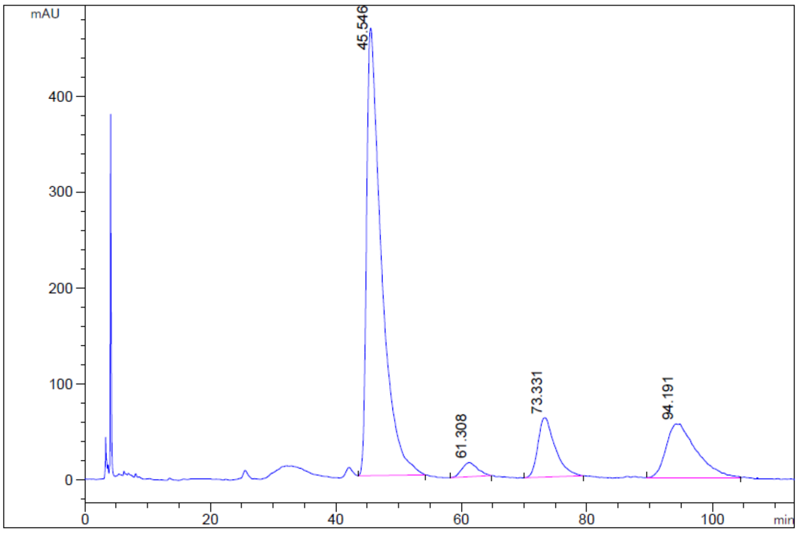

Signal 1: VWD1 A, Wavelength=210 nm

Peak #	RT [min]	Type	Width [min]	Area	Area %	Name
1	45.546	BB	2.249	77108.336	70.073	
2	61.308	BB	2.074	2533.630	2.302	
3	73.331	BB	2.302	11790.827	10.715	
4	94.191	MM	5.559	18607.006	16.909	

tert-Butyl 4-bromo-3-((2-hydroxy-3-oxocyclopent-1-en-1-yl)(phenyl)methyl)-2-oxoindoline-1-carboxylate (**3l**) HPLC chromatograms

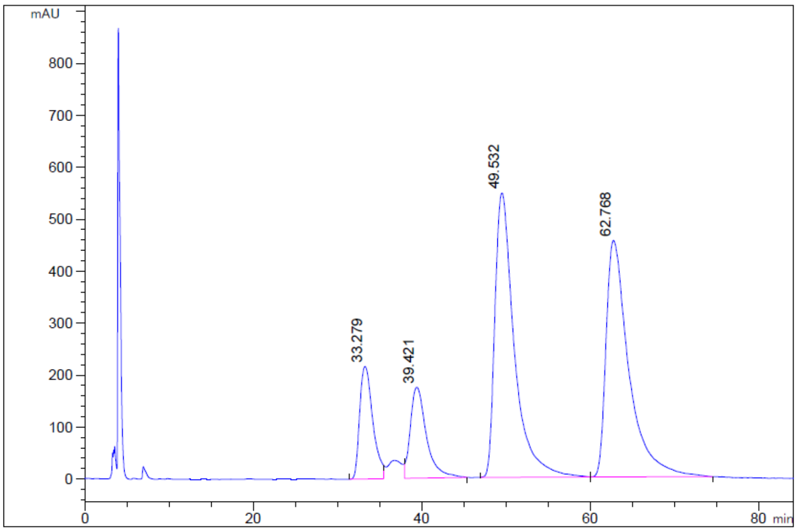

Signal 1: VWD1 A, Wavelength=210 nm

Peak #	RT [min]	Type	Width [min]	Area	Area %	Name
1	33.279	MF	1.807	23468.719	10.639	
2	39.421	FM	2.213	23240.381	10.536	
3	49.532	BB	2.309	87867.836	39.834	
4	62.768	BB	2.536	86008.125	38.991	

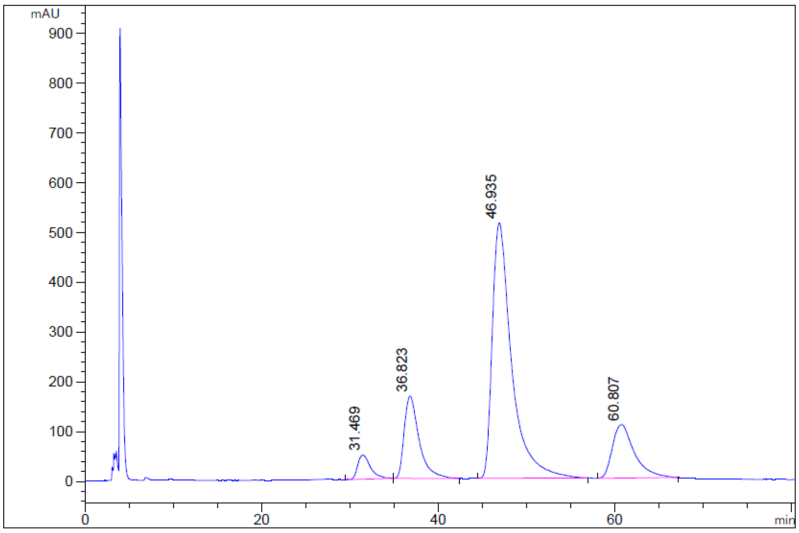

Signal 1: VWD1 A, Wavelength=210 nm

Peak #	RT [min]	Type	Width [min]	Area	Area %	Name
1	31.469	BB	1.526	4883.122	4.028	
2	36.823	BB	1.733	19390.889	15.995	
3	46.935	BB	2.030	78325.977	64.610	
4	60.807	BB	2.423	18628.213	15.366	

tert-Butyl 3-((2-hydroxy-3-oxocyclopent-1-en-1-yl)(*p*-tolyl)methyl)-2-oxoindoline-1-carboxylate (**3n**) HPLC chromatograms

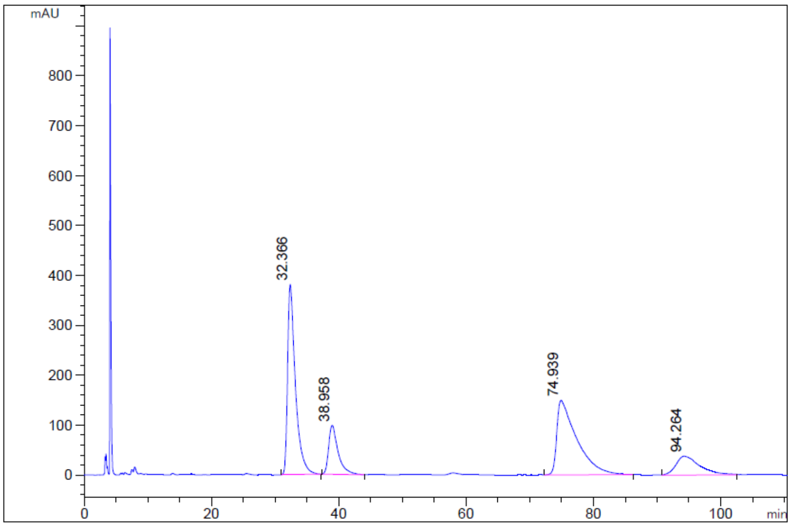

Signal 1: VWD1 A, Wavelength=210 nm

Peak #	RT [min]	Type	Width [min]	Area	Area %	Name
1	32.366	MM	1.426	32619.086	38.210	
2	38.958	MM	1.734	10225.687	11.978	
3	74.939	MM	3.630	32584.193	38.169	
4	94.264	MM	4.359	9938.545	11.642	

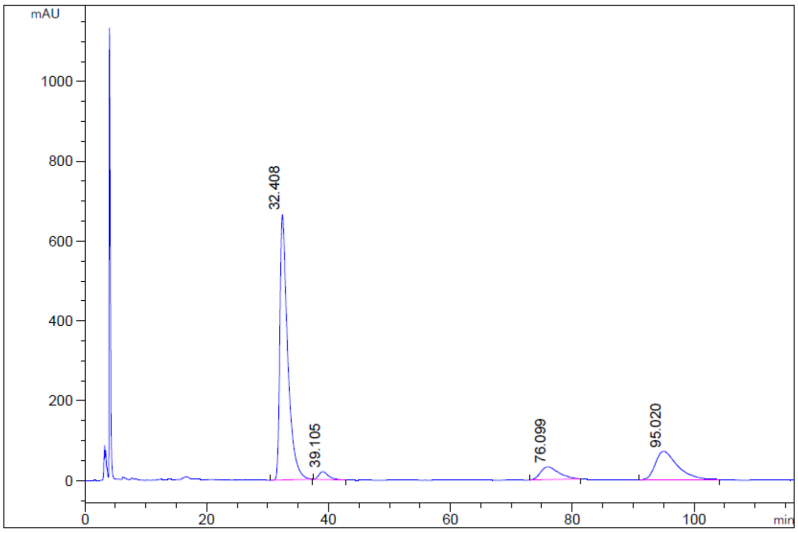

Signal 1: VWD1 A, Wavelength=210 nm

Peak #	RT [min]	Type	Width [min]	Area	Area %	Name
1	32.408	BB	1.289	59425.695	68.654	
2	39.105	BB	1.392	2166.220	2.503	
3	76.099	MM	3.473	6739.010	7.786	
4	95.020	MM	4.258	18226.926	21.058	

tert-Butyl 3-(1-(2-hydroxy-3-oxocyclopent-1-en-1-yl)pentyl)-2-oxoindoline-1-carboxylate (**3o**) HPLC chromatograms

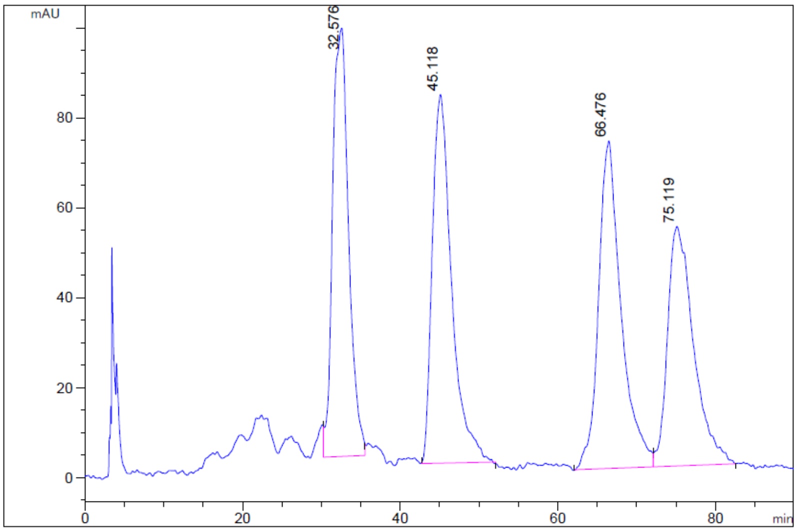

Signal 1: VWD1 A, Wavelength=210 nm

Peak #	RT [min]	Type	Width [min]	Area	Area %	Name
1	32.576	FM	2.357	13469.322	24.845	
2	45.118	MM	2.847	14002.417	25.829	
3	66.476	MF	3.319	14501.938	26.750	
4	75.119	FM	3.829	12239.016	22.576	

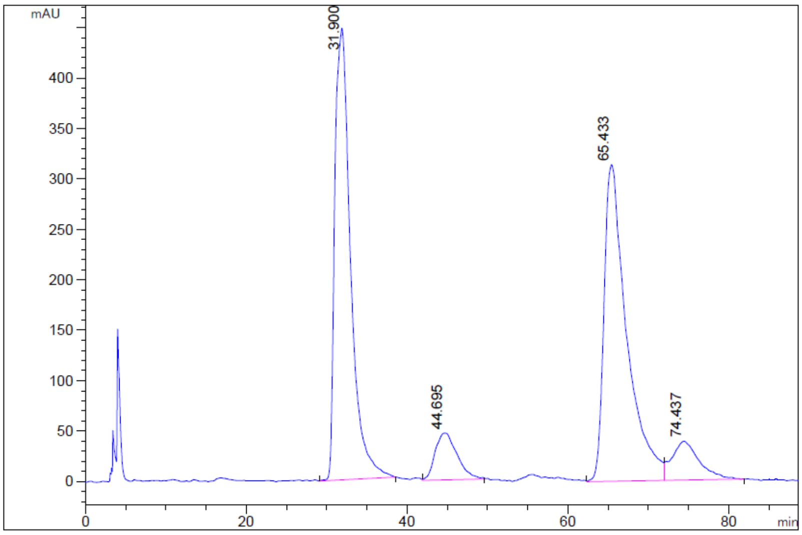

Signal 1: VWD1 A, Wavelength=210 nm

Peak #	RT [min]	Type	Width [min]	Area	Area %	Name
1	31.900	BB	1.852	60917.180	43.431	
2	44.695	MM	3.169	8807.506	6.279	
3	65.433	MF	3.262	61424.047	43.793	
4	74.437	FM	3.949	9112.127	6.497	

tert-Butyl 3-((2-hydroxy-3-oxocyclopent-1-en-1-yl)(3-nitrophenyl)methyl)-2-oxoindoline-1-carboxylate (**3q**) HPLC chromatograms

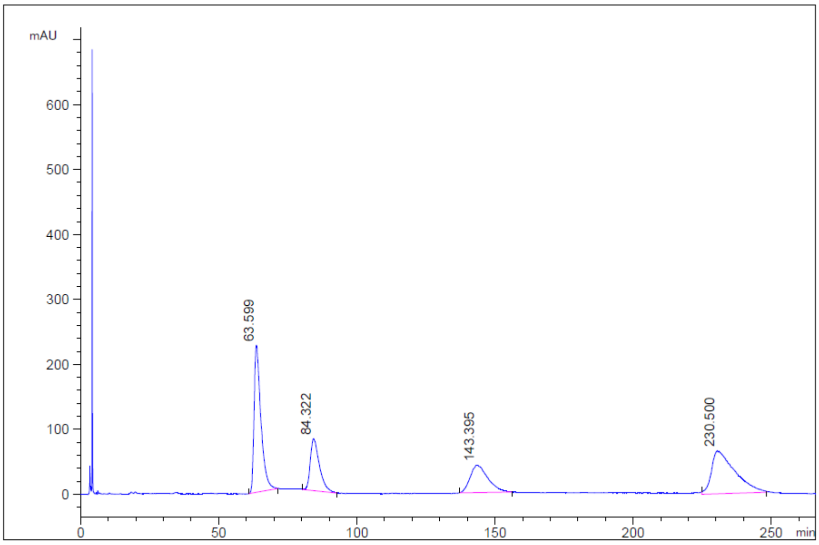

Signaal 1: VWD1 A, Wavelength=210 nm

Peak	Time [min]	Type	With [min]	Area	Area %	Name
1	63.599	BB	2.344	3.899e4	33.907	
2	84.322	MM	3.824	1.834e4	15.951	
3	143.395	MM	7.485	1.901e4	16.529	
4	230.500	MM	9.703	3.865e4	33.612	

From **(*E*)-2q**

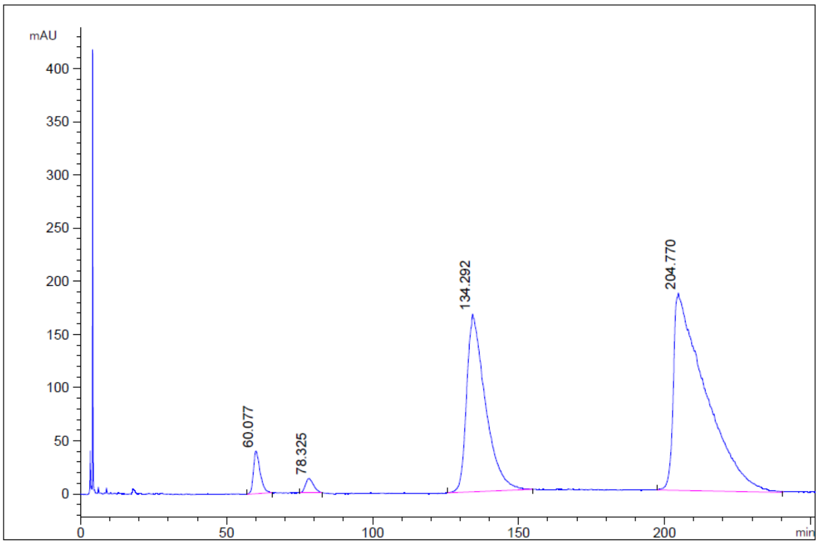

Signaal 1: VWD1 A, Wavelength=210 nm

Peak	Time [min]	Type	With [min]	Area	Area %	Name
1	60.077	MM	2.664	6413.939	2.862	
2	78.325	MM	3.224	2626.680	1.172	
3	134.292	MM	7.409	7.429e4	33.147	
4	204.770	MM	12.634	1.408e5	62.819	

From ***(Z)*-2q'**

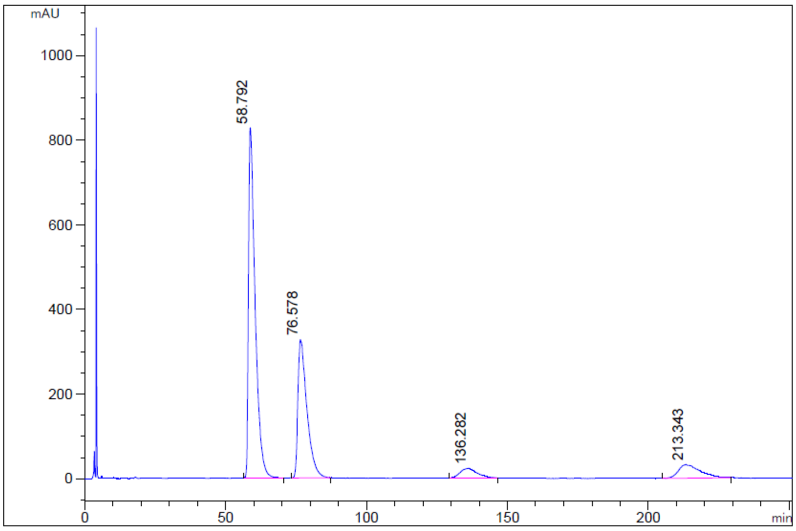

Signal 1: VWD1 A, Wavelength=210 nm

Peak #	RT [min]	Type	Width [min]	Area	Area %	Name
1	58.792	MM	2.733	135698.922	57.679	
2	76.578	MM	3.724	72985.477	31.023	
3	136.282	MM	6.815	9232.436	3.924	
4	213.343	MM	8.977	17348.877	7.374	

References

1. Wrobel, J.; Cook, J. M. *Synth. Commun.* **1980**, *10*, 333-337.
2. Rehan, M.; Flegel, J.; Hetikamp, F.; Pergomet, J. L.; Otte, F.; Strochmann, C.; Kumar, K. **2020**, *52*, 3140-3152.
3. Wang, G.; Liu, X.; Huang, T.; Kuang, Y.; Lin, L.; Feng, X. *Org. Lett*. **2013**, *15*, 76-79.
4. Ghosh, A. K.; Zhou, B. *Tetrahedron Lett.* **2013**, *51*, 2311-2314.
5. Bae, H. Y.; Some, S.; Lee, J. H.; Kim, J.-Y.; Song, M. J.; Lee, S.; Zhang, Y. J.; Song, C. E. *Adv. Synth. Catal.* **2011**, *353*, 3196-3202.
6. Manna, M. S.; Mukherjee, S. *J. Am. Chem. Soc.* **2015**, *137*, 130-133.
7. Rao, K. S.; Ramesh, P.; Trivedi, R.; Kantam, M. L. *Tetrahedron Lett.* **2016**, *57*, 1227-1231.
8. Vakulya, B.; Varga, S.; Csámpai, A.; Soós, T. *Org. Lett.* **2005**, *7*, 1967-1969.
